# Conditioning Public Opinion Perceptions by “Survey Methods 101”: Informing, Engaging, and Motivating Individuals for Critical Processing of Public Opinion Polls

**DOI:** 10.1093/poq/nfag006

**Published:** 2026-03-22

**Authors:** Ozan Kuru

**Affiliations:** Assistant Professor, Department of Communications and New Media, National University of Singapore, Singapore

## Abstract

Can we train individuals on survey methods to boost their critical processing of public opinion evidence? While polls are one of the most systematic and scientific methods for measuring and communicating public opinion, they face credibility challenges, such as decreasing public trust, the prevalence of straw and misinformation polls, and individuals’ biased dismissal of polls that deliver unfavorable results. These issues manifest and fuel misperceptions, misinformation, and polarization in public opinion, pollsters, and the media. We designed and tested a novel strategy to mitigate these challenges collectively, indirectly, and nonconfrontationally via preemptive educative interventions that cultivate polling literacy. Integrating components from pedagogical and persuasion theories, three interventions are designed to (1) inform (passive literacy), (2) inform and engage (active literacy), and (3) inform, engage, and motivate (psychological inoculation) individuals. The effects of these interventions were evaluated and compared in an extensive, preregistered, longitudinal experiment. In Wave 1, trainees were exposed to one of the interventions or a control training. In Wave 2, participants viewed polls with either poor or robust methodology, tested across different poll results (majority supporting vs. opposing; ecological validity) and issues (COVID-19 vaccines and artificial intelligence; conceptual replication), and then evaluated polling evidence. Results showed that inoculation was particularly effective. Participants’ education levels and science literacy levels conditioned various intervention effects. The theoretical implications of this novel pathway to conditioning public opinion and practical insights are discussed with a qualitative review and recommendations for existing public education efforts.

Most corrections and interventions to mitigate and counter public opinion misperceptions and polarization are ineffective (Voelkel et al. 2023; Dias et al. 2024). We designed and tested an alternative strategy against misperceptions and, more broadly, for conditioning public opinion perceptions: *training individuals to be better consumers of public opinion evidence*. Indeed, a significant portion of misperceptions about public opinion either originate from, manifest, and/or feed the underlying confusion with, misunderstanding of, or disagreements about the systematic evidence on public opinion (Nir 2011; Kuru, Pasek, and Traugott 2017; Broockman and Skovron 2018). In this context, polls—one of the most systematic and scientific tools for measuring and communicating public opinion—face three significant credibility challenges. First, public trust in polls has been declining for reasons such as unexpected election outcomes and distrust of news media (Kim et al. 2011; Chia and Chang 2017; Bailey 2024). Second, not all polls have strong methodological quality (Baker et al. 2013; Pasek 2015). Methodological limitations may lead to distorted measurement (Westwood et al. 2022). Further, marketing-oriented push polls and social media polls may confuse the public (Stadtmüller, Silber, and Beuthner 2022; Graham, Hillygus, and Trexler 2024; Scarano et al. 2025). Such polls fuel misinformation, as seen in anti-vaccine discourse (e.g., Kirsch 2023), or can even be cited by public agencies (Kuru 2018). Third, due to motivated reasoning bias, political elites and ordinary individuals tend to discredit polls if results contradict their beliefs (Kuru, Pasek, and Traugott 2017; Madson and Hillygus 2020). These issues then reduce survey participation, erode trust in polling and media, and skew public opinion perceptions, ultimately both fueling and feeding from polarization (Chia and Chang 2017; Silber et al. 2022; Graham, Hillygus, and Trexler 2024; Johnson, Silber, and Darling 2024).

To counter these challenges in perceptions of polls collectively, indirectly, and nonconfrontationally, we investigate preemptive interventions (Kozyreva et al. 2024; Kuru 2025b) that can cultivate critical processing of political statistics. Katherine K. Wallman, the former chief statistician of the United States and the chairwoman of the UN Statistical Commission, championed the importance of the public’s statistical literacy, defining it as “the ability to understand and critically evaluate statistical results that permeate our daily lives” (Wallman 1993). The polling community should increase efforts to boost the integrity of survey research not just on the supply side but also on the public perceptions side (Traugott and Kang 2000; Groves and Lyberg 2010; Krosnick et al. 2015; Jamieson et al. 2023). Hence, our overarching research question investigates if we could train individuals on survey methods to boost their critical processing of public opinion evidence. This might be possible through brief yet substantive and scalable educative interventions that exploit strategies in intervention science (Amazeen and Krishna 2024).

We designed and tested the effectiveness of preemptive interventions to induce critical evaluation of polling evidence. By integrating insights from pedagogical and persuasion literature on literacy training (Vraga et al. 2015; Jones-Jang, Mortensen, and Liu 2021) and psychological inoculation (Compton et al. 2021; Lewandowsky and van der Linden 2021), we focused on the theoretical dimensions of informational vs. motivational components and passive vs. active engagement. These dimensions were synthesized, based on theoretical, methodological, and practical considerations, to inform the design of three interventions: (1) passive literacy (information provision), (2) active literacy (information provision + interactive engagement), and (3) psychological inoculation (information provision + interactive engagement + motivational forewarning against misleading polls). We then compared the effectiveness of these interventions on the public assessments of polls through a large-scale, longitudinal, preregistered experiment.

Participants completed one of the three interventions (or the control training) in Wave 1 (main manipulation-1). In Wave 2, trainees were exposed to polls that (1) had either poor or robust methodological quality (main manipulation-2), (2) showed majority support or opposition in the results (manipulation for ecological validity), and (3) were about COVID-19 vaccines or artificial intelligence (AI) issues (manipulation for conceptual replication). We also tested the moderating roles of a few key preexisting individual differences to investigate the differential uptake of interventions. We tested how one’s ability and confidence in processing statistics (educational attainment, science literacy, subjective numeracy) and one’s attitudes (favorability of poll results to participants’ own views) may condition (facilitate or limit) interventions’ effectiveness. We found that interventions, particularly inoculation, were effective, and individuals’ education and science literacy conditioned these effects. We discuss the theoretical contributions of this novel pathway to shaping public opinion perceptions along with a qualitative review of existing public education efforts to contextualize the practical implications.

## Existing Strategies vs. Preemptive Interventions

Understanding and improving public understanding of polls, given the lack of knowledge, misunderstandings, or motivational biases, is important given polls’ role in democracy in communicating public opinion and thus shaping political knowledge and beliefs (Lavrakas and Traugott 2000; van der Meer, Hakhverdian, and Aaldering 2016). Hence, recent research has focused on polls’ scientific/methodological characteristics as the target for promoting public understanding of polls (Kuru, Pasek, and Traugott 2017; Stadtmüller, Silber, and Beuthner 2022). This approach draws from the increasing importance of methodological transparency in the field, as reflected in AAPOR’s (2015) Transparency Initiative (AAPOR 2015; Lupia 2017). Yet, greater methodological transparency is an unused arsenal unless the public critically evaluates methodological details. Discerning high-quality and reliable polls amid a variety of poor-quality and/or misleading polls is very important, both empirically and normatively, for public perceptions of polls and public opinion (Baker et al. 2013; Pasek 2015).

Studies investigated the presence and framing of methodological details about polls, provided either simultaneously or post hoc. Findings are mixed. While the public knowledge about polls is limited (Traugott and Kang 2000), recent work found that polls with robust methodological quality are evaluated, at least by some individuals, as more credible (Kuru, Pasek, and Traugott 2020a; Stadtmüller, Silber, and Beuthner 2022). Individuals expressed greater trust in surveys with larger sample sizes and “representative” samples (Stadtmüller, Silber, and Beuthner 2022). The *sample size* was the only factor influencing journalists’ assessments of fictitious research (Bottesini et al. 2023), despite the fact that sample size alone cannot guarantee quality (Bradley et al. 2021). Finally, post hoc and direct corrections, such as expert comments on the methodology of specific polls, did not notably shift evaluations or mitigate biases (Kuru, Pasek, and Traugott 2020b).

Also, these studies are limited in a few ways. Most experiments in this context are susceptible to demand effects; participants viewed multiple polls with differing levels of methodological quality either simultaneously (e.g., relative credibility between two polls, as in Kuru, Pasek, and Traugott 2020a) or consecutively (exposure to a poll, evaluating them, and repeating this process, as in Stadtmüller et al. 2022). Such protocols might increase attention to methodological details artificially. Second, their applicability is restricted to specific messages; the effectiveness of the information provided may be partly driven or even conditioned by specific results of polls. Given the mixed findings and limitations, we expect that, when there is no intervention, individuals will not differ in their poll credibility perceptions when exposed to high- vs. low-quality polls (**H1**).

Preemptive interventions may help bypass these limitations. They have greater bandwidth—time and space—and thus can incorporate diverse strategies beyond those predicated on information deficit models (Bubela et al. 2009; Freiling, Krause, and Scheufele 2023). They are less confrontational and direct, which may avoid the illusory truth or backfire effects (Wood and Porter 2019; Udry and Barber 2024; Williams-Ceci, Macy, and Naaman 2024) (Supplementary Material section 1). Also, preemptive interventions are more effective than post hoc strategies in general (McDougall 2019). Thus, interventions may serve as a novel pathway for conditioning public opinion perceptions by indirectly and nonconfrontationally training the public to be more critical poll consumers. Yet, no research has investigated survey methodology training interventions, and only a few have examined preemptive interventions in public opinion research more broadly (e.g., Niederdeppe et al. 2014). We expect that individuals receiving interventions will confer greater credibility to high-quality polls (vs. low-quality polls) that they encounter afterward (**H2**).

## Improving Poll Literacy with Diverse Strategies

Given the bandwidth of preemptive interventions, we need a theory-driven design approach that exploits diverse strategies (cf. Amazeen and Krishna 2024). We focus on two such dimensions from behavioral intervention sciences, one from persuasion—informational vs. motivational components—and another from pedagogical theories—passive vs. active engagement with information. Next, we introduce each and explain how we synthesized them to develop three interventions.

### Informational vs. Motivational Components

A fundamental distinction among preemptive interventions is the underlying informational and motivational components (Kuru 2025a). Interventions targeting improved literacy/knowledge generally inform individuals about facts and techniques for critical engagement. Interventions such as literacy tips, training, and accuracy prompts are primarily informational and entail definitions, examples, and explanations of topics/issues (Vraga et al. 2015; Jones-Jang, Mortensen, and Liu 2021; Pennycook et al. 2024). The provision of factual information about survey methodology would be predominantly informational (e.g., Stadtmüller et al. 2022), although no work examined such information provision preemptively.

Interventions such as “psychological inoculation” or “(direct) warnings” (e.g., against misinformation) have distinctive motivational components (Cook, Lewandowsky, and Ecker 2017; van der Meer, Hameleers, and Ohme 2023). Inoculation involves providing fact- or technique-based training about the strategies used in targeted messages (Compton et al. 2021). It equips individuals with literacy and includes a “forewarning” component about persuasion attempts (Cook, Lewandowsky, and Ecker 2017; Maertens et al. 2025), referred to as “psychological immunity” against persuasion (Compton 2021). Forewarning is thus a motivational component that triggers vigilance. Warning people against threats (e.g., misleading polls), as studied in other contexts (van der Meer, Hameleers, and Ohme 2023; Hameleers 2023), could be another strategy by itself. These interventions are predominantly motivational, mobilizing cognitive resource/attention against threats. Motivational triggers may help in doubt regulation too, as in the processing of statistics (cf. Braithwaite and Sprague 2021). Hence, it is crucial to investigate informational and motivational components.

### Passive vs. (Inter)active Components

Second, we examine both passive and active components in interventions: information delivery (building declarative literacy) vs. exercising/engaging with information through practice (building declarative and procedural literacy). Passive literacy interventions could entail the delivery of news literacy tips/information sheets alone (Guess et al. 2020). Active literacy involves hands-on exercises like participating in interactive quizzes (Collier, Pillai, and Fazio 2023) or developing skills (Qian, Shen, and Zhang 2022). Interactivity may be an important asset; research shows that message interactivity increases message elaboration, leading to more learning and persuasion (Oh and Sundar 2015). Similarly, gamified inoculations are more active and effective (Basol, Roozenbeek, and Van der Linden 2020; Roozenbeek and van der Linden 2020).

For statistics, pedagogical research shows that exercise, formative assessments, and checking for understanding are crucial for learning, particularly in navigating *math anxiety* (Garfield and Chance 2000; Yang et al. 2021). Interactive learning involves trainees “doing something,” similar to taking a quiz (Carlson and Winquist 2011). Such scaffolding and interactivity can help individuals overcome math anxiety and strengthen associative memory networks among newly learned concepts (Pfau et al. 2005). At schools, highly interactive gamified statistics curricula are very effective (Smith 2017). In interventions targeting the public, multiple-choice quizzes improved memory for misinformation corrections but did not reduce misinformation belief (Collier, Pillai, and Fazio 2023). Interactive quizzes increased perceived knowledge and political interest (Chen et al. 2020). Thus, while quizzes alone may not be as effective, they may catalyze learning when combined with other informational and motivational components.

### Synthesizing Multi-Component Interventions

In the current study, balancing the theoretical, methodological, and practical considerations, we synthesized components from the two theoretical dimensions of information vs. motivation and passive vs. (inter)active to design and compare three different interventions: (1) passive literacy training (involving definitions, explanations, and examples about crucial methodological indicators such as sample representativeness, margin-of-error, etc.), (2) active literacy training (including an explanatory quiz, in addition to the passive literacy components), and (3) (active) inoculation (including a motivational trigger about poor-quality and misleading polls, in addition to active literacy components). Next, we elaborate on the rationale.

Theoretically, while recent research compared informational and motivational components, the passive vs. active distinction has yet to be examined in conjunction with informational and motivational components (Green et al. 2022; Kuru 2025a). Hence, despite the extensive research in separate lines of work in pedagogy (literacy) and persuasion (inoculation), it is crucial to understand whether information delivery (passive literacy) is enough by itself or whether there are added benefits coming from (1) interactivity (active literacy) as well as (2) interactivity and motivational triggers combined (“active inoculation” intervention). Inoculation research examined passive vs. active distinction extensively and finds that “active inoculation” is more effective (Basol, Roozenbeek, and van der Linden 2020; Roozenbeek and van der Linden 2020); however, only a few studies on literacy interventions did so (e.g., Qian et al. 2022). Whether active literacy interventions are as effective as active inoculation is thus a crucial question in this line of work that remains to be answered. We bring these lines of research into dialogue by comparing the three interventions.

On the methodological and practical front, our strategy follows the *increasing treatment dosage* regime (cf. Codding and Lane 2015). From passive literacy (first intervention) to inoculation (third intervention), the treatment intensity rises by adding components. This is crucial for intervention optimization on the practical front (Collins et al. 2024); for instance, if active literacy is effective and there is no added benefit from the additional component of motivational triggers, then motivational triggers can be ignored in implementation efforts that have to consider limited resources and participant retention concerns. While there is also a fourth combination possibility of “information + motivation” (i.e., “*passive* inoculation”) arising from both dimensions, we did not test it, given the increasing dosage strategy and external validity concerns (see Supplementary Material section 1). Further, extensive research on inoculation has shifted the focus to highly interactive interventions such as gamification, consistently finding them effective (Basol, Roozenbeek, and van der Linden 2020; Roozenbeek and van der Linden 2020), rendering “passive inoculation” less relevant. Thus, our approach prioritizes real-world impact and external validity instead of probing all hypothetical combinations (Collins et al. 2014; Johnson and Schoonenboom 2016; Leviton 2017), thereby balancing theory-based and practice-based considerations in intervention design (Glanz and Bishop 2010; IJzerman et al. 2020).

Hence, we directly compare the three interventions instead of the underlying dimensions that informed their components (absence vs. presence of motivational triggers and interactivity): given the increasing dosage regime and potential catalyzing benefits of each additional component as discussed above, we hypothesized that the inoculation intervention will be more effective than active literacy, and that active literacy will be more effective than passive literacy (**H3**).

## The Role of Preexisting Individual Differences

To understand the conditions under which interventions operate and to devise customized recommendations, we also investigated the moderating roles of individual differences.

We tested three distinct factors that tap individuals’ abilities and confidence in methodological and statistical concepts: education levels (demographic), science literacy (cognitive), and subjective numeracy (attitudinal/metacognitive). Those with higher education would be more attentive to methodological information (Kuru, Pasek, and Traugott 2020a). Given the methodological nature of interventions, the level of reasoning skills (i.e., scientific literacy) related to basic scientific methods will matter (Golumbic et al. 2022). Also, subjective numeracy has high predictive power for objective literacy measures (Fagerlin et al. 2007). Subjective numeracy taps confidence in statistics; we expect individuals to be more receptive to the interventions due to familiarity and confidence as manifested through higher subjective numeracy. Thus, trainees with higher (A) education levels, (B) scientific literacy, and (C) perceived numeracy will exhibit the effects of interventions more strongly (**H4**).

We also tested individuals’ own attitudes about issues covered in the polls to probe the role of motivational biases (e.g., Madson and Hillygus 2020) in the uptake of interventions. Given the lack of intervention research in this domain and the heterogeneity in the polarization of the two issues we studied (Supplementary Material section 1), we investigate these effects as research questions. We ask if polls that deliver unfavorable results will shape poll evaluations (**RQ1**), condition the effects of poll (methodological) quality on poll evaluations (**RQ2**), and interact with the effects of interventions (**RQ3**).

## Empirical Context

We conducted an experiment in December 2023 in Singapore, a multicultural country in Southeast Asia. We focused on two issues—public opinion on COVID-19 vaccine safety and policy views on AI regulation—which gives us leverage to test two important contemporary issues. These issues differ in (1) how polarizing they are and (2) their novelty. The COVID-19 vaccine has been a polarizing issue in Singapore over the past four years, more so than public views on AI regulation. For instance, a political party requested halting COVID-19 vaccines in 2024 (Channel News Asia 2024). Similarly, AI regulation is a critical issue due to the rise of chatbots like generative AI; there is extensive polling and media coverage around the time of data collection (Channel News Asia 2024). Planning for AI technologies is one of the government’s strategic focus areas, culminating in the National AI Strategy (Tham 2023). Hence, Singapore is a highly relevant place to observe the emergence and crystallization of public opinion on AI during the technological breakthrough.

## Methods

### Data

Ethics approval was received at the National University of Singapore (NUS-IRB-2023-781). The data and code are deposited in POQ’s Harvard Dataverse. The experiment was preregistered at *AsPredicted* with registration number #154000 (https://aspredicted.org/FD9_JCT); see Supplementary Material section 2 for the few minor deviations. We conducted a two-wave longitudinal online experiment with a nonprobability sample of individuals (legal adults in Singapore: aged between 21 and 80) with *Bilendi & respondi* (Appendix and Supplementary Material section 1).

Quotas were used to approximate demographic representation as much as possible. The power analysis is in Supplementary Material section 1. Wave 1 (W1) was collected between December 5 and 14, 2023, while W2 was collected between December 12 and 18, 2023. At least seven days passed after each respondent completed W1 before an invitation for W2 was sent. The sample size for W1 was N = 2,062, and for Wave 2 (W2), it was N = 1,278. The panel retention rate was 62 percent (Supplementary Material section 1).

While there is no true sampling response rate, as this is a panel sample (Baker et al. 2010), we computed a 62 percent response rate in the recruitment sample; we provided all information about survey attempts and participant removal/failure rates in the Appendix. For experimental work, nonprobability samples are widely used (Krupnikov, Nam, and Style 2021). Analyses are unweighted.

We removed eight speeders, three laggards, and 24 respondents who withdrew consent after the full debriefing. The effective sample size for W1 is N = 2,027, and the longitudinal sample (for the main analysis) is N = 1,076. Descriptives for the W1, the longitudinal sample, and those who dropped out are provided in Supplementary Material section 1. Attrition analysis is in Supplementary Material section 6. There was no differential attrition. The descriptive findings are generally comparable to the census information (Department of Statistics 2020); as usual with online panels, high-education respondents were overrepresented, but this did not distort the main results (Supplementary Material section 1).

### Protocol

At W1, participants first answered pretest measures on polls and issues. Respondents took either the control or one of the three interventions. Manipulation checks and a short partial debriefing followed (Supplementary Material section 1). W2 included poll exposure manipulations, outcome measures, manipulation checks, and the full debriefing.

### Manipulations/Measures

**Figure 1.**
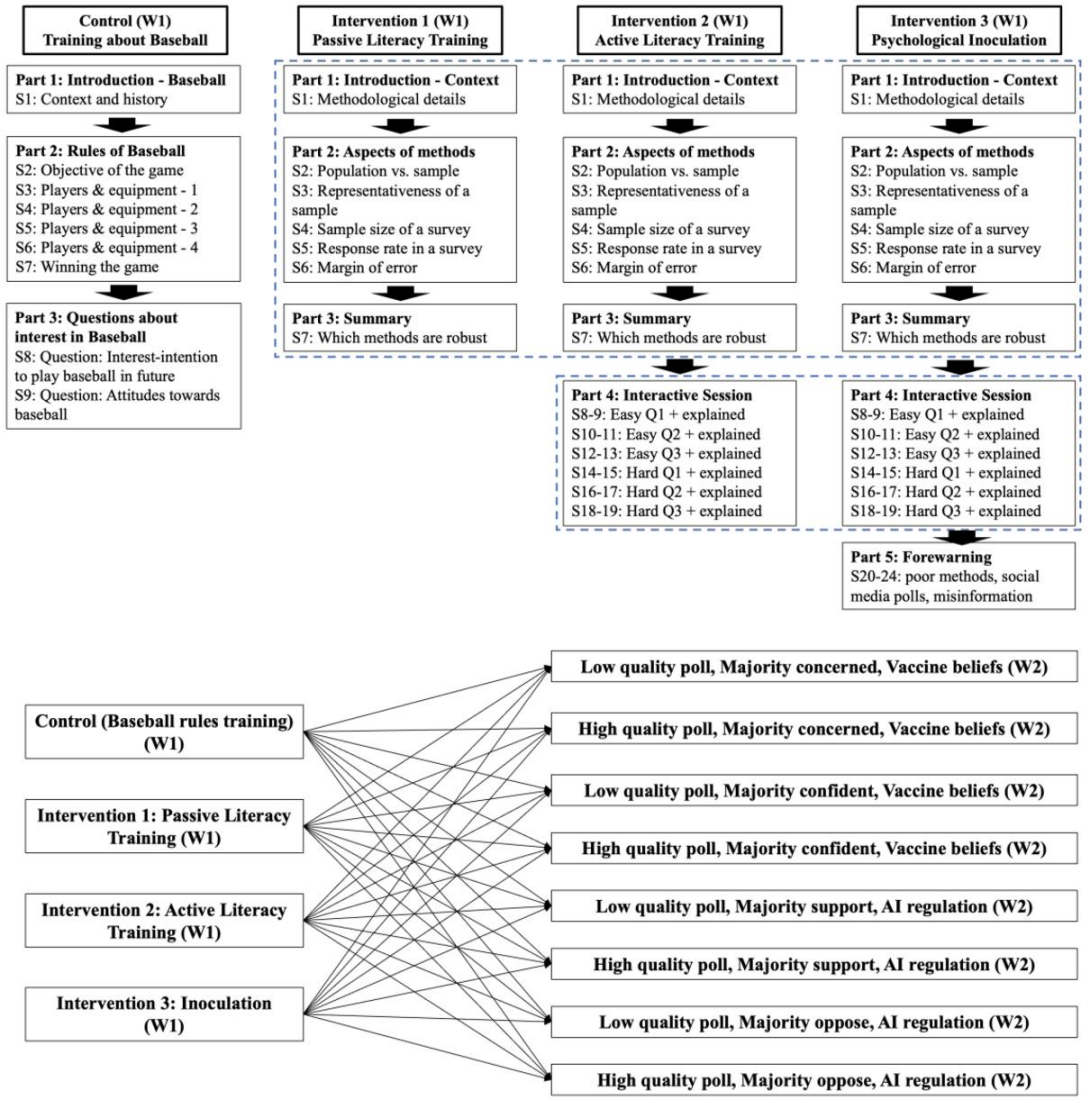
Wave 1 conditions (upper panel) and the Wave 1–Wave 2 crossing of conditions (bottom panel). “W1” denotes Wave 1, “S” denotes Slide, indicating slide numbers in training, and “Q” denotes question, indicating question numbers in training (“Easy” denotes the easier questions while “Hard” denotes the more challenging questions, “explained” indicates that the correct answer was explained to all participants after they provided an answer themselves).

This is a two-wave panel experiment where longitudinal conditions emerge from the full crossing of the conditions from both waves: W1 has one control and three intervention conditions: control, passive literacy training, active literacy training, and inoculation. W2 has eight conditions with the full factorial crossing of three manipulations: the methodological quality of the poll (poor vs. robust), poll result directionality (majority pro vs. anti), and the issue (COVID-19 vaccine perceptions and AI regulation policy views). An example for W2 condition is thus “a low-quality poll showing majority support for AI regulation.” Respondents were assigned to one of the 32 longitudinal conditions (figure 1; 4 in W1 by 8 in W2).

At W1, the control involves training about baseball. Passive literacy intervention involves introducing methodological details of polls, covering five key components (e.g., sampling type), and ending with a summary slide. Active literacy has an additional element: an interactive quiz session. The quiz includes three relatively less challenging (two response options and fact based) and three challenging questions (four response options and scenario based). The correct answers are explained to all participants after answering each question. Finally, inoculation intervention additionally has the forewarning component, which warns respondents about low-quality, misleading, and misinformation polls. Thus, the three interventions shared common (information) and distinct (quiz and forewarning) components, testing the added benefits of (1) active (quiz) and (2) active (quiz) and motivational (warning) components combined.

Comparing the three interventions against each other and a true control condition, we thereby effectively treat all W1 conditions as a “multiple-arm comparative experiment” (MACE; “a k-arm experiment involving k-1 different forms of treatment and a control”) (Collins et al. 2014). This nonfactorial approach is appropriate for the research purpose of (1) determining whether interventions are effective (against control) and (2) evaluating which of the three interventions is the most effective (in comparison to each other and the control).

Importantly, in producing the interventions, we also used insights and examples (e.g., scaffolding approach, soup analogy for sampling, some quiz items, see Supplementary Material section 1) from the education programs that we reviewed (see the Discussion section). These content-related and technical insights provide greater ecological validity to the interventions (Johnson and Schoonenboom 2016).

At W2, methodological quality is the key manipulation. High-quality polls are mostly probability-based samples with larger sample sizes and response rates, and they provide the most accurate portraits of public opinion with a lower margin of error (Baker et al. 2010). These stand in contrast to nonprobability polls, lower sample sizes, and response rates (Pasek 2015; Mercer, Kennedy, and Keeter 2024). We characterize our conceptualization and operationalization of quality as an omnibus approach (as in Kuru et al. 2017; Kuru, Pasek, and Traugott 2020b) instead of a methodological aspect-specific approach (as in Stadtmüller et al. 2022). The omnibus approach assumes that a general methodological quality evaluation can be made by taking into account methodological aspects collectively. We discuss three critical reasons for this choice in Supplementary Material section 1. Manipulations worked (Supplementary Material section 4). Details about the result and issue manipulation, further details on the methodological quality manipulation, and all other measures are in table 1 and Supplementary Material section 1.

**Table 1.** Details of measures.

Names	Question wording	Operationalization and other details
Wave 2 manipulations		
Methodological quality	NA	Experimental predictor, based on W2 manipulations Contrasts polls with high methodological quality (coded 1, N = 534) against those with low quality (coded 0, N = 542) Low quality: “The survey was conducted with a convenience sample of online news consumers of the major digital news platforms in October 2023, and the response rate was 7%. The sample size of the survey was 289 adults (21 years old and older). The margin of error for this result is +/− 7 percentage points. The participants were recruited through invitations in the websites and social media accounts of digital news channels and answered the questions through an online form.” High quality: “The survey was conducted on a nationally representative probability-based sample in October 2023, and the response rate was 70%. The sample size of the survey was 1,537 adults (21 years old and older). The margin of error for this result is +/− 2 percentage points. The participants were recruited through address-based sampling and were interviewed either face to face or through telephones with human interviewers.” See Supplementary Material section 1 for justification for these numbers on sample size, response rate, etc., and the full wordings for low vs. high methodological quality polls.
COVID-19 vaccine vs. AI issue	NA	Experimental predictor based on W2 manipulations Contrasts polls on AI regulation (coded 0, N = 538) to those on COVID-19 vaccines (coded 1, N = 538)
Poll result	NA	Experimental predictor based on W2 manipulations Contrasts poll showing majority concern about vaccines (N = 269) / majority opposition to AI regulation (N = 266) (coded 1) against those showing majority confidence about vaccines (N = 269) / majority support for AI regulation (N = 272) (coded 0)
Outcome and predictor variables		
Perceived credibility of polls	“How [reliable / accurate / valid / credible / scientific] do you find the survey finding you just read?” There are five item-specific response options ranging from “Not [reliable / accurate / valid / credible / scientific] at all” to “Extremely [reliable / accurate / valid / credible / scientific].”	Primary outcome variable with items adopted from (Kuru et al. 2017; Stadtmüller et al. 2022) Measured at W2 only as outcome variable Five items’ reliability was high (α = 0.95). The items were averaged into an index that runs from 0 to 1, where higher scores represent more perceived credibility (Mean = 0.42, sd = 0.20, Median = 0.45). We asked the last item, “scientific,” on a separate page after the first four items were measured. During design, we were slightly concerned this item might cue respondents to think about methods more directly and hence reduce external validity. Hence, to prevent such a possibility, we asked this question on a separate, second page. Excluding this item did not change the reliability (α = 0.95), distribution (Mean = 0.43, sd = 0.21, Median = 0.50), or the main results.
Education	What is the highest level of school you have completed or the highest degree you have received? “Primary school or less,” “Some high school but no diploma,” “High school graduate,” “Some college but no degree,” “Polytechnic,” “Bachelor’s degree in college (4-year),” “Master’s degree,” “Doctoral degree,” “Professional degree.”	Measured at W1 pretest as a moderator The single-item question has 9 response options. Four different fundamental levels are created by recoding these 9 groups: high school education or less (N = 226, 11 percent), some college but no degree (N = 549, 27 percent), college or equivalent degree holders (N = 1,030, 51 percent), and some type of graduate or professional degree (N = 222, 11 percent). > Ranging from 0 to 1, higher scores represent greater education (Mean = 0.54, sd = 0.27, Median = 0.67).
Scientific literacy	“Alex is overweight and wants to find a research-proven method to lose weight. In the next page, we will present 8 short questions about different aspects of Alex’s search for an effective diet. For each question please answer: True or False. Please provide your best guess if you are not sure.” Item example: “As part of Alex’s attempts to lose weight, he decides to stop eating in between meals and to run on the beach. A week later he finds out he lost 5 kg. Alex can determine with certainty the cause of his weight loss. True or False?” The response options were composed of true or false, and there was only one correct answer.	Measured at W1 pretest as a moderator Eight items are adopted from the Everyday Scientific Reasoning scale (Golumbic et al. 2022). Items were updated to remove location names and double negatives in the original scale. Full list of items are in Supplementary Material section 1; all items had two response options. Reliability is not relevant (α = 0.60), as is common with objective knowledge / performance measures. Being able to answer one item does not guarantee similar performance in a harder item, and items do not measure an underlying dimension of a psychological construct. The items were averaged into an index ranging from 0 to 1, where higher scores indicate stronger scientific literacy (Mean = 0.45, sd = 0.25, Median = 0.50; N = 68 answered all items correctly, N = 116 scored zero).
Subjective numeracy	Example item: “How good are you at figuring out how much a shirt will cost if it is 25 percent off?” There were five options ranging from “Not at all good” to “Extremely good.”	Measured at W1 pretest as a moderator Eight items from an established scale have been used with slight improvement edits in response options wording (Fagerlin et al. 2007). Full list of items are in Supplementary Material section 1; all items had five response options. Reliability was high (α = 0.88); the items were averaged into an index ranging from 0 to 1, where higher scores indicate stronger subjective numeracy (Mean = 0.55, sd = 0.19, Median = 0.56).
Unfavorable poll	For each issue (vaccine vs. AI), a separate variable is constructed to index “unfavorable poll” based on the directionality of poll findings (Poll Result) and preexisting (measured as W1 pretest) issue positions. Preexisting view items: “How risky do you think [the side effects of COVID-19 vaccines are / the use of artificial intelligence (AI) technologies in settings such as work, school, and healthcare is]?” with 5 response options from “Not risky at all” (0) to “Extremely risky” (1). Refer to row 3 in this table for details on poll result variable. We also created a pooled version of this variable by combining vaccine and AI issues (as a predictor in alternative models). We obtained the same results when we used an alternative coding for the AI issue that used pretest policy views instead.	Constructed by combining two variables, one from W1 (issue position) and one from W2 (poll result) and used as a moderator We subtracted the distribution of preexisting issue positions from the experimental poll result factor based on directionality. Resulting continuous variable, ranging from 0 to 1, shows how unfavorable the poll is for each participant—either in vaccine (Mean = 0.49, sd = 0.28, Median = 0.50) or AI issue (Mean = 0.48, sd = 0.25, Median = 0.50), with 0 indicating preexisting views strongly in agreement/favor of the position shown in the poll result (respondent’s position matches that of the majority’s preference). The continuous scale shows both whether a viewed poll was unfavorable as well as the strength of this valence.
Interest in polls	“How interested are you in reading survey / poll findings?” with five response options ranging from “Not at all interested” to “Extremely interested.” “How frequently do you read survey / poll findings?” with five response options ranging from “Never” to “Very frequently.” “How much attention do you give to survey / poll findings that are mentioned in news articles or in other media?” with five response options ranging from “No attention at all” to “A lot of attention.”	Measured at W1 pretest as an important theoretical control variable. Three items were adopted from similar measures (Kuru et al. 2017). Reliability was high (α = 0.88); the items were averaged into an index ranging from 0 to 1, where higher scores indicate greater interest in polls (Mean = 0.55, sd = 0.20, Median = 0.50).

*Note*: For other measures, such as age, sex, race, and income, as well as other additional robustness check control variables or outcomes (i.e., evaluations of polls in general), please see Supplementary Material section 1. Both vaccines and AI-related pretest measures were asked of all respondents, while respondents answered only vaccine or AI questions in the posttest, depending on the topic of the poll they were shown. To avoid demand effects, participants were not asked about the second issue (they only answered posttest questions about the issue covered in the poll they were assigned). The only notable missing response case was observed for income. The missing scores for N = 51 respondents who chose “Do not want to respond” were imputed with the median income value 5 (see Supplementary Material section 1). Given that this is a peripheral control variable, a simple median imputation was chosen instead of the multiple imputation technique. Other very minor missing cases were treated with listwise deletion in the analytical models.

### Analytical Strategy

Balance tests documented the equivalence of conditions across most variables, and cell Ns were also balanced (no differential attrition from W1; Supplementary Material section 3 and Supplementary Material section 6). Longitudinal interference tests were also conducted (Supplementary Material section 6). At W1, balance tests showed that only science literacy was slightly imbalanced, but this is a moderator and not part of the main models. Hence, it is not included as a control. At W2, balance tests showed small but significant differences for respondent age, Chinese and Indian respondents, and pretest poll interest scores across W2 conditions and W1 dropouts. Hence, we controlled for these four variables: age, Chinese, Indian, and poll interest of respondents. We also provided supplementary results (1) with baseline models without any controls and (2) including a further set of controls aside from balance check related variables, showing the same findings (Supplementary Material section 5). We conducted a pooled analysis by combining the vaccine and AI issues to predict poll credibility (additionally, the issue type was controlled for), and two issues were separately tested as well, showing the same results. OLS regressions were used, given the continuous outcomes and two-way interactions. R software (open source) was used for analyses.

### ***Main Effects Models (*** ***Tables 2*** ***and***  ***3******)***

First, we tested the effect of poll quality with and without accounting for experimental conditions to answer H1. We employed three different analytical approaches for the main effects of interventions due to the complexity of the experiment, to avoid three-way interactions (and reduced statistical power), to compare overall intervention effectiveness, and to triangulate the results. First, the original (preregistered) Model 1 tests two-way (2 by 4) interactions (*methodological quality X intervention*). For Model 2, we created eight (2 X 4) groups to avoid the two-way interaction; and we used “the control (W1) participants exposed to low-quality (W2) polls” as the reference category. We then elicited the 28 Tukey-adjusted pairwise differences between any of these groups; Models 1 and 2 gave the same results (table 3), answering H2 and H3, yet they do not provide a holistic comparison of “the effectiveness of the interventions” (comparing across low- vs. high-quality polls). As suggested by a reviewer, we split the dataset based on W1 conditions and ran the models (poll quality was the main predictor, Model 3 in table 2). This allowed for comparing the overall effectiveness of interventions. We then implemented nonparametric-bootstrapping (N = 5,000) and compared the CI and *p*-values for the pairwise difference in “Low- vs. High-Quality Poll” (row 1, table 2) across the four models.

**Table 2.** Predicting perceived credibility of polls by interventions and poll quality conditions.

			Analytical strategy 1	Analytical strategy 2	Analytical strategy 3			
		Model 0: Baseline model	Model 1: Two-way (4 by 2) interactions	Model 2: Eight groups	Model 3A: Control participants	Model 3B: Passive literacy participants	Model 3C: Active literacy participants	Model 3D: Inoculation participants
Row #								
(1)	Low- vs. high-quality poll (Wave 2)	0.061 (0.012) *p = *0.000	0.021 (0.023) *p = *0.369		0.022 (0.024) *p = *0.354	0.052 (0.024) *p = *0.031	0.050 (0.023) *p = *0.033	0.125 (0.024) *p = *0.00000
(2)	Passive literacy intervention (Wave 1)		−0.010 (0.023) *p = *0.659					
(3)	Active literacy intervention (Wave 1)		0.009 (0.024) *p = *0.704					
(4)	Inoculation intervention (Wave 1)		−0.050 (0.024) *p = *0.035					
(5)	Passive literacy intervention x low- vs. high-quality poll		0.031 (0.033) *p = *0.344					
(6)	Active literacy intervention x low- vs. high-quality poll		0.026 (0.033) *p = *0.437					
(7)	Inoculation intervention x low- vs. high-quality poll		0.102 (0.033) *p = *0.003					
(8)	Control participants exposed to high-quality poll			0.021 (0.023) *p = *0.369				
(9)	Passive intervention participants exposed to low-quality poll			−0.010 (0.023) *p = *0.659				
(10)	Passive literacy intervention participants exposed to high-quality poll			0.042 (0.024) *p = *0.084				
(11)	Active literacy intervention participants exposed to low-quality poll			0.009 (0.024) *p = *0.704				
(12)	Active literacy intervention participants exposed to high-quality poll			0.056 (0.024) *p = *0.019				
(13)	Inoculation intervention participants exposed to low-quality poll			−0.050 (0.024) *p = *0.035				
(14)	Inoculation Intervention Participants Exposed to High Quality Poll			0.072 (0.024) *p = *0.003				
	Constant	0.300 (0.028) *p = *0.000	0.310 (0.031) *p = *0.000	0.310 (0.031) *p = *0.000	0.256 (0.060) *p = *0.00003	0.279 (0.054) *p = *0.00000	0.345 (0.051) *p = *0.000	0.307 (0.058) *p = *0.00000
	*N*	1,076	1,076	1,076	273	269	263	271
	*R* ^2^ / Adjusted *R*^2^	0.140 / 0.135	0.150 / 0.141	0.150 / 0.141	0.071 / 0.050	0.165 / 0.146	0.188 / 0.169	0.200 / 0.182
	*F* statistic	29.02 (df = 6; 1,069), *p = *0.000	15.653 (df = 12; 1,063), *p = *0.000	15.653 (df = 12; 1,063), *p = *0.000	3.385 (df = 6; 266), *p = *0.003	8.610 (df = 6; 262), *p = *0.000	9.880 (df = 6; 256), *p = *0.000	11.021 (df = 6; 264), *p = *0.000

*Note*: The models are based on OLS regressions, and the values in the numbered (1 to 14) rows show unstandardized coefficients, standard errors (in parentheses), and the associated significance (*p*) values (two tailed). Model 1 is based on a two-way interaction of two manipulations as predictors, while Model 2 is based on a dummy variable testing of eight resultant conditions as predictors. The same results are obtained from the data from three different modeling strategies. Reference category for Model 1: Control (no intervention) in Wave 1 (W1). The reference category for Model 2: Control participants exposed to low methodological quality polls. Model 3 provides subsetted analysis based on W1 arms (control and intervention groups) to allow for direct significance testing for the differences in the effectiveness of interventions through nonparametric bootstrapping. The reference category for Model 3 (3A to 3D) is low methodological quality poll conditions. All results control for pretest imbalance variables (age, Chinese, Indian, interest in polls) and the issue type (COVID-19 vaccine vs. AI risk); full models showing coefficients for control variables are in Supplementary Material section 5. The same substantive results are obtained in models without any control variable and models that control for additional sets of variables, including exposure to unfavorable poll results (Supplementary Material section 5).

**Table 3.** Pairwise linear tests (Tukey adjusted *p*-values) predicting perceived credibility of polls by interventions and poll quality conditions.

Row #	Estimated mean differences between pairs of conditions	Estimate	Std. Error	*t*-value	*Pr(> | t|)*
(1)	Control seen high-quality – control seen low-quality	0.021	0.023	0.901	0.986
(2)	Passive seen low-quality – control seen low-quality	−0.010	0.023	−0.441	1.000
(3)	Passive seen high-quality – control seen low-quality	0.042	0.024	1.730	0.668
(4)	Active seen low-quality – control seen low-quality	0.009	0.024	0.381	1.000
(5)	Active seen high-quality – control seen low-quality	0.056	0.024	2.352	0.267
(6)	Inoculation seen low-quality – control seen low-quality	−0.050	0.024	−2.119	0.403
(7)	Inoculation seen high-quality – control seen low-quality	0.072	0.024	3.050	0.048
(8)	Passive seen low-quality – control seen high-quality	−0.031	0.023	−1.388	0.863
(9)	Passive seen high-quality – control seen high-quality	0.021	0.024	0.891	0.987
(10)	Active seen low-quality – control seen high-quality	−0.012	0.023	−0.510	1.000
(11)	Active seen high-quality – control seen high-quality	0.035	0.023	1.512	0.801
(12)	Inoculation seen low-quality – control seen high-quality	−0.071	0.023	−3.089	0.043
(13)	Inoculation seen high-quality – control seen high-quality	0.051	0.023	2.222	0.339
(14)	Passive seen high-quality – passive seen low-quality	0.053	0.024	2.221	0.339
(15)	Active seen low-quality – passive seen low-quality	0.019	0.023	0.836	0.991
(16)	Active seen high-quality – passive seen low-quality	0.066	0.023	2.878	0.078
(17)	Inoculation seen low-quality – passive seen low-quality	−0.040	0.023	−1.751	0.653
(18)	Inoculation seen high-quality – passive seen low-quality	0.082	0.023	3.608	0.008
(19)	Active seen low-quality – passive seen high-quality	−0.033	0.024	−1.357	0.876
(20)	Active seen high-quality – passive seen high-quality	0.014	0.024	0.568	0.999
(21)	Inoculation seen low-quality – passive seen high-quality	−0.093	0.024	−3.818	0.004
(22)	Inoculation seen high-quality – passive seen high-quality	0.030	0.024	1.239	0.920
(23)	Active seen high-quality – active seen low-quality	0.047	0.024	1.973	0.500
(24)	Inoculation seen low-quality – active seen low-quality	−0.060	0.024	−2.505	0.194
(25)	Inoculation seen high-quality – active seen low-quality	0.063	0.024	2.664	0.135
(26)	Inoculation seen low-quality – active seen high-quality	−0.107	0.024	−4.504	0.0002
(27)	Inoculation seen high-quality – active seen high-quality	0.016	0.023	0.685	0.997
(28)	Inoculation seen high-quality – inoculation seen low-quality	0.123	0.023	5.221	0.00000

*Note*: The models are based on pairwise difference tests deriving from the OLS regressions and present the estimated marginal mean differences, standard errors, and the associated t and significance (*p*) values (two tailed). Example of reading the mean differences: “Control seen high-quality – control seen low-quality” indicates the subtraction of “the estimated mean of control (W1) participants who were exposed to low-quality polls (W2)” from “the estimated mean of control (W1) participants who were exposed to high-quality polls (W2)”. These results are the same across both modeling strategies reported in the main results in table 2. The effects observed in Model 2, row 12, and in Model 3B and 3C in row 1 (all in table 2) for passive and active literacy interventions dissipated in pairwise tests due to *p*-value adjustments in pairwise comparisons. Only inoculation effects persist and do so robustly.

### ***Moderator Models* (** ***Table 4*** **)**

To avoid reduced statistical power and higher-order interactions, and given that we did not hypothesize any difference among interventions when it comes to their moderation by individual differences, we combined all interventions (against control) and then crossed this variable with poll-quality manipulation. This pooled strategy (not preregistered), building on the validated strategy of Model 2 (table 2), created four groups: control seen low-quality poll, control seen high-quality poll, intervention groups seen low-quality poll, and intervention groups seen high-quality poll. This four-group variable is interacted with individual difference moderators in two-way interactions. These models answer H4 and RQ1-3. The preregistered models testing interventions separately for moderation hypotheses—albeit with reduced power and similar results—are in Supplementary Material section 5.

**Table 4.** Moderation of experimental effects by preexisting individual differences in predicting perceived credibility of polls.

Row #		Model 0: Baseline	Model 1: Education	Model 2: Science literacy	Model 3: Subjective numeracy	Model 4A: Unfavorable poll (Vaccine)	Model 4B: Unfavorable poll (AI)	Model 4C: Unfavorable poll (pooled: Vaccine +AI)
(1)	Control seen high-quality polls	0.021 (0.023) *p = *0.369	0.053 (0 .051) *p = *0.307	-0.134 (0.049) *p = *0.007	0.041 (0.073) *p = *0.579	0.019 (0.061) *p = *0.760	−0.099 (0.072) *p = *0.173	−0.030 (0.046) *p = *0.525
(2)	Interventions seen low-quality polls	−0.017 (0.019) *p = *0.381	0.061 (0.041) *p = *0.135	−0.045 (0.041) *p = *0.269	0.062 (0.060) *p = *0.299	−0.041 (0.052) *p = *0.435	−0.112 (0.062) *p = *0.070	−0.067 (0.039) *p = *0.088
(3)	Interventions seen high-quality polls	0.057 (0.020) *p = *0.004	0.051 (0.041) *p = *0.211	−0.029 (0.041) *p = *0.481	0.038 (0.060) *p = *0.527	0.051 (0.052) *p = *0.330	−0.007 (0.064) *p = *0.909	0.030 (0.040) *p = *0.450
(4)	Individual difference*		0.014 (0.060) *p = *0.819	−0.306 (0.066) *p = *0.00001	0.050 (0.088) *p = *0.567	−0.128 (0.079) *p = *0.110	−0.183 (0.100) *p = *0.067	−0.142 (0.062) *p = *0.022
(5)	Control seen high-quality polls x individual difference		−0.056 (0.084) *p = *0.507	0.316 (0.089) *p = *0.0005	−0.035 (0.123) *p = *0.779	0.035 (0.112) *p = *0.755	0.209 (0.134) *p = *0.120	0.105 (0.185) *p = *0.219
(6)	Interventions seen low-quality polls x individual difference		−0.142 (0.069) *p = *0.040	0.031 (0.075) *p = *0.679	−0.139 (0.100) *p = *0.164	0.094 (0.093) *p = *0.316	0.146 (0.113) *p = *0.195	0.106 (0.071) *p = *0.137
(7)	Interventions seen high-quality polls x individual difference		0.012 (0.069) *p = *0.868	0.160 (0.076) *p = *0.037	0.037 (0.101) *p = *0.712	0.049 (0.094) *p = *0.605	0.098 (0.115) *p = *0.397	0.060 (0.073) *p = *0.407
	Constant	0.313 (0.031) *p = *0.000	0.297 (0.042) *p = *0.000	0.462 (0.045) *p = *0.000	0.283 (0.058) *p = *0.00001	0.338 (0.056) *p = *0.000	0.424 (0.067) *p = *0.000	0.380 (0.043) *p = *0.000
	*N*	1,076	1,076	1,076	1,076	538	538	1,076
	*R* ^2^ / Adjusted *R*^2^	0.144 / 0.137	0.155 / 0.146	0.211 / 0.203	0.149 / 0.139	0.163 / 0.145	0.149 / 0.131	0.153 / 0.143
	*F* statistic	22.376 (df = 8; 1,067), *p = *0.000	16.304 (df = 12; 1,063), *p = *0.000	23.753 (df = 12; 1,063), *p = *0.000	15.480 (df = 12; 1,063), *p = *0.000	9.292 (df = 11; 526), *p = *0.000	8.386 (df = 11; 526), *p = *0.000	15.976 (df = 12; 1,063), *p = *0.000

*Note*: The models are based on OLS regressions and the values in the numbered (1 to 7) rows show unstandardized coefficients, standard errors (in parentheses), and the associated significance (*p*) values (two tailed). *Individual difference denotes individual difference moderators, each named in the respective model titles at the top column. For example, in Model 1, the individual difference is Education (participants’ education levels). The reference category for experimental predictors is “Control (participants) Seen Low-Quality polls.” Three intervention groups are pooled and compared against control in these models. Science Lit. denotes science literacy; Subj. Num. denotes subjective numeracy. In this analysis, note that three interventions are pooled. Results control for pretest imbalance variables (age, Chinese, Indian, Interest in Polls) and issue type (COVID-19 vaccine vs. AI risk). Issue type is not controlled for in Models 4 and 5, as these are already based on subsetted (issue-based) data. Testing Model 4A and 4B conditional on poll result directionality (e.g., majority pro- vs. anti-) did not change the results (this test is irrelevant for Model 4C, as it is pooled across both issues).

## Results

### Interventions’ Main Effects

First, we tested whether methodological quality predicted poll evaluations among all participants without accounting for intervention effects. Across all conditions, respondents evaluated high-quality polls as more credible than low-quality polls (table 2, Model 0, row 1). However, this effect was entirely driven by the three training interventions. Among the control group, the perceived credibility of high- vs. low-quality polls did not differ (table 2, Model 3A, row 1); they differed only among intervention conditions (table 2, Models 3B, C, and D; also table 4, Model 0). Thus, H1 and H2 were supported.

Next, we compared the three interventions. Interventions interacted with the methodological quality of polls, showing their intended effectiveness, and this was driven by inoculation intervention (table 2, M1, row 7; Model 2, rows 13 and 14; and Model 3D). Based on Models 1 and 2 in table 2 (producing the same results), we conducted post hoc pairwise comparison tests for all 28 possible pairwise comparisons. These pairwise differences (Tukey’s adjusted *p*-values) are in table 3. There was a significant difference between the credibility of low- and high-quality polls in the inoculation group (table 3, row 28). In terms of effect size, this corresponds to a more than 12 percent change within the range of the perceived credibility index. Given the credibility index has five questions with five response options each, on average, this effect size can be viewed as a one-unit movement in response options of at least two of the items, which we characterize as a small effect size. It is a remarkable longitudinal (delayed/already decayed) effect given that it had not been cultivated immediately after the interventions.

Inoculation conditions exposed to low- vs. high-quality polls have a few other significant differences, particularly from the control and passive literacy conditions (e.g., table 3, rows 7 and 12). This pattern was also observed between low- and high-quality polls in passive and active literacy intervention groups (e.g., passive literacy seen in low- vs. high-quality polls), but it was not persistent based on adjusted *p*-values (which should be prioritized as a more conservative test over the effects in table 2, Model 3B and 3C, row 1).

**Figure 2.**
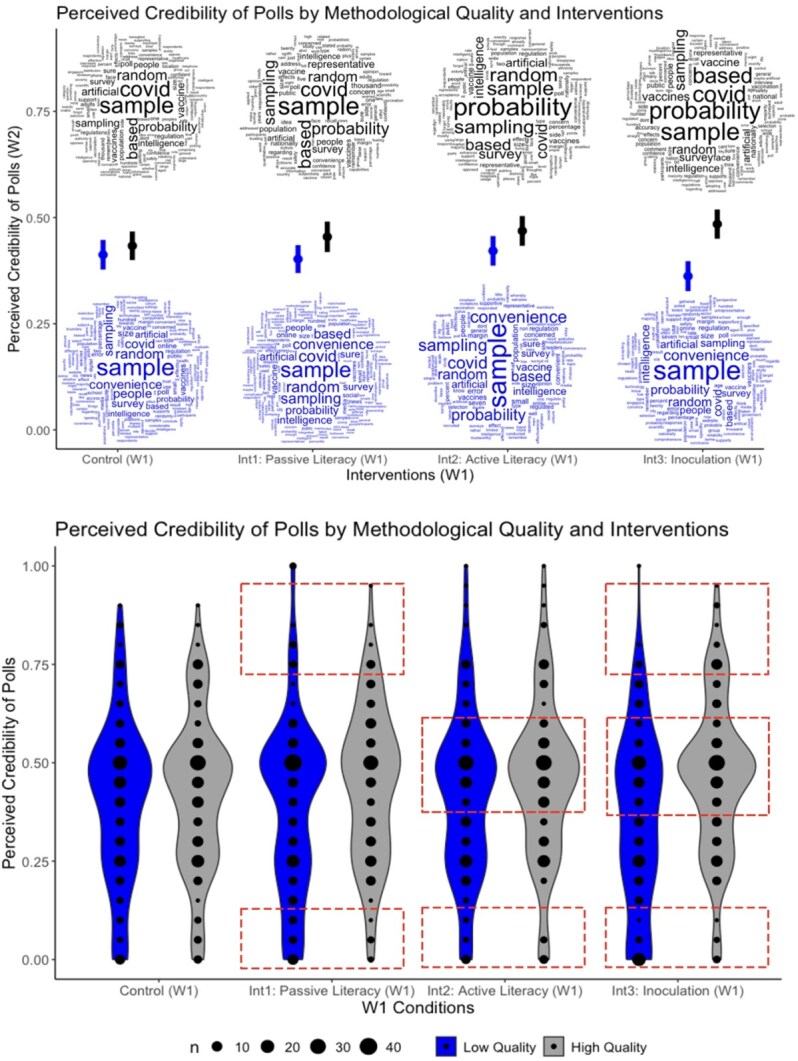
Main effects of interventions on poll perceptions. The top panel shows the marginal means of conditions in perceived poll credibility and word clouds produced from open-ended responses across conditions. The bottom panel shows the distribution of predicted values, with red highlights indicating important distributional differences between the conditions. See Supplementary Material section 4 for enlarged word clouds.

We visualize these effects in figure 2. In the top panel, estimated marginal means are presented where the effects of interventions are visible, and most prominently, in the inoculation intervention, where low-quality polls were evaluated more negatively than high-quality polls. The word clouds visualizing the relative frequency of key terms mentioned by respondents in response to the open-ended question about polls also illustrate that, overall, interventions worked. The relative frequency of mentioning “probability sample” (in high-quality poll conditions; top/black clouds) and “convenience sample” (in low-quality poll conditions; bottom/blue clouds) was much larger in the inoculation group than the other W1 conditions, especially the control group. Finally, in the bottom panel, we present the distributions, further illustrating the differences among W1 groups. Rectangles indicate where distributional differences are most evident: inoculation shifted the evaluations throughout the distribution, while the shifts are minor in the other interventions.

We also compared the overall effectiveness of three interventions. Nonparametric bootstrapping (table 2, Model 3, row 1) showed that inoculation’s effectiveness in creating credibility gap between low- vs. high-quality polls significantly differed from all other W1 conditions: passive literacy intervention ([0.005, 0.138], *p *= 0.034), active literacy intervention ([0.010, 0.141], *p *= 0.025), and the control condition ([0.037, 0.168], *p *= 0.003). On the other hand, passive and active literacy did not significantly differ from the control ([−0.035, 0.096], *p *= 0.358 and [−0.040, 0.090], *p *= 0.400, respectively) or from each other ([−0.068, 0.060], *p *= 0.90).

The results mostly support H3, as the inoculation intervention had a robust effect (consistently shown through three models and adjusted pairwise tests). Other interventions were not effective despite exhibiting the same trend.

### Moderation by Individual Differences

Participants’ education (H4A) and scientific literacy (H4B) levels moderated the intervention effects, while subjective numeracy (H4C) and the favorability of poll results to preexisting views (RQ3) did not.

**Figure 3.**
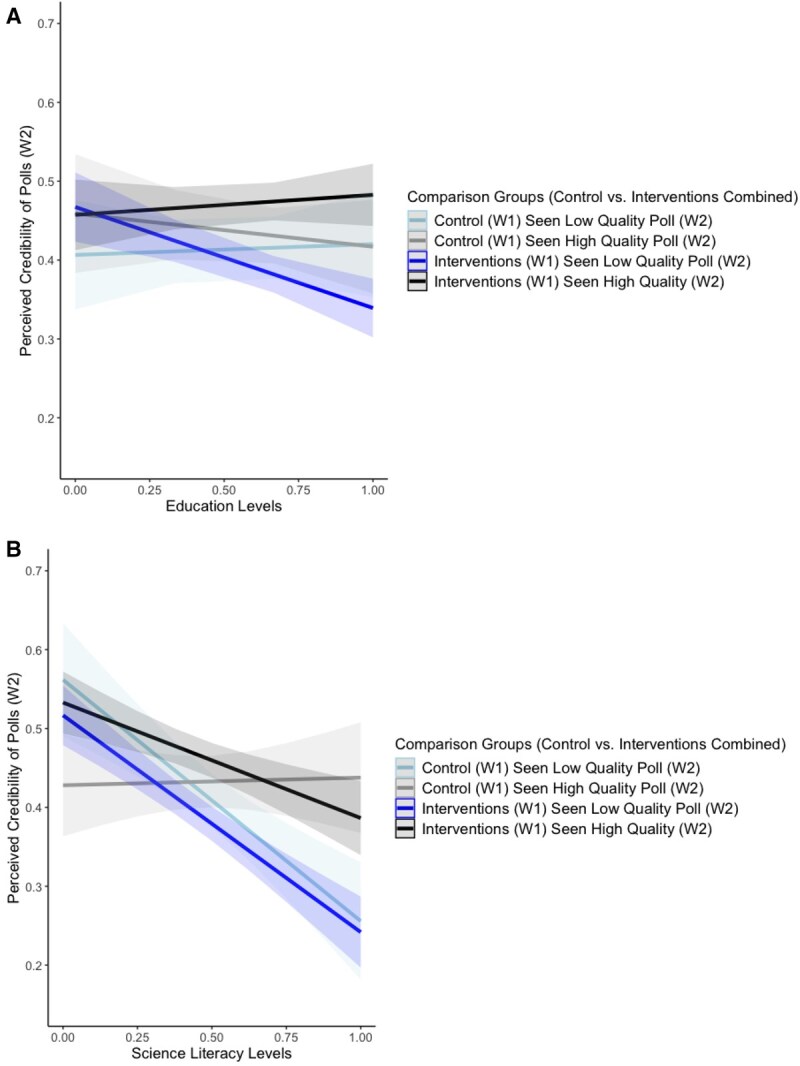
The effects of interventions on poll perceptions moderated by education and scientific literacy. The top panel (figure 3A) shows the interaction effects for education (based on Model 1 in table 4), while the bottom panel (figure 3B) shows the interaction effects for science literacy (based on Model 2 in table 4). Example reading of the comparison groups in the legend: “Control (W1) Seen Low Quality Poll (W2)” indicates the group that consists of W1 control participants who were exposed to a low-quality poll at W2.

Education moderated the interventions’ effects (table 4, Model 1, row 6). As seen in the slope of the dark blue line in figure 3A, compared to low-education respondents, high-education respondents discredited low-quality polls notably, demonstrating the effect of interventions for this group. There was no such trend among control participants. Education levels did not alter the assessments of high-quality polls (flatter slopes of gray and black lines). When controlling for all individual difference moderators simultaneously in a more conservative test, education moderation was not significant, mainly due to the effect of science literacy.

Scientific literacy moderated the effects of methodological quality, including the control and intervention groups. There were two significant interactions between comparison groups and science literacy (table 4, Model 1, rows 5 and 7). The pattern of “increasing differences in assessments of low- vs. high-quality polls as science literacy increases” (figure 3B) is visible for both the interventions and the control group. Science literacy’s significant conditioning effect persists when all other moderators are simultaneously controlled for.

Respondents significantly discredited polls that conflicted with their preexisting views for vaccines (Supplementary Material section 5, b = −0.072, se = 0.030, *p *= 0.016) but not for the AI issue (b = −0.052, se = 0.034, *p *= 0.127; RQ1). This bias coefficient was significant when issues were analyzed together (pooled, b = −0.063, se = 0.022, *p *= 0.005). However, for both issues and the pooled version, the favorability of polls (1) did not interact with the poll quality manipulation (Supplementary Material section 5; RQ2) and (2) did not moderate the effects of interventions (Models 4, table 4; RQ3).

#### Additional Analysis

We asked respondents their views about polls in general, both at the W1 pretest and at the W2 posttest. Polling evaluations slightly shifted to be more negative among all returning participants (including those in the W1 control). This decline was not moderated by interventions, and pretest general poll evaluations did not moderate the intervention effects (Supplementary Material section 7).

## Discussion

Polls represent a frontier between social science and the general public that social research is meant to serve, constituting a “public good” (Smidt 2024). As the communication of public opinion progresses toward a more participatory era (Lupia 2023), training the public to cultivate their critical consumption of public opinion statistics is of paramount significance (Wallman 1993; Jamieson et al. 2023). This is especially important given the polls’ role in perceptions of public opinion, misinformation, trust in media, and polarization (Kuru, Pasek, and Traugott 2020a; Silber et al. 2022; Graham, Hillygus, and Trexler 2024; Johnson, Silber, and Darling 2024). Hence, the polling community should engage in coordinated public engagement for the future of survey research and address divisive issues like polarization and skewed perceptions. Despite the prevalence of educative programs, few target the general public, and their effectiveness has not been investigated. We devised three trainings and studied their causal effects in a longitudinal experiment. Our theory-informed modules exploited diverse strategies beyond the information-deficit approaches (Kuru, Pasek, and Traugott 2020a; Stadtmüller, Silber, and Beuthner 2022). We offered a novel theoretical pathway for conditioning the public perceptions of polls and public opinion broadly construed (Herbst 1993). This has important theoretical and practical implications.

Without interventions, discerning high-quality polls is almost nonexistent. This finding contrasts with recent studies that showed people recognized, at least partly, the relative credibility between simultaneously presented polls (Kuru, Pasek, and Traugott 2020a) and consecutively presented polls (Stadtmüller, Silber, and Beuthner 2022). While these studies were conducted in different contexts, the current design arguably offers the most conservative testing of this question. Without intervention, we can safely conclude that only a tiny minority of the public pay attention, let alone critically engage with, methodological details. This is not surprising.

### Main and Conditional Effects of Interventions

The interventions are effective. We found that interventions instilled a more critical mindset, especially the inoculation strategy, which worked across the board. We demonstrated an intervention that moves the needle meaningfully. These effects could accumulate over time and across contexts to foster a more accurate communication of public opinion, thereby limiting misunderstandings and misperceptions. More specifically, we showed the combined benefit of interactivity and motivational triggers compared to passive information delivery alone or information delivery with interactivity. This shows that engagement, feedback, and motivating individuals can operate together in cultivating survey literacy to the extent that it can shape evaluations of polls encountered later. These results speak to the efficacy of engaged learning (Garfield and Chance 2000; Oh and Sundar 2015) and motivational triggers (Compton 2021) and particularly “active inoculation” (Green, McShane, and Swinbourne 2022; Saleh et al. 2024). As the combination of information and interactivity was not as effective without motivational triggers, we can conclude that motivational triggers with engagement are more crucial than interactivity alone (cf. Green et al. 2022). Since we did not test a “passive inoculation” intervention, we cannot know if this added benefit of motivational triggers can also be observed in the absence of interactivity.

At large, our multi-component methods trainings add to the repertoire of more direct forms of inoculation on public opinion (Niederdeppe, Gollust, and Barry 2014). This strategy can be exploited in highly polarized contexts to understand the dynamics of belief change and mitigate misinformed/polarized opinions (cf. Voelkel et al. 2023; Dias et al. 2024) by customizing components as needed. For example, motivational triggers can be utilized to counteract motivated reasoning in public opinion perceptions (Nir 2011).

Individuals differed in notable ways in their uptake. Interventions are generally more effective among those with greater preexisting abilities (Golumbic et al. 2022). High-education respondents in intervention groups were more critical of low-quality polls. On the other hand, among those with greater science literacy, both control and intervention participants were more critical of low-quality polls. Practically, we can characterize this as a *rich-gets-richer* scenario, similar to the exacerbation of the “knowledge gap” at the macro level (Gaziano and Gaziano 2014). However, inoculation makes an improvement for all, irrespective of preexisting abilities. Results on scientific literacy identified a small segment who, even without an intervention, assessed polls critically. This gives additional context to the null main effect of poll quality among control respondents.

Consistent with prior work (Madson and Hillygus 2020), people discredited polls that delivered results that contradicted their views. However, this biased processing did not moderate the effects of poll quality or interventions. If individuals had rejected polls due to prior beliefs, then this would have raised questions about the value of these interventions over the long term when it comes to one of the most persistent biases in public opinion perceptions (Madson and Hillygus 2020; Neyazi and Kuru 2022; Johnson, Silber, and Darling 2024). While we can conclude that motivational bias in this context did not counteract intervention effects, the absence of a significant moderation should not be interpreted as a conclusive “correction” effect (i.e., mitigating motivated reasoning). We should focus on even more polarizing contexts to investigate the potential for bias mitigation.

We had worried that training could induce general skepticism (spillover effects), as seen in other contexts (Guess et al. 2020), but this did not occur. The interventions (vs. the control) did not specifically increase negative evaluations of polls. There was a slight increase in negative evaluations across all returning participants, including the control group. This was a within-subject effect (Supplementary Material section 7) and not due to attrition (Supplementary Material section 6). This signals a tension between micro vs. macro considerations: a slight increase in skepticism toward polls, if it exists at all, might be worth tolerating to cultivate critical evaluation of specific polls encountered.

### Practical Implications and the Future of Survey Research

Testing the effectiveness of interventions is crucial for evaluating and strategizing the polling community’s transparency and education efforts (AAPOR 2015). Our findings strongly support the promise of training, engaging, and motivating the public about surveys. These can include interactive and explanatory quizzes as well as motivational prompts. Interventions can be customized by JITAIs (just-in-time-adaptive interventions) (Hardeman et al. 2019), targeting differing education levels. Media organizations can provide links to such training modules or embed them into gamified surveys (Keusch and Zhang 2017).

To contextualize the implementation of interventions (IJzerman et al. 2020), we conclude with a qualitative review of the existing education resources. We listed these educational activities in table 5 and identified their general characteristics. As seen in the bottom panel of table 5, there is a greater need for more public-oriented and publicly accessible education, implementing diverse strategies, and more coordination across stakeholders. Our empirical study showed the importance of engaging the public through diverse strategies including interactive and motivational components, which is possible through more coordination among pollsters, scholars, data journalists, and digital platform designers.

**Table 5.** A list of the contemporary public education and training efforts about survey methods based on qualitative review.

Organization, entity, or resource	Description and links	Target audience and reach
American Association for Public Opinion Research (AAPOR)	(1) Online Education/Webinars provide methods training. <https://aapor.org/media/webinars/> (2) Task Force Reports provide weight-of-evidence and expert-consensus assessments on cutting edge issues on polling. <https://www-archive.aapor.org/Education-Resources/Reports.aspx> (3) Standards and Best Practices documents. <https://aapor.org/standards-and-ethics/best-practices/#1668111466330-2e542b06-fd59> (4) AAPOR Cheat Sheet for Journalists in For Media section provides guides for reporters. <https://www-archive.aapor.org/Education-Resources/For-Media.aspx> (5) AAPOR Election Polling Resources is specifically about understanding election polls. <https://aapor.org/publications-resources/education-resources/election-polling-resources/>	Portfolio includes different target audiences including experts (webinars, best practices guides, reports), journalists (cheat sheets, task force reports), and to a lesser extent, the general public. Most materials and reports are public.
American Marketing Association (AMA)	(1) Marketing Research Playbook section in the website provides resources, tool kits, templates. <https://www.ama.org/toolkits/market-research-playbook/>	The focus is assessed to be geared more toward experts, and materials are for members.
American Statistical Association (ASA)	(1) Education section in the website provides guides and pedagogy for statistics in general. <https://www.amstat.org/education>	This is a resource for educators and students that is publicly available.
British Polling Council (BPC)	(1) Guide for Journalists in the website provides tips and information about polls. <http://www.britishpollingcouncil.org/opinion-polls-guidance-for-journalists/>	This is a short guide for journalists that is publicly available.
European Society for Opinion and Marketing Research (ESOMAR)	(1) ESOMAR Academy has comprehensive trainings on survey design. <https://esomar.org/academy>	The target audience is assessed to be geared more toward experts and researchers; materials are mostly not publicly accessible.
Eurostat	(1) Statistics Explained section in the website provides contextual explanations on key socioeconomic and social indicators in Europe. <https://ec.europa.eu/eurostat/statistics-explained/index.php?title=Main_Page> (2) The European Statistical Training Program <https://cros.ec.europa.eu/book-page/estp-training-offer>	The publicly available materials can be helpful for experts, journalists, students, and the public as well. Training program is assessed to be more geared toward experts and journalists.
Gallup	(1) Methodology Blog at Gallup provides information and research about polling methods in general (e.g., survey scales, asking inclusive questions about gender). <https://news.gallup.com/topic/methodologyblog.aspx>	Audience is assessed to be geared more toward experts but they are publicly available.
General Social Survey (GSS) at NORC at the University of Chicago	(1) The General Social Survey Video Series section in the website provides publicly available tutorials on methodological topics such as survey questionnaire design, sampling, total survey error. <https://gss.norc.org/pages/videos.aspx> (2) The materials are also on social media (YouTube) for larger public reach. <https://www.youtube.com/@GSS_NORC/videos>	The primary target is assessed to be geared more toward researchers and experts. Resources are publicly available.
Global Barometer Surveys (GBS)^a^	AfroBarometer, ArabBarometer, AsianBarometer, ICSR-Eurasia Barometer, Latinobarómetro (1) Analysis tools <https://www.globalbarometer.net/analysis> <https://www.arabbarometer.org/survey-data/data-analysis-tool/> (2) Guide videos <https://www.youtube.com/watch?v=BlsD-biInsI> (3) AfroBarometer Summer Schools <https://www.afrobarometer.org/impact/capacity-building/afrobarometer-summer-school/>	Materials are assessed to be geared more toward experts, researchers, and students.
Guide books about polls for the general population	(1) Asher, H. (2016). *Polling and the Public: What Every Citizen Should Know.* CQ Press. (2) Salvanto, A. (2019). *Where Did You Get This Number? A Pollster’s Guide to Making Sense of the World*. Simon & Schuster. (3) Traugott, M. W., & Lavrakas, P. J. (2016). *The Voter’s Guide to Election Polls* (5th ed.). Lulu Publishing Services.	Books provide comprehensive account of survey methods for the public. The primary audience in these resources is assessed to be the general public and journalists.
Institute for Social Research (ISR), University of Michigan	Training programs provide methodological training for designing, implementing, and analyzing surveys. (1) Survey Research Center (SRC) (2) Inter-University Consortium for Political and Social Research (ICPSR)	Researchers, experts, students, and journalists can attend these formal trainings. They are not publicly available.
International Association for Statistical Education	Trainings and workshops about applied statistics <http://iase-web.org/> (1) International Statistical Literacy Project <http://iase-web.org/islp/>	Primary target is assessed to be geared toward educators (pedagogy) and students.
Latin American Public Opinion Project (LAPOP), Vanderbilt University	On-site training programs, summer schools, and other periodic written resources: (1) <https://www.vanderbilt.edu/lapop/summer_research_in_survey_methodology/summerschoolresearchmethodology.php>	Resources are assessed to be geared more toward researchers, experts, and students. Reports such as “*Insights Series”* are public.
News media and data journalism websites/blogs	Explanatory, interactive data journalism stories, tools, and educative videos in news stories and data journalism outlets. Examples: (1) FiveThirtyEight (Currently archived website): Methodology section in the website provides explanations for polling averages computation, forecasting models, and the like. <https://fivethirtyeight.com/tag/methodology/> (2) *New York Times* (*NYT*) Upshot: Search “methodology” for explanatory articles on methods on polling. <https://www.nytimes.com/section/upshot> (3) “What is going on in this graph?” Collaboration between NYT and American Statistical Association <https://www.nytimes.com/column/whats-going-on-in-this-graph> (4) *USA Today* and other explanatory educative stories <https://www.youtube.com/watch?v=UaE-0T04N4c>	These are primarily for the general news-reading public. “*What is going on in this graph?”* is a journalistic project geared toward students.
Pew Research Center	(1) Tips and guides about polling basics <https://medium.com/pew-research-center-decoded/5-tips-for-writing-about-polls-9cb0596ff28> and <https://www.pewresearch.org/course/public-opinion-polling-basics/> (2) Polling Knowledge: Interactive quiz about polling methods in general (8 items) as well as specific issues (e.g., 2020 the US election and polls) <https://www.pewresearch.org/methods/quiz/how-much-do-you-know-about-public-opinion-polling/> (3) Methods 101 video series on the YouTube channel: explanatory videos about polling methods (e.g., nonprobability surveys, question wording, sampling, mode effects) <https://www.youtube.com/@pewresearch/search?query=methods101>	The portfolio is assessed to be geared toward researchers, experts, students, teachers, journalists, and the general public. Available on social media in video formats (YouTube) with hundreds of thousands of view counts and interactive tools (quiz). There is particular attention to the general public as well.
Poynter	(1) “Understanding and Interpreting Opinion Polls” Poynter partnered with AAPOR in 2008 through 2016 to provide self-directed course to reporters. <https://www.poynter.org/ethics-trust/2018/this-organization-helps-reporters-cover-public-opinion-research-and-polling-more-accurately/> (2) Guidelines <https://www.poynter.org/educators-students/2016/5-guidelines-for-writing-about-poll-numbers/>	The resources are assessed to be geared more toward journalists, with some of the content publicly available.
Roper Center for Public Opinion Research	(1) Learn & Teach section in the website provides training and information such as specific guides, history, and basics of polling. <https://ropercenter.cornell.edu/learn> <https://ropercenter.cornell.edu/polling-and-public-opinion/polling-fundamentals> (2) Classroom materials for educators < https://ropercenter.cornell.edu/learnteach/classroom-materials-educators>	The resources are assessed to be geared toward experts, journalists, teachers, students, and the general public.
The Journalist’s Resource	(1) A large list of guides and commentary about polling coverage <https://journalistsresource.org/?s=polls> Examples: <https://journalistsresource.org/politics-and-government/public-opinion-polls-tips-journalists/> <https://journalistsresource.org/politics-and-government/polling-fundamentals-journalists/>	The primary audience is journalists.
US Census Bureau	(1) Statistics in Schools Program under Education section in the website offers comprehensive trainings and tools for statistics in general, but with a focus on counting and surveying. <https://www.census.gov/schools> <https://www.census.gov/programs-surveys/sis/resources/videos.html>	The portfolio is for researchers, experts, students, teachers, journalists, and the general public. It has particular pedagogical content focus for teachers.
World Association for Public Opinion Research (WAPOR)	(1) Webinars and trainings about polling methods and research <https://www.youtube.com/@worldassociationforpublico783/videos> Also available on YouTube channel, the webinars include methodological focus too. <https://www.youtube.com/@worldassociationforpublico783/videos> (2) Resources for journalists: a compilation of resources at WAPOR and elsewhere <https://wapor.org/resources/resources-for-journalists/> (3) Online course for journalists <https://wapor.org/online-course-for-journalists/>	The portfolio is assessed to be geared toward researchers, experts, students, teachers, journalists, and the general public.
World Values Survey (WVS)	(1) Teaching, coaching and workshops: examples include workshops in “Conferences and Workshops” tab and methods information in “What we do” tab in the website: <https://www.worldvaluessurvey.org/WVSContents.jsp>	These trainings target experts and students mostly.
**General Observations** Source: Most of the activities are led by organizations independently and only a few are collaborations, such as the NYT and American Statistical Association collaboration about the “*What is going on in this graph?*” series. Availability: The resources target students, practitioners, media professionals, and academics more than the nonexpert/nonstudent general public. While most of these activities are publicly available, they are generally hosted on organizational websites or locations. Most websites require memberships and accounts. Only a few have a broader reach on social media (e.g., Pew Research Center, publicly available), in the newspapers (e.g., NYT, limited free views and subscription based), or in the guidebooks listed above (for purchase or through library access). Content: Most educational programs require some level of statistical and methodological background and take at least a few hours of concentrated work. Such lengthy training may not be scalable for general public interventions, as most people lack time or resources. Finally, a few resources have interactive elements, like the YouTube videos (e.g., NORC) and quizzing (e.g., Pew Research Center).		

*Note*: Resources are listed in alphabetical order. The aim of this qualitative review was (1) to provide a more systematic assessment of survey methods training available to the general public, (2) obtain insights/examples for the design of the interventions in this study, and (3) contextualize the experimental results via their practical relevance and implications. The qualitative review consists of (1) the search strategy to determine sources, (2) the observation of survey methodology related resources in various diverse formats such as training, scientific information, books, workshops, interventions, and social media content (to the extent possible, in terms of access) and their descriptions in the sources identified, and (3) the determination of patterns in the resources (nature of source and the content, their availability and relevance to the general public). Search steps involved the review of public-opinion-related organizational websites, academic journals, academic summer training programs/workshops, news media, polling organizations and research centers, keyword search in academic and general search engines, and snowball technique in which new resources are discovered in the cross-references in these initial sources. Limitations include reliance on English language content and not covering all efforts given the space limitations. For detailed methodological details about this qualitative review, including the search and inclusion criteria and other similar trainings not included in the main table, please see Supplementary Material section 1.

a See methodological details for the distinction between the parent and affiliated regional organizations.

This study provides insights into the future of polls and survey research amid the contemporary social and technological changes and challenges (Krosnick et al. 2015). The credibility of survey research is central to the conduct of polls, too, with some calling for its inclusion in the Total Survey Error framework (Groves and Lyberg 2010). Such a consideration requires a well-planned communication of polling evidence to the public (Jamieson et al. 2023; Bailey 2024). Criteria for robust quality will face more debates, given declining response rates and transparency issues (Jerit and Barabas 2023), professional survey-takers (Hohenberg et al. 2025), and the generative AI and “synthetic” samples (Kennedy et al. 2022). These issues will continue to foster debates about the credibility and societal role of polls in democratic politics in particular and survey-based social science research and administrative statistics at large, which can be effectively managed through public engagement and coordinated educative interventions.

The effectiveness of interventions should be tracked for longer periods. Future studies can extend this work to election polls and more polarizing issues. Scholars can develop and test trainings about novel metrics of public opinion, such as political forecasts, which the public is even less knowledgeable and confused about (Westwood, Messing, and Lelkes 2020; Barnfield et al. 2025). Individual difference moderators and perceptions of issues can be examined with larger samples. Field experiments are needed to enhance the applicability of findings and further diversify respondents. It is crucial that these effects are investigated in probability-based samples, to account for potential self-selection biases in opt-in panels. Our “omnibus approach” in manipulating methodological quality reduces the applicability of findings for more mixed methodological quality scenarios.

## Supplementary Material

nfag006_Supplementary_Data

## Data Availability

Replication data and documentation are available at https://doi.org/10.7910/DVN/AXMNFO.

## Appendix A. AAPOR-Required Disclosure Elements

**First Data Source:** This study is an online survey-based data collection (longitudinal online survey experiment) by the authors through a company—Bilendi & respondi.

**Data Collection Strategy:** Data collection strategy nonprobability-based online survey.

**Research Sponsor and Conductor:** The funding for this study came to the author from the National University of Singapore [R-124-000-119-133/A-0003775-00-00]. The research was conducted by the author.

**Measurement Tools/Instruments:** All measure details are provided in table 1 already or in Supplementary Material section 1.

**Population Under Study:** As stated in the Methods section, the population under study is the general population—legal adult (aged between 21 and 80) resident individuals living in Singapore at the time of data collection (December 2023). There are no further restrictions on the sample.

**Methods Used to Generate and Recruit the Sample:** The study is based on a nonprobability-based online data collection. We conducted a two-wave longitudinal online experiment with a nonprobability sample of individuals (legal adults in Singapore: age 21 and older) with *Blendi & respondi*. *Blendi & respondi* offers voluntary survey participation with extensive validation, including a double-opt-in registration process. It utilizes a variety of varied sources of recruitment: search engine advertising, database exchange, partnerships for specific targets, and emailing campaigns (see Supplementary Material section 1 for more details). Quotas were used to approximate demographic representation as much as possible. We coordinated with the survey company to put the following demographic quotas to ensure a balanced representation of the population in Singapore as much as possible given the limitations of the online nonprobability-based sample: age [“21–24,” N = 131; “25–34,” N = 598; “35–44,” N = 586; “45–54,” N = 435; “55–80,” N = 330], education [“First group” (includes all lower-education groups in “Polytechnic” or less), N = 800; “Second group” (includes all medium and higher-education groups in “Bachelor’s degree” and more), N = 1,280], sex [“Male” and “Other” categories, N = 990; “Female,” N = 1,090”], race [“Chinese,” N = 1,695; “Malay,” N = 195; “Indian,” N = 95; “Other,” N = 95], and income [“up to 3,000 SGD,” N = 290; “3,000–7,000 SGD,” N = 675; “7,000–11,000 SGD,” N = 525; “11,000–15,000 SGD,” N = 300; “15,000 and above SGD,” N = 290]. Quotas were applied only for Wave 1, as the second (follow-up) survey aimed to maximize retention of returning respondents. Toward the end of W1, quotas were “relaxed” (slightly adjusted) to speed up and finalize data collection. Quota numbers do not exactly match the sample size obtained because of additional (bonus) participants the company provided and the low-quality respondents the researchers would identify and reject (as detailed above). Overall, quotas provided some level of match to the population demographics. Note that this is an experiment (with successful randomization) where the focus is not on population parameters. We noted the limitations of the sample below and in the manuscript. *Blendi & respondi* follows ICC/ESOMAR Codes for its regulations. It includes 300+ criteria for quotas and segmentation in the entire panel and conducts random selection of respondents within quotas for specific surveys. Inattentive respondents such as speeders and nonsense response providers to open-ended questions are actively identified and sanctioned if there are repeated violations. For compensation, participants receive points that are redeemable in a gift catalogue, into cash rewards or, alternatively, they may donate the amount to a selected organization. This panel is invite-only recruitment (email and human verification) and does not involve river sampling.

**Method(s) and Mode(s) of Data Collection:** This is a web survey conducted in English.

**Dates of Data Collection:** Wave 1 was collected between December 5 and 14, 2023, while W2 was collected between December 12 and 18, 2023.

**Sample Size:** The sample size for Wave 1 (W1) was N = 2,062, and for Wave 2 (W2), it was N = 1,278. The panel retention rate was 62 percent.

**Whether and How the Data Were Weighted:** There are no weights.

**How the Data Were Processed and Procedures to Ensure Data Quality:** We recorded N = 3,326 attempts (opening of survey link) for W1 and N = 1,291 for W2. The gap between good completes and link openings, which is N = 1,264 for W1 and N = 13 for W2, indicates those who did not complete the survey either by themselves (broke-off, N = 392), rejected consent (N = 101) and honesty pledges (N = 88), or failed attention checks (first one N = 351, second one N = 332). We removed eight speeders, three laggards, and 24 respondents who withdrew consent (Supplementary Material section 1). The effective sample size for W1 is N = 2,027, and the longitudinal sample is N = 1,076. Details are in the Methods section and in Supplementary Material section 1. This research did not use any automated or artificial intelligence at any stage, including coding, analysis, literature review, and writing of the research report.

**IF A PANEL WAS USED: Panel Description:** Relevant details are already provided in the two sections above, titled “Methods Used to Generate and Recruit the Sample” and “How the Data Were Processed and Procedures to Ensure Data Quality.”

**IF ELIGIBILITY SCREENING WAS DONE:** Screening criteria and process: those below 21 years old (legal adult age in Singapore) and above 80, those who did not agree to the consent form, those who did not agree to provide honesty pledge, those who do not reside in Singapore at the time of data collection, those who failed attention checks, speeders, and those who withdrew consent after the debriefing (as stated above) were screened out.

**Study Stimuli:** The experimental manipulations involve detailed trainings and take substantive space, but all original content is provided in Supplementary Material section 1.

**Dispositions or Response or Participation Rates:** While there is no true sampling response rate, we recorded N = 3,326 attempts (opening of survey link) for W1 and N = 1,291 for W2. The gap between good completes and link openings, which is N = 1,264 for W1 and N = 13 for W2, indicates those who did not complete the survey either by themselves (broke-off, N = 392), rejected consent (N = 101) and honesty pledges (N = 88), or failed attention checks (first one N = 351, second one N = 332). See SM1-E and G for attention checks. This corresponds to a response rate of 62 percent in the recruitment sample (W1).

**Sample Sizes:** All models’ specific sample sizes are reported in the associated Results section tables. Model sample sizes differ based on modeling strategy; most results are reported on the entire sample, split-issue analyses provided in Model 3 in table 2 and Model 4A and 4B in table 4, as indicated in those tables, are based on half/split sample for the specific public opinion issues. For the income variable (which is not a control variable in the main models, as it was not found to be biased in balance tests for randomization check), the missing scores for N = 51 respondents who chose “Do not want to respond” were imputed with the median income value 5.

**Measurement and Model Specification:** Modelling details are explained in the analytical strategy section in the Methods section, and all code is provided in the R file at the POQ Harvard Dataverse, publicly available.

**A General Statement Acknowledging Limitations of the Design and Data Collection:** The experiment is based on a nonprobability-based online data collection; there is no sampling frame or true response rate. The data is based on a single country. It is a two-wave longitudinal study, and not all participants in Wave 1 participated in Wave 2.
