# Supplementary material for: Conditioning Public Opinion Perceptions by “Survey Methods 101”: Informing, Engaging, and Motivating Individuals for Critical Processing of Public Opinion Polls
Source: Public Opin Q. 2026 Mar 22;90(2):399–450. doi: 10.1093/poq/nfag006 (PMC13081209; doi:10.1093/poq/nfag006)
Supplement: nfag006_Supplementary_Data [file nfag006_supplementary_data.pdf]

Supplementary Materials for

Conditioning Public Opinion Perceptions by "Survey Methods 101": Informing, Engaging, and Motivating Individuals for Critical Processing of Public Opinion Polls

Ozan Kuru
Assistant Professor, National University of Singapore
Correspondence to: okuru@nus.edu.sg

Table of Contents

*You can click on "Page" hyperlinks below to navigate the sections and "TOC" (Table of Contents) to come back to the first page below.

1. **Supplementary Material 1:** Design and Methods Details
    a. Methodological Details for the Qualitative Review **[Page]**
    b. Limits of Post-hoc Corrections **[Page]**
    c. Rationale for Adopting the Omnibus (vs. Granular) Approach to Operationalizing Public Perceptions of Methodological Quality **[Page]**
    d. Considerations and Further Rationale in Designing of Conditions and Messages **[Page]**
    e. Power Analysis, Sample Size Considerations, and Additional Sampling Details **[Page]**
    f. All Manipulations and Messages **[Page]**
    g. Other Measures and Full Details **[Page]**
    h. Descriptive Statistics **[Page]**
2. **Supplementary Material 2:** Preregistration and Deviations from Preregistration Explained **[Page]**
3. **Supplementary Material 3:** Equivalence of Conditions and Cell Balance Tests **[Page]**
4. **Supplementary Material 4:** Manipulation Checks **[Page]**
5. **Supplementary Material 5:** Testing Alternative Models Based on Inclusion of Covariates
    a. Results with inclusion of Pre-test Imbalanced Covariates (as reported in the manuscript, with covariate coefficients) **[Page]**
    b. Results with No Control/Covariate Variables **[Page]**
    c. Results with All/Additional/Further Theoretically and Methodologically Relevant Control/Covariate Variables [**Page**]
    d. Details for RQ1 and RQ2 Results **[Page]**
6. **Supplementary Material 6:** Robustness Checks
    a. Attrition Analysis **[Page]**
    b. Longitudinal Interference Checks **[Page]**
7. **Supplementary Material 7:** Other Preregistered and Exploratory Analyses
    a. Shifts in General Evaluations of Polls across Time **[Page]**
8. **The References for Supplementary Materials** [Page]

Supplementary Material 1: Design and Methods Details

A. Methodological Details for the Qualitative Review [click here to return to TOC]

The aim of the review was to be comprehensive in the selection of all educational efforts about survey methods and be clear with inclusion criteria as much as possible given the heterogeneity in the type, reach, and nature of content.

a

1. As a preliminary step, we started exploring the leading organizations WAPOR and AAPOR on public opinion. We have carefully checked all tabs of the websites of these organizations to detect educational activities.
2. Next, we brainstormed and listed a variety of other organizations and institutions to check. During the review, we came across new organizations and activities that were mentioned in the first set of sources (snowballing, cross-referencing), which were then added to our list.
3. We also searched the publication archives of the following journals: Public Opinion Quarterly, International Journal of Public Opinion Research, Survey Practice, Journal of Survey Statistics and Methodology.
    a. These searches mostly returned methodological literature such as interviewing training or training students about methods.
4. We then proceeded to other news media, research centers, and university sources.
5. We also used academic search engines (Google Scholar, Web of Science) but no relevant results emerged. Keywords: (survey OR poll) AND (methods OR methodology OR technique OR techniques OR research OR data) AND (training OR intervention OR education OR workshop OR module).
    a. No new public education activities or institutions have been detected with this strategy as these public education activities do not usually end up as academic publications.
6. Other similar efforts – not included in the main table:
    a. University survey research support centers. As many universities have these support units and they had minimal or no strategic wider reach efforts, we have not included such initiatives For example, Duke Initiative on Survey Methodology (https://dism.duke.edu/) had good online survey methods resources but these were geared towards the host university.
    b. There are numerous other international summer training schools such as the International Political Science Association Summer Schools (https://www.ipsa.org/ipsa-summer-schools) some of which include methods training (https://fass.nus.edu.sg/methods-school/home/about/), the RECSM Summer Methods School at Research and Expertise Centre for Survey Methodology (RECSM) at the Universitat Pompeu Fabra Barcelona (https://www.upf.edu/web/survey/2025-summer-methods-school) and the Summer School: Survey Methodology Training at GESIS - Leibniz-Institut für Sozialwissenschaften (https://training.gesis.org/?site=pDetails&pID=0x95D2949CC24E4C3E87DE9AB08493E1E1). These methods schools have also focus on survey methods.

   c. Survey research company trainings are not included, as these are mostly geared towards customers or commercial. However, some of these are partially publicly available and comprehensive. Examples:
      i. Kantar's Survey Design Training Modules - https://www.kantar.com/campaigns/pf/survey-design-training-modules
      ii. Qualtrics' Training Certificate Program and Online Guides - https://www.qualtrics.com/training/
7. Grouping decisions: Global Barometer Surveys (GBS) is entered as a single entry despite the collective organizations have both an overarching parent organization (https://www.globalbarometer.net/about_bg) as well as independent websites for the regional centers. Given the coordinated parent organization and that not all regional hubs had information about the methodological training information on their platforms, they were entered together collectively for the purposes of this table instead of listing/including only those who included this information on their websites.
8. Limitations:
   a. Search has been limited to English language content.
   b. The latest review update date is June 2024. As there might be activities we have missed or new ones that might emerge shortly, after publication, we hope to have an updated online live list of Table 1 as a repository.
9. Other considerations:
   a. We received earlier feedback that we can also provide individual critical evaluations and recommendations for each organization and initiative listed in our review. We decided against this as this is beyond the scope of this study and our strategies was identifying some common patterns and highlighting notable examples instead of singling out specific organization or initiatives. We believe this is a more productive and measured approach.
   b. The inclusion in the main table also paid attention to the diversity of content covered. For instance, a journalistic organization website or books intended for the general public.

B. Limits of Post-hoc Corrections [click here to return to TOC]

There are further limitations of post-hoc corrections. Pre-emptive literacy training in other contexts, in general, is more effective than post-hoc corrections like debunking and fact-checking (McDougall, 2019). Also, post-hoc corrections are generally small-scale, such that, they do not have the necessary bandwidth (time and space) to deliver methodological concepts to the public effectively. For example, a concept like margin of error is hard to cover in the context of a fact-check correction or a news story reporting on poll results. Additionally, this bandwidth problem also limits the diversity of tactics that can be employed to only information delivery; illustrating the heavy reliance on the information deficit model in these correction attempts (cf. Bubela et al., 2009), which overlooks other dimensions such as interactivity and motivations. Finally, post-hoc interventions may also fuel "illusory truth effects" due to the repetition of misleading or poor-quality content being corrected (Udry & Barber, 2024) or may even, to a limited extent, elicit "backfire effects" in the context of significantly polarizing political issues (Williams-Ceci et al., 2024; Wood & Porter, 2019).

C. Rationale for Adopting the Omnibus (vs. Granular) Approach to Operationalizing Public Perceptions of Methodological Quality [click here to return to TOC]

As mentioned in the paper, we distinguish between omnibus vs. granular approach to operationalizing public perceptions of poll methods quality. We list and explain in detail three important reasons for choosing the omnibus approach. We believe this approach is more in line with how low vs. high-quality surveys differ, as they usually differ on several aspects that tend to go together and hence it is more ecologically valid. Relatedly, some aspects by themselves, such as higher sample size, may be misleading (Bradley et al., 2021). 2) Members of the public are more likely to have a generalized summary evaluation either depending on one, few, or all methods details as much as possible and are less likely to compare and weigh different details separately (i.e. relative importance of sample type vs. response rate) as they have less expertise and time than survey methodologists. 3) Finally, studies that manipulated all different combinations of methodological aspects ended up having at least some ecologically less valid conditions and because of the large number of experimental groups, they had to show multiple polls – consecutively – to each of their respondents, potentially inducing demand effects (Stadtmüller et al., 2022).

D. Considerations and Further Rationale in Designing of Conditions and Messages [click here to return to TOC]

Manipulations
Wave 1: none (control) vs. literacy passive vs. literacy active vs. inoculation
Condition W1-1: Control (baseball)
Condition W1-2: training (passive literacy)
Condition W1-3: training + quiz (active literacy)
Condition W1-4: training + quiz + warning (inoculation)
Wave 2: result X methodological quality X issue replication
Condition 1 (Low quality, Majority Concerned, Vaccine)
Condition 2 (High quality, Majority Concerned, Vaccine)
Condition 3 (Low quality, Majority Confident, Vaccine)
Condition 4 (High quality, Majority Confident, Vaccine)
Condition 5 (Low quality, Majority Support, AI)
Condition 6 (High quality, Majority Support, AI)
Condition 7 (Low quality, Majority Oppose, AI)
Condition 8 (High quality, Majority Oppose, AI)

- **Survey Language Mode: English.** While there are four official languages in Singapore, survey was conducted in English (in line with great majority of research conducted in Singapore) as Enlish is the education, business, and government language and about 95% of the population is literate in English. (Department of Statistics, 2020).
- **Control condition had an equivalent task:** Given there are 3 experimental conditions, we created a control in a way so that the amount of activity in it matches the average amount of activity in all three experimental conditions combined. For example, we asked 2 questions, 1 interest and 1 intention about baseball following the information delivery about baseball rules. To make it a comparable topic that involves some type of rules to be learned, we focused on baseball, with a nod to the *Signal and the Noise* book by Nate Silver.
- **What is a high methodological quality survey?** High-quality polls are mostly probability-based samples with larger sample sizes and response rates, and they provide the most accurate portraits of public opinion with a lower margin of errors (Baker et al., 2010). These stand in contrast to non-probability based polls, lower sample sizes (which especially increase margin of errors for population subgroups in the sample), lower response rates, and online opt-in samples (Mercer et al., 2024; Pasek, 2015).
- **Response rates in Manipulations:** We anticipate criticism that response rates are declining and high response rates in the high-quality poll experimental conditions are not very realistic. While response rates decrease in many countries around the world, what constitutes an acceptable and robust quality poll may be contested (Bailey, 2024). Yet, response rate is not the only indicator of methodological quality. What we have done is a proof of concept by operationalizing methodological quality as robust vs. poor as done in similar recent studies. Future educative interventions may easily integrate additional insights or updates to the characterization and limitations of response rates and other factors. The numbers given in our manipulations provide an opportunity to test individuals' attention to low vs. high response rates, relatively speaking. We also note that response rates vary a lot across countries. For example, while in the US, response rates are getting increasingly low (Leeper, 2019), in the context of empirical data collection, in Singapore, survey response rates are typically much higher. We think this is important and was the major reason in deciding on the number for the high response rate in the manipulations. For example, the National Health Survey 2022 (*MOH | National Population Health Survey 2022*, n.d.) has around 69% response rate while the 2022 Comprehensive Labour Force Survey (CLF) had an 85% response rate (*Comprehensive Labour Force Survey*, 2022). Response rates are similar in numerous other recent public opinion surveys. Finally, we note that falling response rates still do not reduce overall quality, and probability surveys still constitute the most accurate form of public opinion (MacInnis et al., 2018). We carefully weighed these different considerations during the design of the experimental messages, and in this case, when deciding on the numbers for response rates.
- **Design Considerations on Poll Results**: We intentionally kept all low-quality and high-quality polls showing a clear majority, but each had results in both directions, either showing majority support vs. majority opposition. This strategy omits inconclusive polls, such as those showing a close gap, with figures around 50%. We avoided showing such polls so that the margins of errors we report do not interfere with the interpretation. Whether poll results are clearly showing a directional result – because this is a state of public opinion issue (whether it is a consensus or polarizing issue) can be addressed in future research, studies can investigate how methodological quality may matter in such close-gap poll results.

- **Design Considerations on Time Gap between the Waves:** We designed the gap between Wave 1 and Wave 2 as a full 7 days. That is respondents from W1 were invited to the follow-up study full 7 days later (on the 8th day) after they completed W1. Recent work suggests that individual-level interventions such as inoculation tend to decay rapidly after 48 hours (Capewell et al., 2023). Given that this is the first longitudinal study in this area of work, we decided on a full 7 days delay as a balanced approach. Having 48 hours delayed W2 would have lowered the practical validity of our findings as it may be considered too short a gap. A week of gap time is a more notable and valuable test for educative interventions to observe their practical longevity beyond the high decay threshold of 48 hours the prior work documents. Future work should track and measure effects on time periods longer too.
- **Practical Insights for the Design of Interventions**
- We obtained and implemented the following practical and technical insights and examples from the qualitative review of existing survey methods education efforts:
    - 1) To design an online intervention of about 10 minutes (equivalent to a short online survey session and interventions in recent inoculation literature) that can be completed in one sitting by a wide portion of the general public. This was inspired after concluding that most educational efforts were substantive (at least lasting a few hours) and did not target the general public. General public does not have time and resources to allocate a few hours to our interventions. Then we decided our intervention length should not be longer than a short, self-administered, online survey length. We decided on a length of about targeting 10 minutes completion time at most (for the average respondent) based on these considerations.
    - 2) Do not assume no prior knowledge, provide very simple definitions and examples first, including the use of analogies (e.g. soup analogy for sampling, as mentioned in Pew Research Center content, and scaffolding in the delivery through focusing on increasingly more challenging concepts).
        - Scaffolding Principle in Trainings: For the interventions, we started with simple definitions (e.g. what is a survey), and examples, used analogies (mixing the soup example for representative sampling), and provided scaffolding towards more complex concepts (e.g. why a probability-based sample is more representative and scientific).
    - 3) Test the effects of both passive and active (interactive quiz) components as only a few educational efforts (e.g. Pew) utilized interactive quizzes accessible to the public. This atypical yet inspirational example underlines the importance of testing what is already widely adopted in the field (passive information delivery) vs. in what directions it could improve (diversification of strategies, including, at least, some hands-on interactive components).
- **Balancing Theoretical and Practical Considerations: Why not have a fourth (passive inoculation) intervention?**

  MACE Design: The current longitudinal experiment's design is informed by a mixed factorial approach with both between-subjects and within-subjects inferences obtained through fractional (Wave 1) and full (Wave 2) factorial designing of conditions. The study is a full factorial design at the Wave 2 and the longitudinal levels (fully symmetric crossing). This makes it a complex mixed design at the aggregate level. Given the primary research focus on Wave 1 interventions, we have carefully chosen an appropriate analytical approach:

At Wave 1, we used insights from the **fractional factorial design** strategy, a reduced version of full factorial design where one or more potential hypothetical cells from the crossing of manipulation factors are missing. Fractional factorial designs are widely used (accompanied by extensive methodological research on their strengths, value, and limitations) in behavioral intervention sciences for a variety of reasons, including 1) not all potential experimental conditions are theoretically interesting in any given context, 2) intervention effectiveness maximization necessitates removal of some conditions, and 3) practical considerations such as budget and statistical power are taken into account pre-emptively by reducing one or more possible cells (Collins et al., 2009, 2014, 2024; Dziak et al., 2012; Watkins & Newbold, 2020). **We used the fractional factorial approach in synthesizing the two theoretical dimensions (interactivity and motivation) to inform the design of the three interventions** (one single component and two multi-component full interventions). One cell out of the four theoretical possibilities was excluded (i.e., “passive intervention with motivational trigger,” which can also be characterized as “passive inoculation”). The increasing dosage regime aims to find the most effective intervention with the least resources – as in “intervention optimization” (Collins et al., 2024). This strategy thus compares interventions that progressively/consecutively build on each other (i.e., the second intervention having all components in the first one and, one additional component, and so on). This approach prioritizes practical and ecological validity considerations over purely theoretical ones (i.e., testing all theoretical possibilities, as in a full factorial approach), thereby carefully balancing internal and external/ecological validity.

Comparing the three interventions against each other and a true control condition, we thereby effectively treat (**analytical approach and inferences obtained**) W1 conditions (all four of them) as a “**multiple-arm comparative experiment**” (***MACE; “a k-arm experiment involving k-1 different forms of treatment and a control.”***) (Collins et al., 2014). This approach is appropriate for the research purpose of

1) determining whether interventions are effective (against control) and

2) evaluating which of the three interventions is the most effective (in comparison to each other).

- **Figure 1.** Theoretical Crossing of Two Dimensions

	Information (A)	Motivation (C)
Passive (A)	Passive Literacy Training<br>A	Passive Inoculation<br>A + C
Active (B)	Active Literacy Training<br>A + B	Active inoculation<br>A + B + C

- **Note:** Passive information is baseline, hence this is why both “passive” and “information” of them are labelled A. Theoretically and practically, we have to have at least passive information in an intervention. For control (no intervention condition), unrelated information is provided (rules of baseball).
- **Figure 2.** Conditions Designed in the Current Study’s Empirical Part

Control (C1)	Passive Literacy Training (C2)	Active Literacy Training (C3)	(Active) Inoculation (C4)
Unrelated training	Information (A)	Information (A)	Information (A)
		Quiz (B)	Quiz (B)
			Forewarning (C)

- Practically, focusing only on the three interventions provides consistency with "the increasing dosage regime" approach: "A" < "A + B" < "A + B + C" (Passive literacy < Active literacy < Active inoculation).
- Theoretically, it is not clear if "A + B" or "A + C" would be more effective, this is an open research question for future research. Within this increasing dosage principle, the relative effectiveness of the fourth possible theoretical combination of "passive + motivation" is not clear with respect to "active + information" combination. There is no prior work or clear rationale for suggesting whether interactivity or motivation matter more. Yet, findings on the passive vs. active distinction among inoculation interventions do not always show an added benefit of interactivity (Green et al., 2022), although we should note that the Green et al. (2022) study does not compare absence or presence of motivation.
- Note that information is the necessary baseline, without information, we cannot have an independent forewarning (motivational trigger) in inoculation and we cannot employ an interactive quiz without indirectly providing respondents with information:
    - If trainees only answered the quiz, this would not be an intervention; hence they must at least be provided the correct answers with explanation following their response, which would introduce information by design. That possibility does not make sense in terms of ecological and external validity.
    - If trainees were only warned about misleading polls, this would again indirectly provide information in order to be a realistic intervention, because warning should include explanations on what a misleading poll is, which is effectively information provision. While there is some research that focus on the effects of "misinformation warnings," (van der Meer et al., 2023; Williams-Ceci et al., 2024) those approaches do not clarify the absence or presence of any underlying informational element in their design, and they do not constitute an example of inoculation either.
        - The motivational part (forewarning) in inoculation has some inherent active features as a motivational trigger. Hence, it is ecologically less valid to have a truly passive + motivational intervention (passive inoculation). This is especially true for technique-based inoculations. Also, recent inoculation research extensively shifted the focus to investigate active inoculation due to its promise and practical impact through games and exercises (Basol et al., 2020b). Finally, as a motivational trigger mobilizing cognitive resources, forewarning is inherently interactive (Compton, 2021), thus rendering "passive inoculation" theoretically and practically less coherent and distinctive (Basol et al., 2020; Compton, 2021).

  - Hence, not all theoretical combinations are ecologically and externally very valid. Just because we can cross multiple dimensions does not mean all resulting cross-factorial cells are meaningful in real life.
  - Finally, despite all these concerns, while this fourth theoretical combination can be investigated in future research as an open research question, the current study did not include it due also to statistical power too. Adding one W1 intervention would have increased W1 conditions from 4 to 5, and total number of conditions (crossing by 8 conditions at W2) from 32 to 40. Hence, limited resources rationale also supports our decision not to test this theoretical fourth possibility condition (passive inoculation).

- **Passive vs. Active Distinction in Information Engagement (but not in Motivational Component):** Passive literacy training tests if information delivery alone would be effective in recognizing methodological quality indicators. Active literacy training assumes interactivity may enhance the learning of survey methods through hands-on exercise and feedback. Finally, inoculation tests if motivational triggers might have additional benefits. We did not further differentiate between passive vs. active inoculation in the current study because of low ecological validity and inapplicability of making its distinct component - motivational trigger (forewarning) - either passive vs. active. Also, the passive vs. active distinction in inoculation literature is applied collectively in inoculation research (Green et al., 2022; Saleh et al., 2021), not granularly about the distinct informational or motivational components; and investigating this distinction is not of primary significance for the current study.
- **Validation of the claim in the Study Design that Vaccine Issue is more Polarizing than AI Issue in Singapore:** Vaccine issue was more polarizing than AI; for AI, only 11% of respondents (pretest scores) were against the policy position that AI should be regulated, showing that it is not polarizing as vaccines where the distribution was more balanced (53% indicated general confidence, 16% in the midpoint, and 31% indicating general concern about vaccine risks).
  - **Further explanation for the rationale of RQ1-2-3 in the manuscript instead of hypothesizing these relationships:** While the substantive nature of our trainings led us to expect that motivated assessments can be reduced, extensive findings in the literature about the strength and persistence of motivated reasoning emerge as a countervailing consideration, leading us to not have a strong rationale for a directional hypothesis.
- **Examples for Message Construction:** For the poll stories, we carefully examined real-world and recent examples in Singapore and beyond for poll messages on the issues we focused on to use language similar to the coverage of the two issues (COVID-19 vaccine safety and regulation of AI technology)/
  - Belief in false information
    - https://www.straitstimes.com/singapore/around-one-in-four-singapore-residents-surveyed-believe-false-claim-about-covid-19-vaccine
  - AI regulation
    i. https://www.ipsos.com/en-sg/ai-making-singaporeans-and-most-asian-markets-nervous-about-job-security
    ii. https://sg.news.yahoo.com/63-surveyed-americans-want-government-152851731.html

- Example of social media misinformation poll that is referenced in the manuscript (not part of manipulation stories)
  - https://kirschsubstack.com/

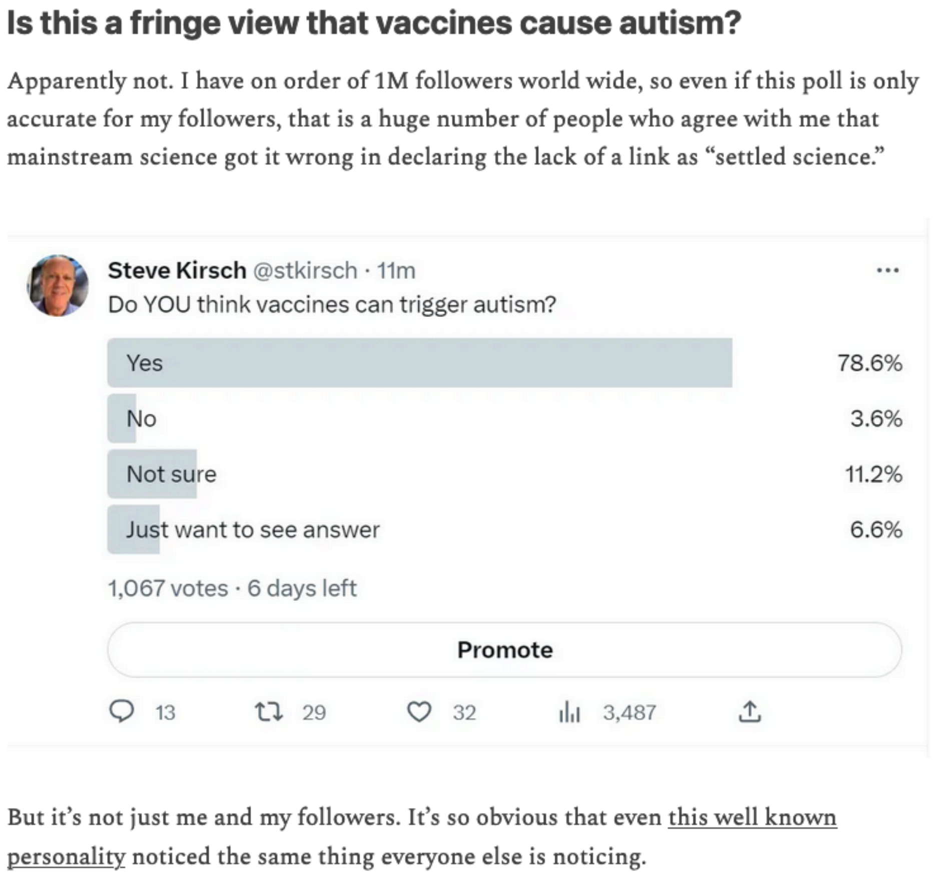

Is this a fringe view that vaccines cause autism?

Apparently not. I have on order of 1M followers world wide, so even if this poll is only accurate for my followers, that is a huge number of people who agree with me that mainstream science got it wrong in declaring the lack of a link as "settled science."

But it's not just me and my followers. It's so obvious that even this well known personality noticed the same thing everyone else is noticing.

- **General Definition to All Respondents at the Start of Study:** At beginning of W1, respondents were first given a definition of surveys and an example to ensure what our pre-test questions meant.
  "Please read:
  What is a survey / poll ? When covering issues and news, media organizations frequently refer to surveys and public opinion or election polls.

A survey / poll is a report on a systematic and scientific analysis of public opinion. It provides information about public's preferences such as voting intentions in an upcoming election, views about a particular policy position, beliefs about issues or support for political leaders etc. It may be on any topic, such as politics, health, economy, and education etc.
Example survey / poll finding: "A poll finds that majority of the public (57%) is optimistic about the economy in the next year."

- **Why not measure polling knowledge as a pre-test moderator, like education, scientific literacy, and perceived numeracy?** Given that survey methods training is our manipulations, we did not measure pre-test knowledge/literacy about survey methods to avoid priming and sensitizing respondents (i.e. demand effects) and interfering with the control condition participants' engagement (contamination with experimental effects) with polls viewed at W2. Measuring general science literacy – as we did – instead of existing knowledge of poll methodology before trainings avoids priming and demands effects, for both the control and the training conditions.

E. Power Analysis, Sample Size Considerations, and Additional Sampling Details [click here to return to TOC]

Power Analysis and Sample Size Considerations:

1. Social science interventions, most frequently against misinformation research recently, have effect sizes that vary a lot across contexts, ranging from small to moderate (Chan et al., 2017; de Saint Laurent et al., 2022).
2. We have three different interventions in this study: passive literacy, active literacy, and inoculation, and these three interventions are hypothesized to have increasing levels of effect as follows: passive literacy < active literacy < inoculation. They are conceptualized and designed to be additive, such that active literacy is composed of passive literacy and an additional component of the quiz, while inoculation is composed of active literacy an additional component of forewarning.
3. Recent inoculation research suggested and used an effect size of d=.20 (Roozenbeek et al., 2022), yet considering that inoculation is hypothesized to have the strongest effect while the two other interventions are presumed to be weaker, a potentially smaller overall effect size of d=.15 range is also taken into consideration as a more conservative approach. We do not test d=.10 because our training interventions are substantive; for example, active literacy and inoculation interventions include a detailed quiz + correct answer explanations session. The two dominate the overall effects more than the passive literacy intervention alone which does not have this more substantive component.
4. Taking into account that there are (4 X 8) = 32 conditions arising from both waves, the expected sample size for W2 is taken into account (with the expectation given by the survey company for the recontact rate, which is between 50% to 60%).
5. Gpower is used; holding 32 comparison groups, an alpha level of p= .05 and a power of .80, numerator df= 21 ([4-1]*[8-1]) constant, an effect size of
    a. d=.20 requires N=551 at minimum
    b. d=.15 requires N=966 at minimum

6. These values were considered in budgeting for the W1 target sample size with the survey company (for a W2 N=1000 approximately expected).
7. We conclude that the study is well-powered for the main analysis as we achieved N=1076 well over the minimum limits of N=551 and N=966 mentioned above.
8. However, for the models/analyses that predict public opinion outcomes (e.g. perceived public opinion), where we analyze vaccine and AI-related issues separately, we expect the power to be weaker given issue-specific analyses if we take into account d=.15. However, for d=.20, N=551 X 2 = 1102 is still very close to our achieved sample size of N=1076.
9. While for three-way interventions (moderation effects by pre-existing individual differences), one might also be concerned about power, the fact that we found significant effects for most of these models is important. Overall, we conclude the study had adequate power.
10. However, please see "deviations from preregistration" elsewhere in this Supplementary Materials based on reviewer feedback during revisions that raised issue with statistical power for the public opinion issue perceptions (which are now removed from main paper and tested as exploratory findings).

Additional Sampling Details:

1. *Blendi & respondi* follows ICC/ESOMAR Codes for its regulations. It includes 300+ criteria for quotas and segmentation in the entire panel and conducts random selection of respondents within quotas for specific surveys. Inattentive respondents such as speeders and nonsense response providers to open ended questions are actively identified and sanctioned if there are repeated violations. For compensation, participants receive points that are "redeemable in a gift catalogue, into cash rewards or, alternatively, they may donate the amount to a selected organization (Blendi & respondi, 2025). This panel is invite-only recruitment (email and human verification), does not involve river sampling
2. The initial sample size for Wave-1 (W1) was N=2,062 and for Wave-2 (W2, recontact) was N=1,278. Hence, the panel retention rate from W1 to W2 was at 62%.
   a. These sample sizes already account for (exclude) the 9 respondents in W2 whose match ID number did not match any ID at W1 due to the survey company's small technical error (N_{W1}=2,071 and N_{W2}=1,285). This is a negligible recruitment issue and does not induce any bias.
3. **Speeders, laggards, and consent withdrawals:** Post-data collection procedures detected a few low-quality responses that we removed: only 8 speeders at W1 (who completed the survey in under 7 minutes) and only 3 laggards at W2 (who took more than 20 minutes spent on the poll exposure page) were removed. Taking the survey under 7 minutes is extreme speeding as W1 involved substantive training sessions as manipulations (which had minimum page times) and indicates not all content was read. There were no speeders for W2 because it is a much shorter survey, however, a few extreme laggards on the message exposure page were removed because they did not complete the survey in one sitting, which might interfere with message evaluation (outcome measures). Only 24 respondents (22 at W1

and 2 at W2) requested withdrawal of their responses after debriefing. Hence, the effective sample size for W1 is N= 2,027, and the main longitudinal analysis sample size is N=1,076.

4. **Attention checks:** To ensure attentive respondents during data collection, two attention checks were employed at W1 (Shamon & Berning, 2020). No participants were removed in W2 for failure in attention check since these were already attentive and successful completers of W1, but they were warned with an attention boost question. Full wording:

Wave 1

WAVE 1 Attention Check Question 1: This is a question to check your attention. You must select the response option that is in the middle. It is the fourth response option. It is the one between "Slightly agree" and "Slightly disagree".

Completely agree (1)
Somewhat agree (2)
Slightly agree (3)
Neither agree nor disagree (4)
Slightly disagree (5)
Somewhat disagree (6)
Completely disagree (7)

WAVE 1 Attention Check Question 2: You must select the second response option from the top. This is a question to check your attention. You must select the second option; it is the one between Completely agree and Slightly agree.

Completely agree (1)
Somewhat agree (2)
Slightly agree (3)
Neither agree nor disagree (4)
Slightly disagree (5)
Somewhat disagree (6)
Completely disagree (7)

Wave 2

WAVE 2 Attention Boost Question 1: This is a question to check your attention. You must select the response option that is in the middle. It is the fourth response option. It is the one between "Slightly agree" and "Slightly disagree".

Completely agree (1)
Somewhat agree (2)
Slightly agree (3)
Neither agree nor disagree (4)
Slightly disagree (5)
Somewhat disagree (6)
Completely disagree (7)
[if incorrectly answered, the prompt enforced respondents to try again to answer correctly: “Please read carefully and fully, and respond accordingly to be able to continue and complete the survey.”]

WAVE 2 Attention Boost Question 2: What number do you get if you add 1 and 2? In other words, what does 1 + 2 equal to? Write the correct answer as a number.
[if incorrectly answered, the prompt enforced respondents to try again to answer correctly: e.g. “The value must be less than or equal to 3.” if they wrote a value higher than 3.]

WAVE 2 Attention Boost Question 3: This is a question to check your attention. You must select "Always" in order to be able to continue the survey.
Never (1)
Rarely (2)
Sometimes (3)
Usually (4)
Always (5)
[if incorrectly answered, the prompt enforced respondents to try again to answer correctly: “Please read carefully and fully, and respond accordingly to be able to continue and complete the survey.”]

5. **Sample is slightly over-educated than the general public. Can this be a serious concern for the biasing of our findings? No.** The fact that education levels did not moderate our most notable finding (inoculation effect) while moderating the passive literacy intervention effects is notable, because 1) education moderated at least one intervention effect, and 2) this heterogeneity in moderation findings across different interventions suggests that variance in our respondents’ education levels was substantive enough to result in these diverse effects. This lessens the concerns about the over-representation of high-education respondents as any systematic pattern (bias) in findings seems unlikely.

F. All Manipulations and Messages [click here to return to TOC]

Wave 1 Conditions

WAVE 1: CONTROL CONDITION

SLIDE-1

We are interested in your attitudes towards baseball in this section.
Please read carefully and fully.

SLIDE-2

c1_1
Baseball Rules
"Baseball is a sport that dates back as far as 1744 and formats of the game have been in place until the modern era today. The game is predominantly big in North America, Canada and Japan. The game is played worldwide with the pinnacle of sport coming from the World Series of Baseball. Ironically, this event is only competed by North American teams."

SLIDE-3

c1_2
Objective of the Game

"The objective of baseball is to score more runs than your opponent. The idea is to hit the ball thrown at you as far as you can before running around 4 bases to complete a run. Once a player manages to get around the four bases before being tagged out, then another batter steps in."

SLIDE-4

c1_3
Players & Equipment - Part 1

"A game is played out between two teams, each made up of 9 players. The game lasts for 9 innings with each team alternating between batting and fielding in each inning. The scores at the end of the innings are added to a cumulative score and the team with most points wins. Each team has three outs per inning before they then swap roles. Each inning can be broken down into the top (where the away team bats), and the bottom (where the home team bats). The field is split into two sections: infield and outfield. Separating the infield and outfield is a diamond shape with four bases, spaced at 90 feet apart each. In the center of the infield is the pitching mound where the pitcher stands and throws the ball toward the batter. The batter stands at the home plate. The other three bases are known as first base, second base and third base. The batter must touch all bases before successfully scoring a run."

SLIDE-5

c1_4
Players & Equipment - Part 2

"The bats are made out of either wood, aluminium or metal materials. The ball is white with red stitching and is roughly 3 inches in diameter. The fielding team wear ‘mits’, which are basically an oversized glove to help them catch and pick up the ball. The catcher (standing behind the batter to catch any balls missed) wears extra padding in their glove, along with leg guards, a body pad, and a helmet.

Scoring: To score, a batter must hit the ball with the bat into the designated fielding area and make it around all four bases (before the fielding team is able to collect the ball and throw it to the base the batter is running to). A player can score a mandatory point if they hit a home run, which usually means the ball has left the playing area, often landing in the crowd. A player can stop at any base if they feel they might not make it to the next base before being tagged out."

SLIDE-6

c1_5
Players & Equipment - Part 3

"Players can score multiple points from one hit if more than one player is already on one of the bases. When you hear the phrase 'the bases are loaded', this refers to the instance where there is a player on every base. So, every time a batter successfully makes it to first base, the other

players on the second and third bases are able to trickle home, earning a point for their team each time. Depending on how many players get around to home plate before being tagged will depend on how many points you score."

SLIDE-7

c1_6
Players & Equipment - Part 4

"If the batter manages to hit the ball from the pitcher, they must make an effort to at least get to first base. They can then run to as many bases as they wish before being tagged out. Each base must be touched with some part of the batters body when running past.

A batter gets up to three strikes before getting out. A strike is deemed when a batter swings for a ball and misses it. The batter can leave the ball but, if it's within a certain area (called the 'strike zone'), then a strike will also be given. If four balls miss the strike zone and the batter does not swing their bat, they can walk to first base. When on base, the batter can run to the next base at any point. "

SLIDE-8

c1_7
Winning the Game

"To win a game, you must outscore your opposition through the 9 innings played. The team with the most points after 9 innings is deemed the

winner. In the event of a tie, extra innings are played until a winner has been concluded." (Reference: <www.rulesofsport.com>)

SLIDE-8

c1_baseballq1 We are interested in your attitudes towards baseball in this section. How likely are you to play baseball in future?

- ○ Not at all likely (1)
- ○ A little likely (2)
- ○ Somewhat likely (3)
- ○ Very likely (4)
- ○ Extremely likely (5)

SLIDE-9

c1_baseballq2 How entertaining do you find baseball?

- ○ Not at all entertaining (1)
- ○ A little entertaining (2)
- ○ Somewhat entertaining (3)
- ○ Very entertaining (4)
- ○ Extremely entertaining (5)

WAVE 1: PASSIVE LITERACY CONDITION

SLIDE-1

Q314 **Please read carefully and fully.**

c2_1
Methodological quality of surveys:

While there are many surveys (polls) we encounter and read in media, surveys differ in their level of methodological quality (scientific strength).

Some surveys are methodologically strong (**high quality**) while others are poor (**low quality**).

High quality surveys provide more reliable and accurate statistical findings about the society while low quality surveys may be very misleading.

There are a few key details to examine to evaluate the methodological quality of surveys. These details are ideally provided together with survey results when they are presented in the media. **We should pay attention to these details while reading survey reports.**

SLIDE-2

c2_2 **Population vs. Sample:**

Population is the collective of individuals a survey attempts to understand. Depending on the scope and aim of a survey, it could be an entire country or retired individuals, for example.

Sample is the collective of individuals who participate in a survey and are drawn from the population under study. A survey tries to understand a population by studying a sample of it (which is a very small segment of a population).

This is similar to tasting a spoon of soup while we are cooking a pot of soup. Soup in spoon would be the sample and the soup in the pot is the population.

SLIDE-3

c2_3 **Representativeness of a sample:**

Representativeness refers to how well **the sample** of a survey captures **the population** under study.

Some samples are representative. Representative samples are collected with **probability-based scientific random selection methods**, which gives every participant equal chance of being included. Probability-based, randomly selected samples capture the diversity in a population successfully and usually avoid bias.

On the other hand, **non-probability based samples, such as convenience samples** of participants who are included in a survey without random selection, are **NOT** representative. Some types of individuals (for example, those who happened to be in a shopping mall where survey was conducted) may be over-represented in a convenience sample, creating a bias.

Going with the soup example, in order to taste the soup more accurately while cooking, we usually mix the soup and sample a representative sample of it with the spoon. If we do not mix the soup first and only sample from the top of the pot, our spoon will not take a representative sample of the soup.

SLIDE-4

c2_4
Sample size of a survey:

Sample size refers to the number of individuals who participated in a survey. Generally, greater number of participants (**larger sample size**) provides more statistical reliability and confidence in the statistical findings.

For example, it is very hard to provide reliable results for a country based on only 100 participants in a survey, because such a low sample size may not include any person with a particular demographic feature (for example, a female above 75 years old who has college degree education

level). A sample of 1000 individuals at least is much better than, say, 100 individuals.

SLIDE-5

c2_5 **Response rate in a survey:**

When participants are invited to surveys, not everyone accepts the invitation. The ratio between invitations and completed surveys refers to the **response rate**. If response rates are too low, it might sometimes lead to a bias in the representativeness of a sample. A response rate of 35% is much better than a response rate of 5%.

Also, if response rates are small and different for different types of individuals, it might also create a bias in survey results. For example, participants more interested in politics can have higher response rate in a political survey and this may bias the results (people with greater political interest being overly-represented in that survey).

SLIDE-6

c2_6 **Precision of results: margin of error**

Margin of error refers to variability in results of a statistical finding. Survey results do not give exact findings, but provide estimates within a possibility range. This range is determined by the size of margin of error.

For example, **+/- 3% margin of error means** that a particular finding is within 3 points below and 3 points above the exact number. A finding that states 54% (with +/-3% margin of error) of the public supports a policy means that the exact public support level is highly likely somewhere within the range of 51% (54-3) to 57% (54+3).

A smaller margin of error is a stronger methodological quality indicator because the survey finding is more precise, the finding is within a narrower range. A margin of error of 3% is much better than a margin of error of 6%.

SLIDE-7

c2_7
Summary

1. Surveys that have **representative (probability-based) samples**
2. Surveys that have **larger sample size**
3. Surveys that have **higher response rate**
4. Surveys that have **lower margin of error**

tend to <u>have stronger methodological quality and are more scientific, accurate, reliable</u> than those surveys which do not have these properties.

WAVE 1: ACTIVE LITERACY CONDITION

(PASSIVE LITERACY CONDITION'S ALL CONTENT + QUIZ AND CORRECT ANSWER EXPLANATION INTERACTIVE SESSION)

SLIDES-1 TO 7: PASSIVE LITERACY CONTENT

SLIDE-8

Q394 **Let's exercise! In this section we have 6 quiz questions, please respond by making your best attempt.**

SLIDE-9

c3_q01 Which one is a methodologically stronger / more scientific survey?

○ A survey with a convenience sample (1)

○ A survey with probability-based random sampling (2)

○ Not sure (5)

SLIDE-10
Q587 Correct answer: A probability-based random sampling survey has stronger methodological quality than a convenience sample survey.

SLIDE-11

c3_q02 Which one is a methodologically stronger / more scientific survey?

- ○ A survey with 100 respondents (1)
- ○ A survey with 1000 respondents (2)
- ○ Not sure (3)

SLIDE-12

Q590 Correct answer: A survey with 1000 respondents has stronger methodological quality than a survey with only 100 respondents.

SLIDE-13

c3_q03 Which one is a methodologically stronger / more scientific survey?

- ○ A survey with a response rate of 3% (1)
- ○ A survey with a response rate of 35% (2)
- ○ Not sure (3)

Q592 Correct answer: A survey with 35% response rate has stronger methodological quality than a survey with only 3% response rate.

SLIDE-14

c3_q1 Assuming all other characteristics are the same, which of the following surveys' sample characteristics would provide the most accurate and reliable statistic for a country?

- ◯ 1-) A convenience sample of individuals in a shopping mall (1)
- ◯ 2-) A non-probability based sample of students at schools (4)
- ◯ 3-) A non-probability based sample of people from each district (5)
- ◯ 4-) A probability-based nationally representative sample of all individuals in the country (6)

SLIDE-15

Q396
The correct answer is Option 4: "A probability-based nationally representative sample of all individuals in the country." This describes a representative sample for the population (country).

SLIDE-16

c3_q2 Assuming all other characteristics are the same, which of the following surveys' sample characteristics would provide the best estimate of support levels in the country for a particular policy position?

- 1-) When the sample size of the poll is 5,000, half of it coming from cities and the other half of it coming from rural areas (1)
- 2-) When the sample size of the poll is 10,000, and is representative of 3 big cities or districts (4)
- 3-) When the sample size of the poll is 8,000, and it is a convenience non-probability sample (5)
- 4-) When the sample size of the poll is 5,000, and it is a probability-based nationally representative sample (6)

SLIDE-17

Q398 The correct answer is Option 4: "When the sample size of the poll is 5,000, and it is a probability-based nationally representative sample" This describes a nationally representative sample. While Option 2 (10,000) has higher sample size than Option 4 (5,000), sample representativeness (probability based sample in Option 4) is a more important factor for methodological quality than sample size alone. Option 4 has also a large enough sample to have representative and reliable statistical findings.

SLIDE-18

c3_q3 Assuming all other characteristics are the same, which of the following survey results would you be most confident represents a policy position that is supported by a majority of people (more than half - 50% - of the population)?

- 1-) Policy position got 47% support, with a 2% margin of error (1)
- 2-) Policy position got 51 % support, with a 2% margin of error (4)
- 3-) Policy position got 54% support, with a 2% margin of error (5)
- 4-) Both 2nd and 3rd options show that position is being supported by the majority (more than 50%) (6)

SLIDE-19

Q400 The correct answer is Option 3, because it shows that 54% result is within range of 52% (54-2) and 56% (54+2), the range of 52 to 56 is clearly more than 50% threshold, showing the majority support. On the other hand, Option 2, 51%, range from 49% to 53%, so we are not confident if the survey in Option 2 represents a majority position for the policy position.

WAVE 1: INOCULATION CONDITION

(ACTIVE LITERACY CONDITION'S ALL CONTENT + FOREWARNING COMPONENT)

SLIDES-1 TO 7: PASSIVE LITERACY CONTENT

SLIDES-8 TO 19: INTERACTIVE AND EXPLANATORY QUIZ SESSION

SLIDE-20

c4_forewarn1 **IMPORTANT: Misuse of Surveys as Misinformation !**

Surveys/polls may also be used as misinformation by some people and organizations.

SLIDE-21

c4_forewarn2 **IMPORTANT: Misuse of Surveys as Misinformation !**

> Sometimes survey **results may be mis-contextualized** in order to use them as evidence in support of an inaccurate claim.

SLIDE-22

c4_forewarn3 **IMPORTANT: Misuse of Surveys as Misinformation !**

> Surveys with **poor methodological quality and important scientific limitations** may be presented as evidence for accurate public opinion.

SLIDE-23

c4_forewarn4 **IMPORTANT: Misuse of Surveys as Misinformation !**

> Some digital platforms have a survey/poll design feature, such as **Twitter Polls and Instagram Polls. These are not scientific and have extremely low methodological quality.** They are considered **'straw polls.'** These surveys may be engaging and fun, but they are not scientific.

SLIDE-24

c4_forewarn5 **IMPORTANT: Misuse of Surveys as Misinformation !**

For these reasons, we should be critical and careful while reading survey findings. We should check the methodological details and other relevant information to ensure the credibility and quality of evidence presented to protect ourselves from misinformation that makes use of surveys.

Wave 2 Conditions

Condition 1 (Low quality, Majority Concerned, Vaccine)

c1_m **The majority (58%) of the public is CONCERNED about the safety of COVID-19 vaccines**

According a recent survey released by KnowledgeMetrics, the majority of the public is concerned about the side effects of the COVID-19 vaccines. 58% of the respondents in the survey stated that they believe the serious side effects of the vaccine, such as having hearth problems, hospitalization and even death, are very common in the population. This shows that most of the public is very worried about the safety of COVID-19 vaccines.

The survey was conducted with a convenience sample of online news consumers of the major digital news platforms in October 2023, and the response rate was 7%. The sample size of the survey was 289 adults (21 years old and older). The margin of error for this result is +/- 7 percentage points. The participants were recruited through invitations in the websites and social media accounts of digital news channels and answered the questions through an online form.

Condition 2 (High quality, Majority Concerned, Vaccine)

c2_m **The majority (58%) of the public is CONCERNED about the safety of COVID-19 vaccines**

According a recent survey released by KnowledgeMetrics, the majority of the public is concerned about the side effects of the COVID-19 vaccines. 58% of the respondents in the survey stated that they believe the serious side effects of the vaccine, such as having hearth problems, hospitalization and even death, are very common in the population. This shows that most of the public is very worried about the safety of COVID-19 vaccines.

The survey was conducted on a nationally-representative probability-based sample in October 2023, and the response rate was 70%. The sample size of the survey was 1537 adults (21 years old and older). The margin of error for this result is +/- 2 percentage points. The participants were recruited through address-based sampling and were interviewed either face to face or through telephones with human interviewers.

Condition 3 (Low quality, Majority Confident, Vaccine)

c3_m **The majority (58%) of the public is CONFIDENT about the safety of COVID-19 vaccines**

According a recent survey released by KnowledgeMetrics, the majority of the public is confident about the safety of the COVID-19 vaccines. 58% of the respondents in the survey stated that they believe the serious side effects of the vaccine, such as heart problems, hospitalization and death, are extremely rare in the population. This shows that most of the public is confident about the safety of COVID-19 vaccines.

The survey was conducted with a convenience sample of online news consumers of the major digital news platforms in October 2023, and the response rate was 7%. The sample size of the survey was 289 adults (21 years old and older). The margin of error for this result is +/- 7 percentage points. The participants were recruited through invitations in the websites and social media accounts of digital news channels and answered the questions through an online form.

Condition 4 (High quality, Majority Confident, Vaccine)

c4_m **The majority (58%) of the public is CONFIDENT about the safety of COVID-19 vaccines**

According a recent survey released by KnowledgeMetrics, the majority of the public is confident about the safety of the COVID-19 vaccines. 58% of the respondents in the survey stated that they believe the serious side effects of the vaccine, such as heart problems, hospitalization and death, are extremely rare in the population. This shows that most of the public is confident about the safety of COVID-19 vaccines.

The survey was conducted on a nationally-representative probability-based sample in October 2023, and the response rate was 70%. The sample size of the survey was 1537 adults (21 years old and older). The margin of error for this result is +/- 2 percentage points. The participants were recruited through address-based sampling and were interviewed either face to face or through telephones with human interviewers.

Condition 5 (Low quality, Majority Support, AI)

c5_m **The majority (58%) of public SUPPORTS regulation of artificial intelligence (AI) technologies in work, school, and healthcare settings**

According a recent survey released by KnowledgeMetrics, the majority of the public supports stricter regulation of artificial intelligence (AI) technologies. 58% of the respondents in the survey stated that they believe it is very important to regulate and control how AI technologies are integrated into workplace, school and healthcare settings. Respondents highlighted that unregulated AI use could lead to problems like job losses, decline in student performance, and breach of patient privacy; therefore AI should be regulated more strictly. This shows that most of the public supports stronger regulation of AI use.

The survey was conducted with a convenience sample of online news consumers of the major digital news platforms in October 2023, and the response rate was 7%. The sample size of the survey was 289 adults (21 years old and older). The margin of error for this result is +/- 7 percentage points. The participants were recruited through invitations in the websites and social media accounts of digital news channels and answered the questions through an online form.

Condition 6 (High quality, Majority Support, AI)

c6_m **The majority (58%) of public SUPPORTS regulation of artificial intelligence (AI) technologies in work, school, and healthcare settings**

According a recent survey released by KnowledgeMetrics, the majority of the public supports stricter regulation of artificial intelligence (AI) technologies. 58% of the respondents in the survey stated that they believe it is very important to regulate and control how AI technologies are integrated into workplace, school and healthcare settings. Respondents highlighted that unregulated AI use could lead to problems like job losses, decline in student performance, and breach of patient privacy; therefore AI should be regulated more strictly. This shows that most of the public supports stronger regulation of AI use.

The survey was conducted on a nationally-representative probability-based sample in October 2023, and the response rate was 70%. The sample size of the survey was 1537 adults (21 years old and older). The margin of error for this result is +/- 2 percentage points. The participants were recruited through address-based sampling and were interviewed either face to face or through telephones with human interviewers.

Condition 7 (Low quality, Majority Oppose, AI)

c7_m **The majority (58%) of public OPPOSES regulation of artificial intelligence (AI) technologies in work, school, and healthcare settings**

According a recent survey released by KnowledgeMetrics, the majority of the public opposes stricter regulation of artificial intelligence (AI) technologies. 58% of the respondents in the survey stated that they believe it is not needed to regulate and control how AI technologies are integrated into workplace, school and healthcare settings. Respondents highlighted that more regulation is not needed as job losses, decline in student performance, and breach of patient privacy were minimal and benefits of AI should be maximized without restrictions. This shows that most of the public opposes stronger regulation of AI use.

The survey was conducted with a convenience sample of online news consumers of the major digital news platforms in October 2023, and the response rate was 7%. The sample size of the survey was 289 adults (21 years old and older). The margin of error for this result is +/- 7 percentage points. The participants were recruited through invitations in the websites and social media accounts of digital news channels and answered the questions through an online form.

Condition 8 (High quality, Majority Oppose, AI)

c8_m **The majority (58%) of public OPPOSES regulation of artificial intelligence (AI) technologies in work, school, and healthcare settings**

According a recent survey released by KnowledgeMetrics, the majority of the public opposes stricter regulation of artificial intelligence (AI)

technologies. 58% of the respondents in the survey stated that they believe it is not needed to regulate and control how AI technologies are integrated into workplace, school and healthcare settings. Respondents highlighted that more regulation is not needed as job losses, decline in student performance, and breach of patient privacy were minimal and benefits of AI should be maximized without restrictions. This shows that most of the public opposes stronger regulation of AI use.

The survey was conducted on a nationally-representative probability-based sample in October 2023, and the response rate was 70%. The sample size of the survey was 1537 adults (21 years old and older). The margin of error for this result is +/- 2 percentage points. The participants were recruited through address-based sampling and were interviewed either face to face or through telephones with human interviewers.

G. Other Measures and Full Details [click here to return to TOC]

WAVE 1

AI Issue
How risky do you think the use of artificial intelligence (AI) technologies in settings such as work, school, and healthcare is?
How concerned are you about the use of artificial intelligence (AI) technologies in settings such as work, school, and healthcare?
How worried are you about the use of artificial intelligence (AI) technologies in settings such as work, school, and healthcare?

Now please focus on what other people think about the use of artificial intelligence (AI) in our lives. How risky do you think most people in the country find the artificial intelligence (AI) technologies? How concerned do you think most people in the country are about artificial intelligence (AI) technologies? How worried do you think most people in the country are about artificial intelligence (AI) technologies?

What portion of the general public in the country do you think is concerned about artificial intelligence (AI) technologies?

“Would you support or oppose a policy proposal that aims to regulate more strictly the use of artificial intelligence (AI) technologies in settings such as work, school, and healthcare?” with seven response options ranging from “Strongly oppose regulation” to “Strongly support regulation.”

“Overall, how positive or negative do you feel towards artificial intelligence (AI) technologies?” with seven response options ranging from “Strongly negative” to “Strongly positive.”

Digital News Literacy Behaviors
When you are reading digital news articles on the Internet, how often do you...

1. Cross-check with mass media sources like news agencies, newspapers, television
2. Check different social media platforms regarding the information
3. Cross check the information with online verification sites
4. Read comments on the article to see if there is discussion about the veracity of information
5. Check the sources cited/references in the story
6. Examine the numerical and statistical information in detail
7. Examine the publication date of the news story
8. If provided, read the updates and changes to the news story
9. Zoom into images and photos to examine if they are edited / photoshopped

Never
Rarely
Sometimes
Frequently
Very frequently

Subjective Numeracy

1. How good are you at working with fractions?
2. How good are you at working with percentages?
3. How good are you at calculating a 15% tip?
4. How good are you at figuring out how much a shirt will cost if it is 25% off?

Not at all good
A little good
Somewhat good
Very good
Extremely good

5. When reading the newspaper, how helpful do you find tables and graphs that are parts of a story?

Not at all helpful
A little helpful
Somewhat helpful
Very helpful
Extremely helpful

6. When people tell you the chance of something happening, do you prefer that they use words ("it rarely happens") or numbers ("there's a 1% chance")?

Always prefer words
Usually prefer words
Prefer words and numbers about equally
Usually prefer numbers
Always prefer numbers

7. When you hear a weather forecast, do you prefer predictions using words (e.g., “there is a small chance of rain today”) or predictions using percentages (e.g., “there will be a 20% chance of rain today”)?

Always prefer words
Usually prefer words
Prefer words and percentages about equally
Usually prefer percentages
Always prefer percentages

8. How often do you find numerical information to be useful?

Never
Rarely
Sometimes
Frequently
Very frequently

Science Literacy (Adopted from Everyday Scientific Reasoning Scale)
Introduction: Alex is overweight and wants to find a research-proven method to lose weight. In the next page, we will present 8 short questions about different aspects of Alex's search for an effective diet. For each question please answer: True or False. Please provide your best guess if you are not sure.

1. sl1 Alex joins a clinical trial testing a new diet pill. Half of the participants receive the pill and half receive a placebo. Both Alex’s doctor and the researchers know which group Alex belongs to, but Alex himself does not know. This situation is sufficient to examine the effectiveness of the new pill. True or False? [accurate response: False]
2. sl2 It has been proven that people who have a diet rich in cucumbers weigh 20% less than people who don’t eat cucumbers at all. Therefore, Alex can conclude that eating cucumbers is helpful for weight loss. True or False? [accurate response: False]
3. sl3 As part of Alex's attempts to lose weight, he decides to stop eating in between meals and to run on the beach. A week later he finds out he lost 5 kg. Alex can determine with certainty the cause of his weight loss. True or False? [accurate response: False]

4. sl4 Alex has been on a diet for a month and in support of this process visits a dietician to check whether he has lost weight. The dietician suggests that Alex test the activity of his digestive system. Healthy and proper digestive system activity can serve as evidence to weight loss. True or False? [accurate response: False]
5. sl5 Alex read an article about a study regarding candy consumption. In the study, those who managed to resist the temptation and not eat the candy – won a weekend in a beach resort. Alex can conclude that if this method worked in the study, he could also use it in order to stop eating candies. True or False? [accurate response: False]
6. sl6 An ad agency wants to examine the effectiveness of their six-month campaign promoting a low protein diet. For this purpose, they administrate surveys before and after the campaign. During this time (and with no connection to the campaign), the media reported that the winner of a famous reality music competition had lost weight following a low protein diet. The survey at the end of the campaign showed an increase in awareness about low protein diets. Alex can conclude that the campaign increased the awareness about this diet. True or False? [accurate response: False]
7. sl7 Alex joins a weight loss support group which explores the effectiveness of two different dieting methods. All participants who weigh over 120 kg try method A and all participants under 120 kg try method B. This way the group can tell which method is more effective for losing weight. True or False? [accurate response: False]
8. sl8 Alex buys a new digital scale. First thing every morning he weighs himself wearing his pajamas, and each time he sees different results. The only reason to explain the different results, is that Alex gains and losses weight every day. True or False? [accurate response: False]

Coding for Education Levels: Respondents were asked, "What is the highest level of school you have completed or the highest degree you have received?" with options "Primary school or less," "Some high school but no diploma," "High school graduate," "Some college but no degree," "Polytechnic," "Bachelor's degree in college (4-year)," "Master's degree," "Doctoral degree," "Professional degree." Taking into account the levels and types of schooling (e.g. polytechnic vs Bachelor's programs), we created four levels of education as follows 1) high school graduate or less, 2) some college or polytechnic, 3) Bachelor's graduate, and 4) graduate or professional degrees. Scores range from 0 (lowest level) to 1 (highest level).

Age How old are you? Please write in numbers.

Sex Are you:
o Male (1)
o Female (2)
o Other (3)

RaceSG What is your ethnicity?
o Chinese (1)
o Malay (2)
o Indian (3)
o Other (4)

EducationSG What is the highest level of school you have completed or the highest degree you have received?
o Primary school or less (1)
o Some high school but no diploma (2)
o High school graduate (3)
o Some college but no degree (4)
o Polytechnic (5)
o Bachelor's degree in college (4-year) (6)
o Master's degree (7)
o Doctoral degree (8)
o Professional degree (9)

Income Last year -- that is, in 2022 -- what was the average MONTHLY income of all the people living in your house or apartment BEFORE taxes in Singaporean Dollars (SGD)?
o $1000 or less (1)
o $1001 to $3000 (2)
o $3001 to $5000 (3)
o $5001 to $7000 (4)
o $7001 to $9000 (5)
o $9001 to $11000 (6)
o $11001 to $13000 (7)
o $13001 to $15000 (8)
o $15001 to $17000 (9)
o $17001 to $20000 (10)
o $20001 to $25000 (11)
o More than $25000 (12)
o Do not want to respond (14**) [N=51 respondents who chose these were imputed the median value of 5]**

interest1 How interested are you in reading survey / poll findings?
o Not at all interested (1)
o A little interested (2)
o Somewhat interested (3)
o Very interested (4)
o Extremely interested (5)

interest2 How frequently do you read survey / poll findings?

o Never (1)
o Rarely (2)
o Sometimes (3)
o Frequently (4)
o Very frequently (5)

interest3 How much attention do you give to survey / poll findings that are mentioned in news articles or in other media?

o No attention at all (1)
o A little attention (2)
o Some attention (3)
o Considerable attention (4)
o A lot of attention (5)

pre_credible How credible do you find survey/poll findings in the media in general?

o Not at all credible (1)
o A little credible (2)
o Somewhat credible (3)
o Very credible (4)
o Extremely credible (5)

pre_concern1 How risky is the possibility that surveys/polls can manipulate and mislead people?
o Not at all risky (1)
o A little risky (2)
o Somewhat risky (3)
o Very risky (4)
o Extremely risky (5)

pre_efficacy1 How confident are you in understanding survey/poll findings in the media?

o Not at all confident (1)
o A little confident (2)
o Somewhat confident (3)
o Very confident (4)
o Extremely confident (5)

W1 post test manipulation check

efficacy1 How confident are you in understanding survey/poll findings in the media?

o Not at all confident (1)
o A little confident (2)
o Somewhat confident (3)
o Very confident (4)
o Extremely confident (5)

efficacy2 How much informed are you in understanding survey/poll findings in the media?

o Not at all informed (1)
o A little informed (2)
o Somewhat informed (3)
o Very informed (4)
o Extremely informed (5)

mc_for_control How informed do you feel about the game of baseball?

- o Not at all informed (1)
- o A little informed (2)
- o Somewhat informed (3)
- o Very informed (4)
- o Extremely informed (5)

Additional Controls for Appendix Models

vaccination Did you get a COVID-19 vaccine?
- o No (1)
- o Yes, 1 dose (2)
- o Yes, 2 doses (3)
- o Yes, 3 doses (4)
- o Yes, 4 doses or more (5)

sideeffects Did you yourself experience any side effects from any of the COVID-19 vaccines you took?
- o No side effects at all (1)
- o Very minor side effects (2)
- o Minor side effects (3)
- o Moderate side effects (4)
- o Major side effects (needed to go to hospital) (5)

experienceAI Have you ever used artificial intelligence (AI) chatbots such as ChatGPT?

- o Never (1)
- o Only once (2)
- o A few times (3)
- o Quite a few times (4)

o Using it regularly (5)

Ideology Generally speaking, would you describe your political views as:
o Very conservative (1)
o Somewhat conservative (2)
o Moderate; middle of the road (3)
o Somewhat liberal (4)
o Very liberal (5)
o Do not want to respond (6)

WAVE 2

Trust in Science (Hendriks et al., 2015; Ophir and Jamieson, 2021)

Thinking about scientists in general, to what extent would you say that scientists in general
share your values
are competent at what they do
are honest and trustworthy

Not at all true
A little true
Somewhat true
Very true
Extremely true

Thinking about science in general, to what extent would you say that...
Scientific theories are trustworthy
Scientific methods are reliable
Scientific findings and knowledge acquired from science are credible
Science is a trustworthy way to understand the world we live in

Not at all true
A little true
Somewhat true
Very true
Extremely true

Manipulation Checks

mc1 Please answer the following question about the story you just read carefully.
What was the survey / poll you just read about?
o COVID-19 vaccines (1)
o Artificial intelligence (2)
o Cryptocurrencies (3)

mc2a1 Please answer the following question about the story you just read carefully.
What was the survey / poll you just read about?
o COVID-19 vaccines (1)
o History museums (2)

mc2b1 Please answer the following question about the story you just read carefully.
What was the survey / poll you just read about?
o Artificial intelligence (1)
o History museums (2)

mc2xa1 Please answer the following question about the story you just read carefully.
In the survey / poll you just read, what was the finding?
o The majority (58%) of the public is CONCERNED about the safety of COVID-19 vaccines (1)
o The majority (58%) of the public is CONCERNED about cryptocurrencies (2)

mc2xa2 Please answer the following question about the story you just read carefully.
In the survey / poll you just read, what was the finding?
o The majority (58%) of the public is CONFIDENT about the safety of COVID-19 vaccines (1)

o The majority (58%) of the public is CONFIDENT about cryptocurrencies (2)

mc2xb1 Please answer the following question about the story you just read carefully.
In the survey / poll you just read, what was the finding?
o The majority (58%) of public SUPPORTS regulation of artificial intelligence (1)
o The majority (58%) of public SUPPORTS regulation of cryptocurrencies (2)

mc2xb2 Please answer the following question about the story you just read carefully.
In the survey / poll you just read, what was the finding?
o The majority (58%) of public OPPOSES regulation of artificial intelligence (1)
o The majority (58%) of public OPPOSES regulation of cryptocurrencies (2)

mc2a2 Please answer the following question about the story you just read carefully.
In the survey / poll you just read, what was the finding?
o The majority (58%) of the public is CONCERNED about the safety of COVID-19 vaccines (1)
o The majority (58%) of the public is CONFIDENT about the safety of COVID-19 vaccines (2)

mc2b2 Please answer the following question about the story you just read carefully.
In the survey / poll you just read, what was the finding?
o The majority (58%) of public SUPPORTS regulation of artificial intelligence (1)
o The majority (58%) of public OPPOSES regulation of artificial intelligence (2)

mc3_Representative How representative is the sample of the poll you just read?

o Not at all representative (1)
o A little representative (2)
o Somewhat representative (3)
o Very representative (4)
o Extremely representative (5)

c Assuming all other characteristics are the same, which of the following surveys' sample characteristics would provide the best estimate of support levels in the country for a particular policy position?

- o 1-) When the sample size of the poll is 5,000, half of it coming from cities and the other half of it coming from rural areas (1)
- o 2-) When the sample size of the poll is 10,000, and is representative of 3 big cities or districts (2)
- o 3-) When the sample size of the poll is 8,000, and it is a convenience non-probability sample (4)
- o 4-) When the sample size of the poll is 5,000, and it is a probability-based nationally representative sample (5)

d Which one is a methodologically stronger / more scientific survey?
- o A survey with a margin of error of 6 (1)
- o A survey with a margin of error of 2 (2)
- o Not sure (4)

a Which one is a methodologically stronger / more scientific survey?

- o A survey with a convenience sample (1)
- o A survey with probability-based random sampling (2)
- o Not sure (4)

mc6 What was the type of sample of the survey/poll you just read in the news story? Please write very briefly with a couple of words at most.

e How worried are you about potential misuse of and misinformation in surveys / polls?

- o Not at all worried (1)
- o A little worried (2)
- o Somewhat worried (3)
- o Very worried (4)
- o Extremely worried (5)

Q632 How informative do you find social media features such as Twitter Polls or Instagram Polls?
- o Not at all informative (1)
- o A little informative (2)
- o Somewhat informative (3)
- o Very informative (4)
- o Extremely informative (5)

credible How credible do you find survey/poll findings in the media in general?
o Not at all credible (1)
o A little credible (2)
o Somewhat credible (3)
o Very credible (4)
o Extremely credible (5)

accurate How acccurate do you find survey/poll findings in the media in general?
o Not at all accurate (1)
o A little accurate (2)
o Somewhat accurate (3)
o Very accurate (4)
o Extremely engaging (5)

informative How informative do you find survey/poll findings in the media in general?
o Not at all informative (1)
o A little informative (2)
o Somewhat informative (3)
o Very informative (4)
o Extremely informative (5)

concern1 How risky is the possibility that surveys/polls can manipulate and mislead people?
o Not at all risky (1)
o A little risky (2)
o Somewhat risky (3)
o Very risky (4)
o Extremely risky (5)

concern2 How worried are you that surveys/polls can manipulate and mislead people?
o Not at all worried (1)
o A little worried (2)

o Somewhat worried (3)
o Very worried (4)
o Extremely worried (5)

concern3 How concerned are you that surveys/polls can manipulate and mislead people?
o Not at all concerned (1)
o A little concerned (2)
o Somewhat concerned (3)
o Very concerned (4)
o Extremely concerned (5)

efficacy1 How confident are you in understanding survey/poll findings in the media?
o Not at all confident (1)
o A little confident (2)
o Somewhat confident (3)
o Very confident (4)
o Extremely confident (5)

efficacy2 How much informed are you in understanding survey/poll findings in the media?
o Not at all informed (1)
o A little informed (2)
o Somewhat informed (3)
o Very informed (4)
o Extremely informed (5)

mc_for_control_w2 How informed do you feel about the game of baseball?
o Not at all informed (1)
o A little informed (2)
o Somewhat informed (3)
o Very informed (4)
o Extremely informed (5)

soup When talking about survey and polls, to explain the idea of representative samples, some scientists make the comparison to tasting a pot of soup. How familiar are you about this soup comparison?
o Not at all familiar (1)
o A little familiar (2)
o Somewhat familiar (3)
o Very familiar (4)
o Extremely familiar (5)

int1 In the past week, did you pay attention to any poll result reported in the media?
o Not at all (1)
o 1 time (2)
o 2 times (3)
o 3 times (4)
o 4 times (5)
o 5 times of more (6)

int2 In the past week, did you read or hear any information about COVID-19 vaccines?
o Not at all (1)
o 1 time (2)
o 2 times (3)
o 3 times (4)
o 4 times (5)
o 5 times of more (6)

int3 In the past week, did you read or hear any information about artificial intelligence (AI)?
o Not at all (1)
o 1 time (2)
o 2 times (3)
o 3 times (4)
o 4 times (5)
o 5 times of more (6)

media1 In the past 14 days, how often have you used the following sources to get news and information?
Print newspapers (1)
TV news (2)
Online news websites (3)
Facebook (4)
Twitter (5)
WhatsApp (6)
Telegram (7)
Instagram (8)
YouTube (9)

- Never (1)
- Once in the past 14 days (2)
- Once per week (3)
- 2-3 times per week (4)
- 4-6 times per week (5)
- Once every day (6)
- 2-3 times every day (7)
- 4-5 times every day (8)
- More than 5 times every day (9)

Additional Outcome Measures (see "Deviations from Pre-registration" section as these outcomes are analyzed for exploratory purposes now given statistical power issue)

**Names**	**Question Wording**	**Details**
Perceived Risk (measured at W1 as pretest and at W2 as outcome variable)	> "How risky do you think [the side effects of COVID-19 vaccines are/ the use of artificial intelligence (AI) technologies in settings such as work, school, and healthcare is]?"<br>> "How concerned are you about [the side effects of COVID-19 vaccines/ the use of artificial intelligence (AI) technologies in settings such as work, school, and healthcare?]"<br>> How worried are you about the [side effects of COVID-19 vaccines/ the use of artificial intelligence (AI) technologies in settings such as work, school, and healthcare]?"	> This is a secondary outcome variable and has two operationalization versions: W2-W1 difference scores and W2 scores alone.<br>> Three items' reliability was high (α=.92). The items were averaged into an index ranging from 0 to 1 where higher scores represent greater risk perceptions<br>> Depending on their manipulation group, half of respondents answered these questions about COVID-19 vaccines and the other half answered about AI.

	> There were five item-specific response options ranging from "Not [risky/concerned/worried] at all" to "Extremely [risky/concerned/worried]."	
Positivity towards Issues (measured at W1 as pretest and at W2 as outcome variable)	> "Overall, how positive or negative do you feel towards [COVID-19 vaccines/ artificial intelligence (AI) technologies]?"<br>> Seven response options ranging from "Strongly negative" to "Strongly positive."	> This is a secondary outcome variable and has two operationalization versions: W2-W1 difference scores and W2 scores alone.<br>> This was a single item measure where higher scores represent greater positivity towards vaccine or AI<br>> Depending on their manipulation group, half of respondents answered these questions about COVID-19 vaccines and the other half answered about AI.
Behavioral Intentions / Policy Views (measured at W1 as pretest and at W2 as outcome variable)	> "On a scale of 1 to 7, where 1 is "strongly unlikely" and 7 is "strongly likely", how likely is it for you to get a COVID-19 vaccine (or booster shots) in future if health authorities recommend it for your age group? Please indicate your preference as a likelihood" with seven response options ranging from "1) Strongly unlikely" to "7) Strongly likely."<br><br>> "Would you support or oppose a policy proposal that aims to regulate more strictly the use of artificial intelligence (AI) technologies in settings such as work, school, and healthcare?"<br>> Seven response options ranging from "Strongly oppose regulation" to "Strongly support regulation."	> This is a secondary outcome variable and there are two operationalization versions: W2-W1 difference scores and W2 scores alone.<br>> Separate single item questions for each issue, where greater scores represent stronger intentions to get vaccinated in future or stronger support for AI regulation policy<br>> Depending on their manipulation group, half of respondents answered these questions about COVID-19 vaccines and the other half answered about AI.<br>> For this question, vaccine and AI focus was quite distinct (behavioral intention vs. policy-support) as we prioritized stronger ecological validity (issue-relevant outcomes). For instance, for AI issue, behavioral intention similar to 'vaccination' did not make sense (e.g. intention to use AI at work) as it involves many factors beyond public opinion (e.g. accessibility, company/school policy), hence we asked about policy-views about regulation of AI.

DEBRIEFINGS

Partial Debriefing at the end of Wave 1 (anonymized by removing authors' info)

Thank you for participating in Part 1 (Survey 1) of this study. A full debrief will be provided after completion of Part 2 (Survey 2) of this study which will happen about 7 to 10 days later.

Some research designs require that the full extent of the study not be explained prior to participation in Survey 2. Although we have described

the general nature of the tasks that you will be asked to perform, the full intent of the study will not be explained to you until after the completion of the study, at the end of Survey 2. At that time, we will provide you with a full debriefing which will include an explanation of the hypothesis that was tested and other relevant background information pertaining to the study. You will also be given an opportunity to ask any questions you have about the hypothesis and the procedures used in the study."

You will receive an invitation to a second and shorter Part 2 survey if you complete this part.

If you have any further queries about the study, please contact the Principal Investigator and they will be more than happy to address any other queries that you may have about the study.

If you decide to withdraw your data after going through the full debriefing document, you can do it. You will still be reimbursed if you decide to withdraw after the full debrief (upon completion of Part 2 of this study)

Please contact the Principal Investigator, Ozan Kuru for all research-related matters and in the event of research-related injuries or inquiries. If you do not plan to join the Survey 2 or end up deciding not joining later on, you can contact the researcher in case you want to learn more about the full purpose and scope of this study. Withdrawals are possible both during submission of this page as well as afterwards.

You may keep a record of this debriefing for your own records by taking a screenshot, a picture of the screen by another device, or copy-pasting it in a document.

Please indicate below if you want to submit your answers and finalize the survey.

If, after reading this debriefing, you want to withdraw your responses from this study, you have the option to do so (click below) before closing this screen.

- ○ **CLICK HERE TO SUBMIT YOUR RESPONSES AND FINALIZE THE SURVEY** (1)
- ○ WITHDRAW MY RESPONSES (2)

Full Debriefing at the end of Wave 2 (anonymized by removing author info)

Please read the debriefing of the study and finalize the survey by clicking the submission and blue arrow at the bottom right of this page:

Debriefing. IMPORTANT NOTIFICATION!

Thank you for your participation in our study, which has the full title as follows: Understanding Methods of Polls. This debriefing session is to clarify and inform you of the true nature of the study, which could not have been revealed at the start of the study because the study design requires that you remain as not fully informed about the content at the beginning in order to measure your reactions. If we had disclosed the full purpose of the study at the beginning, which involved training about polling methodology or different techniques of messaging (as explained below), it could have shaped your judgements directly in evaluating the credibility of hypothetical poll results in this current session, which would introduce validity problems to this experimental scientific research. The experimental purposes needs to be hidden until its effects are measured in this type of research. Hence, we provide more details about the full nature of the study below.

The purpose of this study is to understand whether exposure training about survey/poll methods could equip individuals to be more critical when they encounter survey results. A few different approaches are tested experimentally. These include 1) providing information about methodological details of surveys that are relevant for quality assessments, 2) including interactive quiz with response explanation, 3) warning about misuse and mis-contextualization of poor quality surveys in digital media. Participants were shown surveys with either low or high methodological quality to understand and test the effectiveness of these strategies. We were also interested whether and how your beliefs about science and numeracy skill perceptions can shape your evaluation of information about surveys.
Please note that we have shown you accurate and scientific methodological information about surveys in the training. The surveys you were shown, on the other hand, were hypothetical and not real. These surveys do not include misinformation but were hypothetically created to test your reactions in the given scenario. Please do not take these survey results as evidence of public opinion whether or not their results might be similar to contemporary poll results on these issues in the society. Example surveys/polls are regularly reported in media, as seen in the following links, which are real surveys (not hypothetical):

Example of a recent survey about misinformation beliefs in the society: [**https://www.straitstimes.com/singapore/around-one-in-four-singapore-residents-surveyed-believe-false-claim-about-covid-19-vaccine**]
Example of a recent survey about issue position support levels in the society: [**https://www.channelnewsasia.com/singapore/covid-19-vaccination-making-it-compulsory-singapore-ips-study-2352781**]
Please ignore the hypothetical surveys we showed you. This is an academic study and we do not show these posts to influence your beliefs and decisions. To be critical consumers of surveys in the media, please pay attention to the methodological details such as sampling type (is it

representative?), sample size, margin of error, question wordings and timing of the survey. Please cross-check online information that you suspect with public authorities' recommendations and official sources whenever possible.

If you have any further queries about the study, please contact the Principal Investigator and they will be more than happy to address any other queries that you may have about the study.

If you decide to withdraw your data after going through the debriefing document, you can do it. You will still be reimbursed if you decide to withdraw after the debrief.
Please contact the Principal Investigator Ozan Kuru for all research-related matters and in the event of research-related injuries, or withdrawal requests. Withdrawals are possible both during submission of this page as well as afterwards.

IMPORTANT NOTIFICATION:
The messages (surveys) you were shown in this study were only hypothetical. They are produced for research purposes by incorporating and editing some real examples of recent surveys. Do not take into account this information when making your judgments and decisions.

Participants should not take screenshots of the messages nor share them outside of the research.

These messages are for research purposes only and are not representative nor are they opinions of the research team or the National University of Singapore.

You may keep a record of this debriefing for your own records by taking a screenshot, a picture of the screen by another device, or copy-pasting it in a document.

Please indicate below if you want to submit your answers and finalize the survey. If, after reading this debriefing, you want to withdraw your responses from this study, you have the option to do so (click below) before closing this screen.

○ **CLICK HERE TO SUBMIT YOUR RESPONSES AND FINALIZE THE SURVEY** (1)

○ WITHDRAW MY RESPONSES (2)

H. Descriptive Statistics [click here to return to TOC]

Table. W1 Descriptives

Variable		N	Mean	Std. Dev.	Min	Pctl. 25	Pctl. 50 (median)	Pctl. 75	Max
Age		2027	0.35	(.21)	0.00	0.18	0.33	0.50	1.00
Sex		2027							
	Male	964	0.48						
	Female	1058	0.52						
	Other	5	0.00						
Chinese		2027							
	Not Chinese	369	0.18						
	Chinese	1658	0.82						
Malay		2027							
	Not Malay	1839	0.91						
	Malay	188	0.09						
Indian		2027							
	Not Indian	1938	0.96						
	Indian	89	0.04						
Other Race		2027							
	Not Other Race	1935	0.95						
	Other Race	92	0.05						
Liberal		2027	0.50	(.19)	0.00	0.50	0.50	0.50	1.00
Income		2027	0.38	(.24)	0.00	0.18	0.36	0.55	1.00
Education		2027	0.54	(.27)	0.00	0.33	0.67	0.67	1.00
Interest in Polls (pretest)		2027	0.55	(.20)	0.00	0.42	0.50	0.67	1.00
General Credibility of Poll (pretest)		2027	0.47	(.19)	0.00	0.25	0.50	0.50	1.00
General Concern about Polls (pretest)		2027	0.51	(.22)	0.00	0.25	0.50	0.75	1.00
General Efficacy about Polls (pretest)		2027	0.49	(.20)	0.00	0.25	0.50	0.50	1.00

Vaccine Risk Perceptions (pretest)	2027	0.44	(.25)	0.00	0.25	0.42	0.58	1.00
Vaccine Public Opinion Perceptions (pretest)	2026	0.54	(.26)	0.00	0.25	0.58	0.75	1.00
Vaccine Public Opinion % Perceptions (pretest)	2027	0.39	(.27)	0.00	0.20	0.30	0.60	1.00
Vaccination Intention (pretest)	2027	0.54	(.30)	0.00	0.33	0.50	0.67	1.00
Positivity towards Vaccines (pretest)	2027	0.59	(.25)	0.00	0.50	0.67	0.83	1.00
AI Risk Perceptions (pretest)	2027	0.42	(.22)	0.00	0.25	0.42	0.50	1.00
AI Public Opinion Perceptions (pretest)	2021	0.53	(.26)	0.00	0.25	0.50	0.75	1.00
AI Public Opinion % Perceptions (pretest)	2026	0.38	(.25)	0.00	0.20	0.30	0.50	1.00
AI Policy Support (pretest)	2027	0.67	(.23)	0.00	0.50	0.67	0.83	1.00
Positivity towards AI (pretest)	2027	0.61	(.20)	0.00	0.50	0.67	0.67	1.00
News Literacy Behaviors (pretest)	2027	0.51	(.20)	0.00	0.39	0.50	0.64	1.00
Subjective Numeracy (pretest)	2027	0.56	(.19)	0.03	0.44	0.56	0.69	1.00
Science Literacy (pretest)	2027	0.45	(.25)	0.00	0.25	0.50	0.62	1.00
Severity of Vaccine Side Effect Experience (pretest)	2025	0.34	(.23)	0.00	0.25	0.25	0.50	1.00
Experience Level with AI (pretest)	2027	0.40	(.35)	0.00	0.00	0.50	0.75	1.00

Table. W2 Descriptives (Longitudinal Main Analysis Sample)

Variable	N	Mean	Std. Dev.	Min	Pctl. 25	Pctl. 50 (median)	Pctl. 75	Max
Age	1076	0.37	(.21)	0.00	0.21	0.34	0.52	1.00
Sex	1076							
Male	558	0.52						
Female	514	0.48						
Other	4	0.00						
Chinese	1076							
Not Chinese	168	0.16						
Chinese	908	0.84						

Malay	1076							
Not Malay	989	0.92						
Malay	87	0.08						
Indian	1076							
Not Indian	1046	0.97						
Indian	30	0.03						
Other Race	1076							
Not Other Race	1025	0.95						
Other Race	51	0.05						
Liberal	1076	0.50	(.19)	0.00	0.50	0.50	0.50	1.00
Income	1076	0.38	(.24)	0.00	0.18	0.36	0.55	1.00
Education	1076	0.55	(.28)	0.00	0.33	0.67	0.67	1.00
Interest in Polls (pretest)	1076	0.57	(.19)	0.00	0.42	0.58	0.75	1.00
General Credibility of Poll (pretest)	1076	0.48	(.19)	0.00	0.25	0.50	0.50	1.00
General Concern about Polls (pretest)	1076	0.50	(.22)	0.00	0.25	0.50	0.75	1.00
General Efficacy about Polls (pretest)	1076	0.50	(.19)	0.00	0.50	0.50	0.50	1.00
Vaccine Risk Perceptions (pretest)	1076	0.44	(.24)	0.00	0.25	0.42	0.58	1.00
Vaccine Public Opinion Perceptions (pretest)	1075	0.54	(.26)	0.00	0.25	0.58	0.75	1.00
Vaccine Public Opinion % Perceptions (pretest)	1076	0.39	(.27)	0.00	0.20	0.30	0.60	1.00
Vaccination Intention (pretest)	1076	0.54	(.29)	0.00	0.33	0.50	0.67	1.00
Positivity towards Vaccines (pretest)	1076	0.59	(.24)	0.00	0.50	0.67	0.83	1.00
AI Risk Perceptions (pretest)	1076	0.42	(.22)	0.00	0.25	0.42	0.50	1.00
AI Public Opinion Perceptions (pretest)	1073	0.52	(.26)	0.00	0.25	0.50	0.75	1.00
AI Public Opinion % Perceptions (pretest)	1075	0.38	(.24)	0.00	0.20	0.30	0.50	1.00
AI Policy Support (pretest)	1076	0.68	(.22)	0.00	0.50	0.67	0.83	1.00
Positivity towards AI (pretest)	1076	0.61	(.20)	0.00	0.50	0.67	0.67	1.00
News Literacy Behaviors (pretest)	1076	0.50	(.20)	0.00	0.39	0.50	0.62	1.00
Subjective Numeracy (pretest)	1076	0.56	(.19)	0.06	0.44	0.56	0.69	1.00

Science Literacy (pretest)	1076	0.45	(.25)	0.00	0.25	0.50	0.62	1.00
Severity of Vaccine Side Effect Experience (pretest)	1074	0.34	(.23)	0.00	0.25	0.25	0.50	1.00
Experience Level with AI (pretest)	1076	0.39	(.35)	0.00	0.00	0.50	0.75	1.00

Table. Descriptives for Dropouts (did not complete W2 survey)

**Variable**	**N**	**Mean**	**Std. Dev.**	**Min**	**Pctl. 25 (median)**	**Pctl. 50**	**Pctl. 75**	**Max**
Age	951	0.33	(.21)	0.00	0.16	0.29	0.47	0.95
Sex	951							
Male	406	0.43						
Female	544	0.57						
Other	1	0.00						
Chinese	951							
Not Chinese	201	0.21						
Chinese	750	0.79						
Malay	951							
Not Malay	850	0.89						
Malay	101	0.11						
Indian	951							
Not Indian	892	0.94						
Indian	59	0.06						
Other Race	951							
Not Other Race	910	0.96						
Other Race	41	0.04						
Liberal	951	0.51	(.20)	0.00	0.50	0.50	0.50	1.00
Income	951	0.38	(.23)	0.00	0.18	0.36	0.50	1.00
Education	951	0.53	(.27)	0.00	0.33	0.67	0.67	1.00

Interest in Polls (pretest)	951	0.54	(.20)	0.00	0.42	0.50	0.67	1.00
General Credibility of Poll (pretest)	951	0.47	(.18)	0.00	0.25	0.50	0.50	1.00
General Concern about Polls (pretest)	951	0.52	(.22)	0.00	0.38	0.50	0.75	1.00
General Efficacy about Polls (pretest)	951	0.48	(.20)	0.00	0.25	0.50	0.50	1.00
Vaccine Risk Perceptions (pretest)	951	0.43	(.25)	0.00	0.25	0.42	0.58	1.00
Vaccine Public Opinion Perceptions (pretest)	951	0.55	(.26)	0.00	0.25	0.50	0.75	1.00
Vaccine Public Opinion % Perceptions (pretest)	951	0.39	(.27)	0.00	0.20	0.30	0.60	1.00
Vaccination Intention (pretest)	951	0.53	(.31)	0.00	0.33	0.50	0.67	1.00
Positivity towards Vaccines (pretest)	951	0.59	(.25)	0.00	0.50	0.67	0.83	1.00
AI Risk Perceptions (pretest)	951	0.42	(.22)	0.00	0.25	0.42	0.50	1.00
AI Public Opinion Perceptions (pretest)	948	0.54	(.26)	0.00	0.25	0.50	0.75	1.00
AI Public Opinion % Perceptions (pretest)	951	0.38	(.25)	0.00	0.20	0.30	0.60	1.00
AI Policy Support (pretest)	951	0.66	(.23)	0.00	0.50	0.67	0.83	1.00
Positivity towards AI (pretest)	951	0.61	(.21)	0.00	0.50	0.67	0.83	1.00
News Literacy Behaviors (pretest)	951	0.51	(.20)	0.00	0.39	0.50	0.67	1.00
Subjective Numeracy (pretest)	951	0.56	(.19)	0.03	0.44	0.56	0.69	1.00
Science Literacy (pretest)	951	0.45	(.25)	0.00	0.25	0.50	0.62	1.00
Severity of Vaccine Side Effect Experience (pretest)	951	0.35	(.23)	0.00	0.25	0.25	0.50	1.00
Experience Level with AI (pretest)	951	0.42	(.35)	0.00	0.00	0.50	0.75	1.00

Supplementary Material 2: Preregistration and Deviations from Preregistration Explained [click here to return to TOC]

This section presents 1) the full preregistration details (which are publicly available with time stamp at *#154000, https://aspredicted.org/FD9_JCT*) and 2) deviations and discussions of deviations from the preregistration.

1) Preregistration

1) Have any data been collected for this study already?
No, no data have been collected for this study yet.

2) What's the main question being asked or hypothesis being tested in this study?
H1: Respondents in the control (W1-1) condition A) will not differ in their poll credibility perceptions when exposed to high vs. low quality polls and B) will move in the direction of poll results in their risk and public opinion perceptions regardless of poll quality.

H2: A) Respondents in intervention-groups will confer greater creditability to high quality polls (and lower credibility to low quality polls) as opposed to control (W1-1 < W1-2, W1-3, W1-4) and B) will move in the direction of poll results in their risk and public opinion perceptions more for high quality than low quality polls (W1-1 < W1-2, W1-3, W1-4 for high quality polls).

H3: The effects of inoculation will be greater than active-intervention which will be greater than literacy training only interventions. Hence the order of intervention effectiveness in H2 will vary as follows; literacy training will have smaller effect than literacy training with quiz session, and literacy training with quiz session will have smaller effect than the inoculation intervention: W1-2 < W1-3 < W1-4.

H4: Those with stronger A) subjective numeracy, B) everyday scientific literacy, and C) news literacy behaviors will be more responsive to interventions (H2, and H3).

RQ1: Will respondents discredit high and low quality polls equally (on non-polarizing issues of health and AI) of which results are unfavorable to their pre-existing issue positions more? (moderation of H1 by issue position)
(pretest risk perceptions used as moderator and this RQ only for poll credibility and public opinion perceptions outcomes)

RQ2: Will intervention effectiveness (H2 and H3) be moderated by pre-existing issue positions? (moderation of H2 and H3 by issue position)
(pretest risk perceptions used as moderator and this RQ only for poll credibility and public opinion perception outcome variables)

Exploratory:

- General beliefs (credibility judgments) of polls in media (W1 pre-post measures) as an additional outcome variable for W1 interventions only
- Education levels as moderator
- Trust in science as moderator

3) Describe the key dependent variable(s) specifying how they will be measured.
To be tested both 1) (primarily) pre- and post-test differences across waves and 2) post-test cross-sectionally:
- Perceived credibility of polls: 5 items: reliable, accurate, valid, credible, scientific
- Perceived risk of issue: 3 items: risky, concerned, worried (respondent)
- Perceptions of public opinion: 3 items: risky, concerned, worried (most people in country) + 1 item % prediction for issue position in country
- Intentions: 1 item, 7 point response
- Overall feelings towards issue: 1 item positivity-negativity

4) How many and which conditions will participants be assigned to?
This is a 2-wave panel experiment where conditions emerge from the crossing of manipulations at both waves:

Cross-sectionally, there are 4 conditions
Longitudinally, there are 4 X 8 = 32 conditions
Participants will be randomly assigned to conditions.
Wave 1: none vs. literacy passive vs. literacy active vs. inoculation
Condition W1-1: Control
Condition W1-2: training
Condition W1-3: training + quiz
Condition W1-4: training + quiz + warning
Wave 2: result X methodological quality X issue replication
Condition W2-1: issue 1, result majority pro, low quality
Condition W2-2: issue 1, result majority pro, high quality
Condition W2-3: issue 1, result majority anti, low quality
Condition W2-4: issue 1, result majority anti, high quality
Condition W2-5: issue 2, result majority pro, low quality
Condition W2-6: issue 2, result majority pro, high quality
Condition W2-7: issue 2, result majority anti, low quality

Condition W2-8: issue 2, result majority anti, high quality

5) Specify exactly which analyses you will conduct to examine the main question/hypothesis.
Both cross-sectional and longitudinal analyses with ANCOVA and/or multiple regression models will be conducted. Longitudinally, within-person difference/change scores will also be calculated and used as outcome variable too. As outcome variables are continuous, OLS based variance models will be used. Moderators will be tested with interaction tests.
Experimental tests will be conducted both across all 32 conditions separately as well as in separate combination for 16 X 2 conceptual replications (e.g. by combining vaccine and AI conceptual replication conditions)

6) Describe exactly how outliers will be defined and handled, and your precise rule(s) for excluding observations.
Adult individuals in Singapore (21+ years old) are recruited into the study. Demographic quotas are used to represent the country as much as possible. There is consent form, accuracy pledge, and a couple of attention check questions. Manipulation check failures will not be removed. Participants who state 'withdraw' after debriefing (which happens very rarely) will also be excluded from analysis to honor their request.

7) How many observations will be collected or what will determine sample size?
No need to justify decision, but be precise about <u>exactly</u> how the number will be determined.
This is a 2 wave panel online survey experiment. Wave 1 N = 2000 and targeted Wave 2 N = 1100 although W2 recontact rates are estimates only and W1 N is determined by power analysis and expected attrition rates in consultation with the survey company as well as budget consideration.

8) Anything else you would like to pre-register?
(e.g., secondary analyses, variables collected for exploratory purposes, unusual analyses planned?)
Interference measure: Respondents are asked whether and how many times they read information about polls, COVID-19 vaccines, and AI in the last ten days.
There are manipulation check questions too."
[End of Preregistration]
2) Deviations from Preregistration

- Some hypotheses and RQs have been reorganized and re-numbered for improved flow of argument in the manuscript reporting (H4 and RQ1-2).
- RQ1 and 2 have been renumbered to RQ2 and RQ3 respectively, and a new RQ1 is added. The new RQ1 is already implicitly suggested in the pre-registration by relying on prior research findings, but to be clear to readers this is now explicitly stated.

- H1 through H4 had expectations about the second set of outcome variables in preregistration while the manuscript primarily focuses on perceived credibility; most of these are reported only in the Supplementary Files. We instead re-organize these for clarity and report outcomes other than perceived credibility in the new H5 in the manuscript.
- Preregistration listed education moderation as exploratory, but upon further theoretical framing updates during the writing of the paper (e.g. recent studies examining poll perceptions used education as moderator), we decided to organize most moderators as indexing individuals' existing abilities to understand polls (e.g. education, science literacy, subjective numeracy, digital news literacy behaviors), hence we decided to present education results as part of main findings.
- Preregistration mentioned that we would utilize ANCOVA and OLS regressions, but to be consistent and for ease, we relied on OLS only. ANCOVA and OLS share the same background computation but OLS results were more straightforward. This was especially the case with our individual difference moderator analyses.
- It is evident that at W2, the primary/key manipulation is methodological quality levels of polls presented (as interventions at W1 target them), while poll result (ecological validity and symmetry in design) and issue type (conceptual replication) are secondary. We did not clarify this in the preregistration, we just listed the three manipulations at W2 as if they were of equal importance. Note that when we elaborated and clarified methodological quality is the key manipulation at W2 (not mentioned in preregistration), this is purely important for framing of the paper and did not change anything in methodological and analytical procedures of the study. Analytically, all three interventions are treated on an equal level, because they were designed on an equal (symmetric and equivalent) manner.
- REVISION UPDATE: Upon reviewer feedback about reduced power of individual difference moderator tests, we came up with new modelling strategy for these tests (H4 and RQ1-2-3) which are clearly marked as such in the manuscript (that they are not pre-registered): We pooled interventions and tested them collectively in interaction with individual difference pretest scores.
- REVISION UPDATE: We also compared public opinion perceptions across W1 and W2; however, these models were underpowered as they were based on split samples (issue-specific nature of outcome variables) and involved three-way interactions. Due to the unidirectional nature of the outcome variable, the effects must be conditioned on poll result directionality, introducing another interaction term for the proper testing of the hypothesis).

3) General Discussion

There are no deviations in the design of the study (conditions, all outcome and predictor measures, etc.). There are some deviations, made in response to reviewer feedback during revisions, which are marked so. We also disclose that we did not conduct any pilot tests for this study before its pre-registration. While there is nothing wrong with pilot testing and it is valued by many scholars, this disclosure is important, because some scholars pointed out that unreported or not-preregistered pilot testing before conducting pre-registered main studies has been a mechanism of bypassing the temporal premise of pre-registration (McDermott, 2022). The ideal approach for scholars who utilize pilot tests might be to disclose them and preregister the pilot tests as well. In conclusion, we believe our comparisons do not pose a significant post-hoc rationalization risk.

Supplementary Material 3: Equivalence of Conditions and Cell Balance Tests [click here to return to TOC]

Cell Balances

Balance tests: The cell sample sizes were balanced; although there was some slight variation due to natural fallout from W1 to W2. However, the Chi-squared test showed that this cell sample size variation (which ranged from 2.04% to 4.46% in each cell among all 32 conditions) was not significant ($x^2(21)$= 31.316, ns.). See the table for detailed Ns for each manipulation and cell.

Table. Cell sample size balances across conditions for the Effective Sample (Main Analysis of N=1076).

	**Conditions**	**Cross-conditions**	**N**	**% of Total N=**
**Manipulation W1**	Control	273		25.37%
	Intervention 1 (Passive literacy)	269		25.00%
	Intervention 2 (Active literacy)	263		24.44%
	Intervention 3 (Inoculation)	**271**		25.19%
	**Total**	**1076**		**100.00%**
**Manipulation W2**	Message 1 (Low quality, Majority Concerned, Vaccine)	136		12.64%
	Message 2 (High quality, Majority Concerned, Vaccine)	133		12.36%
	Message 3 (Low quality, Majority Confident, Vaccine)	136		12.64%
	Message 4 (High quality, Majority Confident, Vaccine)	133		12.36%
	Message 5 (Low quality, Majority Support, AI)	133		12.36%
	Message 6 (High quality, Majority Support, AI)	133		12.36%
	Message 7 (Low quality, Majority Oppose, AI)	137		12.73%
	Message 8 (High quality, Majority Oppose, AI)	135		12.55%
	**Total**	**1076**		**100.00%**
**W1 X W2 Conditions**	Control	Message 1	41	3.81%
		Message 2	32	2.97%

	Message 3	29	2.70%
	Message 4	38	3.53%
	Message 5	30	2.79%
	Message 6	26	2.42%
	Message 7	29	2.70%
	Message 8	48	4.46%
Intervention 1	Message 1	37	3.44%
	Message 2	41	3.81%
	Message 3	39	3.62%
	Message 4	27	2.51%
	Message 5	32	2.97%
	Message 6	30	2.79%
	Message 7	41	3.81%
	Message 8	**22**	2.04%
Intervention 2	Message 1	32	2.97%
	Message 2	28	2.60%
	Message 3	38	3.53%
	Message 4	34	3.16%
	Message 5	29	2.70%
	Message 6	35	3.25%
	Message 7	31	2.88%
	Message 8	36	3.35%
Intervention 3	Message 1	26	2.42%
	Message 2	32	2.97%
	Message 3	30	2.79%
	Message 4	34	3.16%
	Message 5	42	3.90%
	Message 6	42	3.90%

		Message 7	36	3.35%
		Message 8	29	2.70%
**Total**			**1076**	**100.00%**
**Dropouts from W1 to W2 by W1 conditions***	Control	227		23.86%
	Intervention 1 (Passive literacy)	242		25.47%
	Intervention 2 (Active literacy)	244		25.66%
	Intervention 3 (Inoculation)	238		25.03%
**Total**		**951**		**100.00%**

Notes. There was no differential attrition among W1 groups.

Equivalence of Conditions

Balance tests F-tests were conducted to see if any of the W1 conditions differed from each other according to the pre-test measures and demographic characteristics. Random assignment worked for almost all variables, and the equivalence of conditions has been achieved. For the few variables that had significant imbalances, we included them as covariates. See details as well as models that included either no covariates at all or additional further covariates as mentioned in the main paper.

Table. Equivalence by WAVE 1

**manips_w1**	**1**			**2**			3			4			
**Variable**	**N**	**Mean**	**SD**	**N**	**Mean**	**SD**	**N**	**Mean**	**SD**	**N**	**Mean**	**SD**	**Test**
Age	500	0.35	0.21	511	0.35	0.22	507	0.34	0.22	509	0.35	0.21	F=0.187
Sex	500			511			507			509			
Chinese	500	0.81	0.39	511	0.84	0.37	507	0.81	0.39	509	0.81	0.39	F=0.595
Malay	500	0.088	0.28	511	0.084	0.28	507	0.097	0.3	509	0.1	0.3	F=0.403
Indian	500	0.044	0.21	511	0.041	0.2	507	0.043	0.2	509	0.047	0.21	F=0.076
Other Race	500	0.058	0.23	511	0.037	0.19	507	0.049	0.22	509	0.037	0.19	F=1.191

Liberal	500	0.5	0.2	511	0.51	0.19	507	0.5	0.18	509	0.51	0.2	F=0.593
Income	500	0.39	0.23	511	0.37	0.23	507	0.37	0.24	509	0.37	0.24	F=1.204
Education	500	0.54	0.28	511	0.55	0.27	507	0.53	0.27	509	0.54	0.27	F=0.752
Interest in Polls	500	0.56	0.18	511	0.56	0.21	507	0.54	0.19	509	0.56	0.2	F=1.548
Polls credible in general	500	0.47	0.18	511	0.48	0.19	507	0.46	0.19	509	0.47	0.19	F=0.822
Poll concern in general	500	0.5	0.23	511	0.52	0.23	507	0.51	0.22	509	0.51	0.22	F=0.863
Poll efficacy in general	500	0.49	0.2	511	0.49	0.2	507	0.48	0.19	509	0.49	0.2	F=0.413
Pretest vaccine risk perceptions	500	0.42	0.25	511	0.44	0.24	507	0.44	0.24	509	0.45	0.25	F=1.995
Pretest vaccine public opinion perceptions	499	0.55	0.26	511	0.54	0.26	507	0.54	0.27	509	0.55	0.26	F=0.06
Pretest vaccine % public opinion perceptions	500	0.39	0.27	511	0.38	0.27	507	0.38	0.26	509	0.39	0.27	F=0.699
Pretest vaccine intention	500	0.54	0.29	511	0.52	0.29	507	0.56	0.31	509	0.51	0.3	F=2.492
Pretest overall vaccine positivity	500	0.6	0.24	511	0.6	0.24	507	0.59	0.24	509	0.58	0.26	F=1.005
Pretest AI risk perceptions	500	0.42	0.21	511	0.42	0.22	507	0.42	0.22	509	0.42	0.22	F=0.016
Pretest AI public opinion perceptions	499	0.51	0.26	508	0.55	0.26	507	0.52	0.26	507	0.54	0.26	F=1.893
Pretest AI % public opinion perceptions	500	0.4	0.25	510	0.38	0.25	507	0.36	0.24	509	0.39	0.25	F=2.431
Pretest AI policy support	500	0.66	0.23	511	0.67	0.22	507	0.67	0.22	509	0.69	0.24	F=1.328
Pretest overall AI positivity	500	0.61	0.2	511	0.62	0.2	507	0.59	0.2	509	0.61	0.21	F=1.115
News Literacy Behaviors	500	0.52	0.2	511	0.51	0.19	507	0.5	0.2	509	0.51	0.21	F=0.736
Subjective Numeracy	500	0.56	0.19	511	0.55	0.19	507	0.55	0.19	509	0.57	0.19	F=0.761
Science Literacy	500	0.48	0.26	511	0.44	0.25	507	0.44	0.25	509	0.44	0.24	F=2.866*

Statistical significance markers: * p<0.05; ** p<0.01; *** p<0.001.

Table. Equivalence by WAVE 2 – Part 1/2 of the Table

	**0**			**1**			**2**			**3**		
**Variable**	**N**	**Mean**	**SD**	**N**	**Mean**	**SD**	**N**	**Mean**	**SD**	**N**	**Mean**	**SD**
Age	951	0.33	0.21	136	0.36	0.21	133	0.38	0.21	136	0.37	0.21
Sex	951			136			133			136		
Chinese	951	0.79	0.41	136	0.81	0.39	133	0.79	0.41	136	0.85	0.36

Malay	951	0.11	0.31	136	0.11	0.31	133	0.11	0.31	136	0.074	0.26
Indian	951	0.062	0.24	136	0.022	0.15	133	0.03	0.17	136	0.015	0.12
Other Race	951	0.043	0.2	136	0.059	0.24	133	0.075	0.26	136	0.059	0.24
Liberal	951	0.51	0.2	136	0.5	0.18	133	0.52	0.18	136	0.47	0.17
Income	951	0.38	0.23	136	0.38	0.24	133	0.39	0.25	136	0.38	0.26
Education	951	0.53	0.27	136	0.56	0.28	133	0.56	0.3	136	0.55	0.27
Interest in Polls	951	0.54	0.2	136	0.58	0.19	133	0.59	0.19	136	0.55	0.21
Polls credible in general	951	0.47	0.18	136	0.51	0.2	133	0.47	0.19	136	0.48	0.2
Poll concern in general	951	0.52	0.22	136	0.51	0.24	133	0.52	0.22	136	0.52	0.22
Poll efficacy in general	951	0.48	0.2	136	0.52	0.21	133	0.5	0.2	136	0.52	0.2
Pretest vaccine risk perceptions	951	0.43	0.25	136	0.46	0.27	133	0.45	0.25	136	0.43	0.23
Pretest vaccine public opinion perceptions	951	0.55	0.26	136	0.55	0.26	133	0.53	0.25	135	0.54	0.25
Pretest vaccine % public opinion perceptions	951	0.39	0.27	136	0.39	0.28	133	0.36	0.26	136	0.38	0.27
Pretest vaccine intention	951	0.53	0.31	136	0.52	0.32	133	0.52	0.28	136	0.54	0.29
Pretest overall vaccine positivity	951	0.59	0.25	136	0.56	0.26	133	0.61	0.25	136	0.62	0.23
Pretest AI risk perceptions	951	0.42	0.22	136	0.44	0.24	133	0.45	0.21	136	0.38	0.22
Pretest AI public opinion perceptions	948	0.54	0.26	135	0.54	0.26	133	0.55	0.25	135	0.54	0.26
Pretest AI % public opinion perceptions	951	0.38	0.25	136	0.39	0.25	133	0.35	0.23	136	0.36	0.24
Pretest AI policy support	951	0.66	0.23	136	0.67	0.23	133	0.67	0.23	136	0.69	0.21
Pretest overall AI positivity	951	0.61	0.21	136	0.6	0.21	133	0.6	0.2	136	0.63	0.19
News Literacy Behaviors	951	0.51	0.2	136	0.52	0.2	133	0.48	0.19	136	0.49	0.22
Subjective Numeracy	951	0.56	0.19	136	0.59	0.19	133	0.56	0.19	136	0.55	0.2
Science Literacy	951	0.45	0.25	136	0.43	0.28	133	0.48	0.25	136	0.45	0.25

Table. Equivalence by WAVE 2 – Part 2/2 of the Table

	4			5			6			7			8			
**Variable**	**N**	**Mean**	**SD**	**N**	**Mean**	**SD**	**N**	**Mean**	**SD**	**N**	**Mean**	**SD**	**N**	**Mean**	**SD**	**Test**
Age	133	0.36	0.22	133	0.36	0.2	133	0.4	0.22	137	0.34	0.23	135	0.38	0.21	F=3.074**

Sex	133			133			133			137			135			
Chinese	133	0.83	0.37	133	0.87	0.34	133	0.85	0.36	137	0.88	0.33	135	0.87	0.34	F=2.06**
Malay	133	0.083	0.28	133	0.06	0.24	133	0.068	0.25	137	0.073	0.26	135	0.074	0.26	F=0.926
Indian	133	0.015	0.12	133	0.03	0.17	133	0.053	0.22	137	0.015	0.12	135	0.044	0.21	F=2.343*
Other Race	133	0.068	0.25	133	0.038	0.19	133	0.03	0.17	137	0.036	0.19	135	0.015	0.12	F=1.196
Liberal	133	0.52	0.19	133	0.48	0.19	133	0.5	0.18	137	0.51	0.2	135	0.48	0.18	F=1.363
Income	133	0.37	0.23	133	0.37	0.24	133	0.38	0.24	137	0.37	0.24	135	0.36	0.23	F=0.167
Education	133	0.53	0.26	133	0.54	0.27	133	0.56	0.27	137	0.53	0.29	135	0.54	0.26	F=0.491
Interest in Polls	133	0.54	0.2	133	0.59	0.17	133	0.54	0.19	137	0.57	0.2	135	0.57	0.2	F=2.628**
Polls credible in general	133	0.48	0.18	133	0.47	0.19	133	0.46	0.18	137	0.48	0.18	135	0.47	0.19	F=1.111
Poll concern in general	133	0.49	0.22	133	0.51	0.25	133	0.5	0.23	137	0.51	0.21	135	0.47	0.22	F=1.135
Poll efficacy in general	133	0.49	0.17	133	0.5	0.21	133	0.48	0.19	137	0.49	0.18	135	0.5	0.19	F=1.653
Pretest vaccine risk perceptions	133	0.41	0.24	133	0.49	0.23	133	0.44	0.24	137	0.43	0.23	135	0.43	0.25	F=1.117
Pretest vaccine public opinion perceptions	133	0.57	0.26	133	0.54	0.27	133	0.55	0.27	137	0.54	0.27	135	0.53	0.27	F=0.37
Pretest vaccine % public opinion perceptions	133	0.38	0.28	133	0.4	0.27	133	0.38	0.27	137	0.39	0.27	135	0.39	0.27	F=0.26
Pretest vaccine intention	133	0.58	0.28	133	0.53	0.29	133	0.48	0.29	137	0.55	0.3	135	0.59	0.29	F=1.644
Pretest overall vaccine positivity	133	0.62	0.23	133	0.56	0.25	133	0.57	0.26	137	0.58	0.23	135	0.6	0.24	F=1.228
Pretest AI risk perceptions	133	0.43	0.21	133	0.45	0.21	133	0.42	0.21	137	0.4	0.22	135	0.4	0.21	F=1.793
Pretest AI public opinion perceptions	133	0.51	0.27	132	0.53	0.26	133	0.51	0.26	137	0.5	0.25	135	0.5	0.26	F=0.657
Pretest AI % public opinion perceptions	133	0.39	0.24	132	0.39	0.24	133	0.4	0.25	137	0.41	0.25	135	0.37	0.24	F=0.949
Pretest AI policy support	133	0.68	0.23	133	0.68	0.24	133	0.7	0.22	137	0.68	0.23	135	0.69	0.2	F=0.685
Pretest overall AI positivity	133	0.61	0.18	133	0.59	0.21	133	0.61	0.2	137	0.62	0.21	135	0.59	0.19	F=0.483
News Literacy Behaviors	133	0.51	0.19	133	0.53	0.21	133	0.49	0.21	137	0.51	0.19	135	0.49	0.18	F=0.814
Subjective Numeracy	133	0.53	0.19	133	0.56	0.2	133	0.53	0.18	137	0.57	0.18	135	0.57	0.19	F=1.406
Science Literacy	133	0.43	0.26	133	0.48	0.24	133	0.42	0.25	137	0.47	0.25	135	0.46	0.25	F=1.146

Statistical significance markers: * p<0.05; ** p<0.01; *** p<0.001.

Supplementary Material 4: Manipulation Checks [click here to return to TOC]

We employed a comprehensive set of manipulation check questions, categorized under three areas:

a) Analysis of responses to open-ended questions (for W2 manipulations) – Please see word cloud analyses further below.
   i) There are important differences between control and intervention conditions, and increasingly so going from passive to active and then to inoculation intervention. Compare how each W1 group differs in terms of how W2 low-quality vs. high-quality poll readers wrote their answers. Example: "Probability" in inoculation high-quality condition is the biggest (most frequent word) used by respondents compared to low-quality inoculation and compared to control and other interventions.
b) Recognition of correct/incorrect questions (for W2 manipulations)
   i) Strategy 1: general question for issue recognition:
      (1) Only 14 respondents mislabelled/misrecognized whether they had seen a vaccine or AI-related poll. 13 of the 14 respondents selected the correct answer when prompted for a second time in a follow-up question which urged them to be careful.
   ii) Strategy 2: Comparing the correct response with an unrelated item: cryptocurrencies
      (1) All respondents (N=269) who were exposed to polls showing majority concern about vaccines correctly identified this information about public opinion and did not choose the opposite answer (i.e. majority confidence about vaccines). Only 1 respondent out of N=269 (other half) incorrectly identified the poll showing majority confidence about vaccines.
      (2) All respondents who were shown (N=266) poll showing majority support for AI regulation (and concern about AI) identified it correctly. On the other hand, 6 out of N=272 who saw majority opposition to AI regulation results gave an incorrect response.
   iii) Strategy 3: Comparing the correct response with the plausible and related item: concern vs. confidence for vaccine issue and support vs. opposition to AI regulation for AI issue
      (1) When asked about majority concern vs. confidence about vaccine polls directly, 11 out of N=269 for concern and 88 out of 269 for confidence were not correct.
      (2) When asked about majority support vs. opposition about AI polls directly, 64 out of N=272 for the opposition poll and 13 out of 266 for the support poll were not correct.
c) Evaluation questions (for W1 manipulations, as measured at both W1 and W2) – Please see the t-test comparison tables further below. These questions tapped specific content in each intervention and the related constructs to gauge how manipulations worked.
   i) As seen in the tables, intervention conditions changed evaluations for most variables as opposed to control.
   ii) Control condition (W1-baseball game) differed in terms of familiarity with baseball too.
   iii) Most notably, respondents of W1 interventions performed better in objective quiz questions about poll methods by the end of W2 as well.
   iv) Finally, we directly asked about the soup analogy for representative probability-based samples that was mentioned in all three interventions at W1 (in the passive literacy information delivery component). For question-wording, see the survey instrument details

elsewhere in this appendix. We asked this question at the end of W2 too. All three intervention conditions reported significantly higher familiarity at the end of W1 with the soup analogy than the control group at W1.

(1) Pairwise Tukey corrected differences:
   (a) Passive literacy – Control: M_{diff}=.33, p=.001
   (b) Active literacy – Control: M_{diff}=.24, p=.032
   (c) Inoculation – Control: M_{diff}=.25, p=.025

General Conclusion: Collectively, we are confident both W1 and W2 manipulations worked as designed. While this is expected given the main findings in hypothesis testing, these extensive and detailed manipulation checks provide important evidence about how the manipulations worked.

See the Figure and Tables on the next pages.

Figure. Word clouds representing open-ended response text entries by respondents in describing the poll they were each exposed to (W2), across pre-emptive interventions (W1) “What was the type of sample of the survey/poll you just read in the news story? Please write”

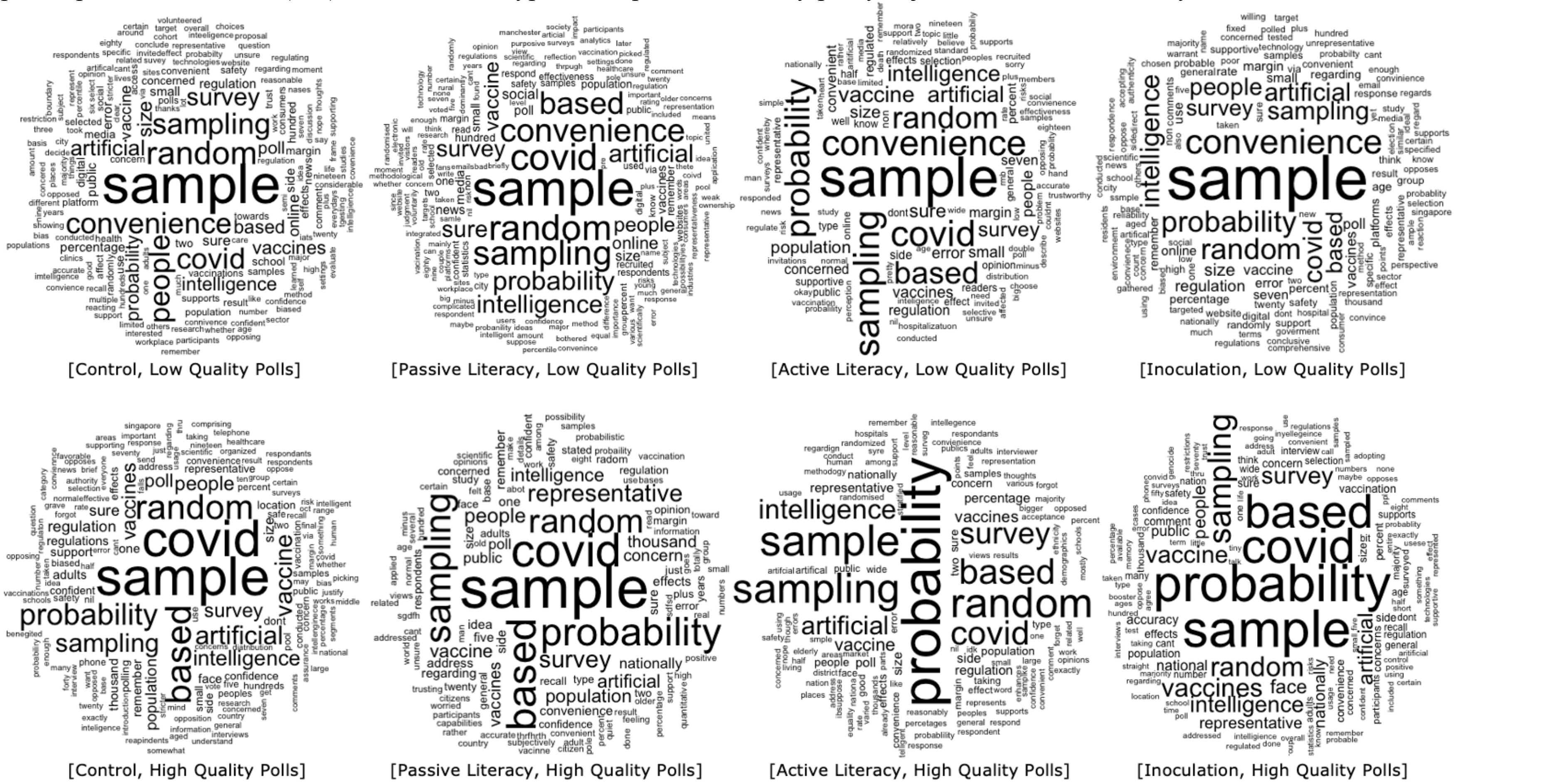

Control vs. Intervention Conditions Combined

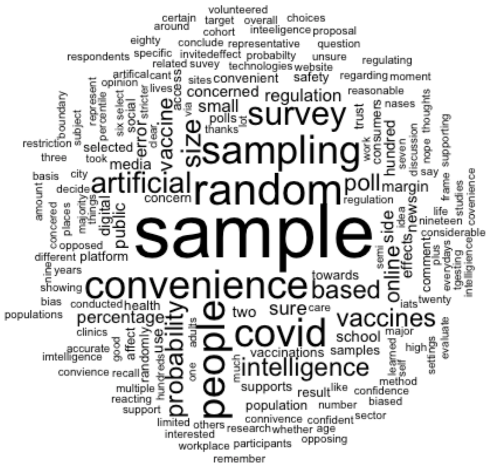

[Control Group, Low Quality Polls]

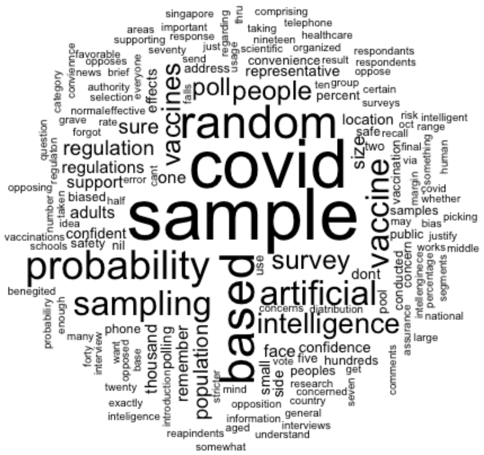

[Control Group, High Quality Polls]

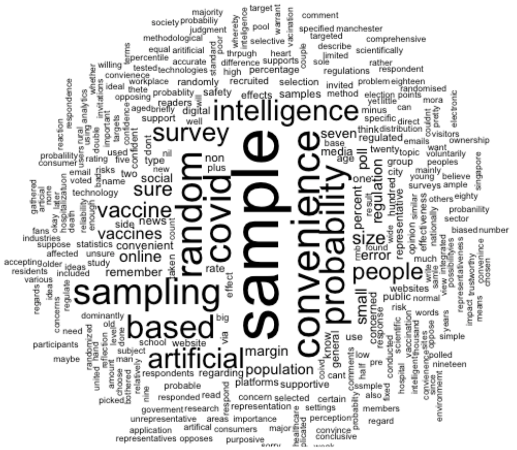

[Intervention Groups Combined, Low Quality Polls]

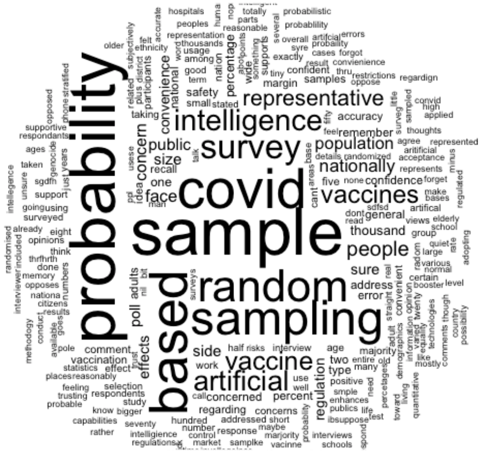

[Intervention Groups Combined, High Quality Polls]

Table. Manipulation Checks for W1 Manipulations at W1

	Control (W1)			Int1: Passive Literacy (W1)			Int2: Active Literacy (W1)			Int3: Inoculation (W1)			
**Variable**	**N**	**Mean**	**SD**	**N**	**Mean**	**SD**	**N**	**Mean**	**SD**	**N**	**Mean**	**SD**	**Test**
Misinformation polls familiarity	499	0.44	(.25)	510	0.47	(.24)	507	0.47	(.24)	509	0.55	(.25)	F=17.869***
Twitter polls familiarity	483	0.31	(.29)	494	0.31	(.30)	490	0.29	(.29)	492	0.32	(.30)	F=0.976
Informed about baseball	484	0.41	(.25)	495	0.21	(.26)	491	0.18	(.23)	492	0.2	(.25)	F=88.907***
General Poll Credibility Difference (W1 post – W1 pre)	500	-0.069	(.19)	511	-0.073	(.20)	507	-0.073	(.18)	509	-0.098	(.19)	F=2.495*
General Poll Credibility Index (W1 posttest)	500	0.41	(.17)	511	0.42	(.18)	507	0.41	(.17)	509	0.39	(.17)	F=3.411**
General Concern about Polls (W1 post)	500	0.52	(.25)	511	0.56	(.23)	507	0.53	(.22)	509	0.6	(.23)	F=12.723***
General Poll Concern Difference (W1 post – W1 pre)	500	0.018	(.22)	511	0.033	(.23)	507	0.023	(.22)	509	0.092	(.22)	F=12.275***
General Poll Concern Index (W1 posttest)	500	0.5	(.23)	511	0.52	(.22)	507	0.5	(.21)	509	0.58	(.22)	F=14.204***
General Poll Efficacy (W1 posttest)	500	0.47	(.22)	511	0.48	(.21)	507	0.48	(.21)	509	0.47	(.22)	F=0.355
General Poll Efficacy Difference (W1 post – W1 pre)	500	-0.018	(.20)	511	-0.0073	(.20)	507	-0.0015	(.20)	509	-0.025	(.21)	F=1.366
General Poll Efficacy Index (W1 posttest)	500	0.47	(.20)	511	0.48	(.20)	507	0.47	(.20)	509	0.46	(.21)	F=0.542

Statistical significance markers: *** p<0.1; ** p<0.05; *** p<0.01**

Table. Manipulation Checks for W1 Manipulations at W2 and across time (W2-W1)

	Control (W1)			Int1: Passive Literacy (W1)			Int2: Active Literacy (W1)			Int3: Inoculation (W1)			
**Variable**	**N**	**Mean**	**SD**	**N**	**Mean**	**SD**	**N**	**Mean**	**SD**	**N**	**Mean**	**SD**	**Test**
Poll Methods Quiz (W2 posttest)	273	0.59	(.32)	269	0.6	(.32)	263	0.67	(.33)	271	0.73	(.31)	F=11.406***
Worried about Misuse of Polls (W2 post)	273	0.49	(.23)	269	0.5	(.23)	263	0.49	(.24)	271	0.54	(.25)	F=3.2**
Informativeness of Social Media Polls (W2 post)	273	0.34	(.23)	269	0.33	(.22)	263	0.35	(.21)	271	0.31	(.22)	F=1.119
General Poll Credibility (W2 post)	273	0.4	(.17)	269	0.41	(.18)	263	0.42	(.17)	271	0.39	(.18)	F=1.616
General Poll Credibility Difference (W2 post – W1 pre)	273	-0.08	(.20)	269	-0.098	(.20)	263	-0.053	(.22)	271	-0.084	(.20)	F=2.166*
General Poll Credibility Difference (W1 post – W1 post)	273	-0.0092	(.15)	269	-0.015	(.16)	263	0.0054	(.17)	271	0.0046	(.17)	F=1.012
General Poll Credibility Index (W2 post)	273	0.55	(.20)	269	0.53	(.22)	263	0.54	(.22)	271	0.58	(.22)	F=2.503*
General Poll Concern Difference (W2 post – W1 pre)	273	0.081	(.23)	269	0.051	(.25)	263	0.048	(.26)	271	0.067	(.25)	F=0.969
General Poll Concern Difference (W2 post – W1 pre)	273	0.049	(.21)	269	0.019	(.19)	263	0.041	(.18)	271	-0.0077	(.18)	F=4.856***
General Poll Efficacy (W2 post)	273	0.46	(.20)	269	0.45	(.18)	263	0.46	(.19)	271	0.48	(.19)	F=0.807
General Poll Efficacy Difference (W2 post – W1 pre)	273	-0.037	(.22)	269	-0.041	(.22)	263	-0.039	(.20)	271	-0.019	(.21)	F=0.593
General Poll Efficacy Index Difference (W2 post – W1 post)	273	-0.013	(.19)	269	-0.014	(.17)	263	-0.0048	(.19)	271	0.011	(.21)	F=1.013
Informed about Baseball (W2 post)	273	0.24	(.25)	269	0.2	(.25)	263	0.19	(.23)	271	0.19	(.23)	F=2.771**
Informed about Baseball (W2 post-W1 post)	261	-0.17	(.26)	255	-0.00098	(.22)	251	-0.019	(.22)	259	-0.02	(.20)	F=31.745***

Statistical significance markers: *** p<0.1; ** p<0.05; *** p<0.01**

Supplementary Material 5: Testing Alternative Models Based on Inclusion of Covariates

A. Results with inclusion of Pre-test Imbalanced Covariates (as reported in the manuscript, with covariate coefficients) [click here to return to TOC]

Control variable coefficients were removed from the manuscript's table due to space limitations; we present them here.

Table 1. Predicting Perceived Credibility of Polls by Interventions and Poll Quality Conditions by Three Modelling Strategies – Full Details Including Control Variable Coefficients

	**Model 0 Baseline Model**	**Analytical Strategy 1**	**Analytical Strategy 2**	**Analytical Strategy 3**			
		**Model 1: Two-way (4 by 2) Interactions**	**Model 2: Eight Conditions**	**Model 3A: Among Control Participants**	**Model 3B: Among Passive Literacy Participants**	**Model 3C: Among Active Literacy Participants**	**Model 3D: Among Inoculation Participants**
Low vs. High Quality Poll (Wave 2)	0.061*** (0.012)	0.021 (0.023)		0.022 (0.024)	0.052* (0.024)	0.050* (0.023)	0.125*** (0.024)
	p = 0.00000	p = 0.369		p = 0.354	p = 0.031	p = 0.033	p = 0.00000
				**[-0.023, 0.069]**	**[0.005, 0.099]**	**[0.004, 0.095]**	**[0.077, 0.172]**
Passive Literacy Intervention (Wave 1)		-0.010 (0.023)					
		p = 0.659					
Active Literacy Intervention (Wave 1)		0.009 (0.024)					
		p = 0.704					
Inoculation Intervention (Wave 1)		-0.050 (0.024) *					
		p = 0.035					
Passive Literacy Intervention X Low vs. High Quality Poll		0.031(0.033)					
		p = 0.344					
Active Literacy Intervention X Low vs. High Quality Poll		0.026 (0.033)					

		p = 0.437					
Inoculation Intervention X Low vs. High Quality Poll		0.102 (0.033) **					
		p = 0.003					
Control Participants Exposed to High Quality Poll			0.021 (0.023)				
			p = 0.369				
Passive Int. Participants Exposed to Low Quality Poll			-0.010 (0.023)				
			p = 0.659				
Passive Literacy Int. Participants Exposed to High Quality Poll			0.042 (0.024)				
			p = 0.084				
Active Literacy Int. Participants Exposed to Low Quality Poll			0.009 (0.024)				
			p = 0.704				
Active Literacy Int. Participants Exposed to High Quality Poll			0.056 (0.024) *				
			p = 0.019				
Inoculation Int. Participants Exposed to Low Quality Poll			-0.050 (0.024) *				
			p = 0.035				
Inoculation Int. Participants Exposed to High Quality Poll			0.072 (0.024) **				
			p = 0.003				
Age	-0.083** (0.028)	-0.085 (0.028) **	-0.085 (0.028) **	-0.008 (0.056)	-0.085 (0.057)	-0.087 (0.055)	-0.157** (0.057)
	p = 0.004	p = 0.003	p = 0.003	p = 0.893	p = 0.135	p = 0.113	p = 0.007
Chinese	-0.054** (0.018)	-0.054 (0.018) **	-0.054 (0.018) **	-0.006 (0.034)	-0.056 (0.040)	-0.086** (0.033)	-0.071 (0.037)
	p = 0.003	p = 0.003	p = 0.003	p = 0.856	p = 0.169	p = 0.009	p = 0.058
Indian	0.070 (0.039)	0.065 (0.039)	0.065 (0.039)	0.078 (0.085)	0.106 (0.088)	-0.006 (0.064)	0.105 (0.082)
	p = 0.075	p = 0.095	p = 0.095	p = 0.365	p = 0.229	p = 0.924	p = 0.205
Poll Interest	0.308*** (0.030)	0.313 (0.030) ***	0.313 (0.030) ***	0.274*** (0.067)	0.341*** (0.057)	0.343*** (0.059)	0.293*** (0.063)

	p = 0.000	p = 0.000	p = 0.000	p = 0.0001	p = 0.000	p = 0.00000	p = 0.00001
Vaccine vs. Artificial Intelligence Issue Polls	-0.027* (0.012)	-0.025 (0.012) *	-0.025 (0.012) *	-0.012 (0.024)	-0.014 (0.024)	-0.054* (0.023)	-0.019 (0.024)
	p = 0.024	p = 0.036	p = 0.036	p = 0.607	p = 0.550	p = 0.022	p = 0.435
Constant	0.300*** (0.028)	0.310 (0.031) ***	0.310 (0.031) ***	0.256*** (0.060)	0.279*** (0.054)	0.345*** (0.051)	0.307*** (0.058)
	p = 0.000	p = 0.000	p = 0.000	p = 0.00003	p = 0.00000	p = 0.000	p = 0.00000
**N**	1,076	1,076	1,076	273	269	263	271
**R^2 / Adjusted R^2**	0.140 / 0.135	0.150 / 0.141	0.150 / 0.141	0.071 / 0.050	0.165 / 0.146	0.188 / 0.169	0.200 / 0.182
**Residual Std. Error**	0.193 (df = 1069)	0.193 (df = 1063)	0.193 (df = 1063)	0.194 (df = 266)	0.195 (df = 262)	0.186 (df = 256)	0.196 (df = 264)
**F Statistic**	29.017*** (df = 6; 1069)	15.653*** (df = 12; 1063), p = 0.000	15.653*** (df = 12; 1063), p = 0.000	3.385** (df = 6; 266)	8.610*** (df = 6; 262)	9.880*** (df = 6; 256)	11.021*** (df = 6; 264)

Notes. The same results are obtained from the data from two different modeling strategies. Model 1 is based on a two-way interaction of two manipulations as predictors, while Model 2 is based on dummy variable testing of eight resultant conditions as predictors. Model 3 provides sub-setted analysis based on W1 arms (control and intervention groups) to allow for direct significance testing for the differences in the effectiveness of interventions through non-parametric bootstrapping. CI for the Low vs. High Quality Polls coefficients in Model3s are bootstrapped confidence intervals. Reference category for Model 1: Control (no intervention) in Wave 1 (W1). The reference category for Model 2: Control participants exposed to low methodological quality polls. The reference category for Model 3 is low methodological quality poll conditions. Stars are used for visual help: *p<0.05; **p<0.01; ***p<0.001; note that exact p-values are also reported in the second rows (under each coefficient). Results control for pretest imbalance variables (age, Chinese, Indian, Interest in Polls) and method factor of issue type (COVID-19 vaccine vs AI risk). The same substantive results are obtained in models without any control variable (SM5) and those that control for additional variables, including exposure to unfavorable poll results (SM5).

Results for Older Pre-registered Models (reported as Model 1 in the final version)

Table 2. Predicting Perceived Credibility of Polls by Methodological Quality, Interventions, and Pre-existing Individual Differences

	**M1:** Quality	**M2:** Quality X Intervene	**M3:** Quality X Intervene X Education	**M4:** Quality X Intervene X Science Literacy	**M5:** Quality X Intervene X Subjective Numeracy	**M6:** Quality X Intervene X News Literacy	**M7:** Quality X Intervene X Unfavoured Result (Vaccine)	**M8:** Quality X Intervene X Unfavoured Result (AI)

**High** Methodological **Quality** (Low Methodological Quality= 0)	0.06***	0.02	0.05	-0.13**	0.04	0.08	0.02	-0.1
	(0.01)	(0.02)	(0.05)	(0.05)	(0.07)	(0.07)	(0.06)	(0.07)
**Passive Literacy** Intervention (W1)		-0.01	0.11*	-0.04	0.001	-0.01	-0.03	-0.1
		(0.02)	(0.05)	(0.05)	(0.07)	(0.07)	(0.06)	(0.07)
**Active Literacy** Intervention (W1)		0.01	0.06	-0.03	0.15	0.004	0.01	-0.11
		(0.02)	(0.05)	(0.05)	(0.08)	(0.07)	(0.06)	(0.08)
**Inoculation** Intervention (W1)		-0.05*	0.01	-0.07	0.05	-0.05	-0.12	-0.13
		(0.02)	(0.05)	(0.05)	(0.07)	(0.06)	(0.07)	(0.07)
**Individual Difference** Moderator (See Column Titles)			0.01	-0.31***	0.05	0.09	-0.13	-0.19
			(0.06)	(0.07)	(0.09)	(0.09)	(0.08)	(0.10)
High Quality X Passive Literacy		0.03	-0.16*	0.12	-0.1	-0.1	0.02	0.1
		(0.03)	(0.07)	(0.07)	(0.10)	(0.09)	(0.09)	(0.11)
High Quality X Active Literacy		0.03	-0.09	0.15*	-0.21*	-0.07	0.03	0.18
		(0.03)	(0.07)	(0.07)	(0.11)	(0.10)	(0.10)	(0.11)
High Quality X Inoculation		0.10**	0.05	0.19**	0.08	0.09	0.19*	0.31**
		(0.03)	(0.07)	(0.07)	(0.10)	(0.09)	(0.10)	(0.10)
High Quality X Individual Difference			-0.06	0.32***	-0.04	-0.11	0.03	0.21
			(0.08)	(0.09)	(0.12)	(0.12)	(0.11)	(0.13)
Passive Literacy X Individual Difference			-0.21**	0.04	-0.02	-0.005	0.07	0.14
			(0.08)	(0.09)	(0.12)	(0.12)	(0.11)	(0.14)
Active Literacy X Individual Difference			-0.1	0.05	-0.24	0.02	0.07	0.17
			(0.08)	(0.10)	(0.13)	(0.13)	(0.12)	(0.14)

Inoculation X Individual Difference			-0.1	0.02	-0.17	0.01	0.16	0.13
			(0.09)	(0.09)	(0.12)	(0.11)	(0.12)	(0.13)
High Quality X Passive Literacy X Individual Difference			0.33**	-0.15	0.25	0.27	-0.04	-0.08
			(0.12)	(0.13)	(0.18)	(0.17)	(0.16)	(0.20)
High Quality X Active Literacy X Individual Difference			0.21	-0.24	0.42*	0.18	-0.05	-0.25
			(0.12)	(0.13)	(0.18)	(0.18)	(0.17)	(0.19)
High Quality X Inoculation X Individual Difference			0.09	-0.18	0.04	0.01	-0.18	-0.40*
			(0.12)	(0.12)	(0.17)	(0.16)	(0.18)	(0.18)
Age	-0.08**	-0.09**	-0.09**	-0.12***	-0.08**	-0.07*	-0.12**	-0.04
	(0.03)	(0.03)	(0.03)	(0.03)	(0.03)	(0.03)	(0.04)	(0.04)
Chinese	-0.05**	-0.05**	-0.05**	-0.03	-0.05**	-0.05**	-0.02	-0.10***
	(0.02)	(0.02)	(0.02)	(0.02)	(0.02)	(0.02)	(0.02)	(0.03)
Indian	0.07	0.07	0.07	0.06	0.06	0.06	0.11	0.01
	(0.04)	(0.04)	(0.04)	(0.04)	(0.04)	(0.04)	(0.06)	(0.05)
Interest in Polls	0.31***	0.31***	0.32***	0.30***	0.31***	0.27***	0.33***	0.31***
	(0.03)	(0.03)	(0.03)	(0.03)	(0.03)	(0.03)	(0.04)	(0.04)
AI Issue (vs. COVID-19 vaccine issue=0)	-0.03*	-0.02*	-0.03*	-0.02	-0.02*	-0.03*		
	(0.01)	(0.01)	(0.01)	(0.01)	(0.01)	(0.01)		
	**M1:**	**M2:**	**M3:**	**M4:**	**M5:**	**M6:**	**M7:**	**M8:**
**Constant**	0.30***	0.31***	0.30***	0.46***	0.28***	0.28***	0.33***	0.42***
	(0.03)	(0.03)	(0.04)	(0.04)	(0.06)	(0.06)	(0.06)	(0.07)
**N**	1,076	1,076	1,076	1,076	1,076	1,076	538	538
**R^2**	0.14	0.15	0.17	0.22	0.17	0.16	0.18	0.16

**F Statistic**	29.02*** (df = 6; 1069)	15.65*** (df = 12; 1063)	10.53*** (df = 20; 1055)	14.63*** (df = 20; 1055)	10.47*** (df = 20; 1055)	10.11*** (df = 20; 1055)	5.94*** (df = 19; 518)	5.21*** (df = 19; 518)

Notes. M denotes model number, Intervene denotes intervention factor, Ind. Differences denotes individual difference moderators and each of them is named in the respective model titles at the top column as the last interaction term in M3 through M8. Bold words in the top 5 row variable names reflect the shortened versions of variable names as used in lower rows. High Methodological Quality contrasts poor (0) and robust (1) quality polls. The reference category for interventions is the control group. *p<0.05; **p<0.01, ***p<.001. Results are persistent when tested among vaccine or AI conditions only and when controlling for poll result directionality within either vaccine or AI conditions, despite halving the sample size in those subgroup tests. Results persisted when demographic control variables were included.

B. Results with No Control/Covariate Variables [click here to return to TOC]

Table. Predicting Perceived Credibility of Polls by Methodological Quality, Interventions, and Pre-existing Individual Differences

	**M1:** Quality	**M2:** Quality X Intervene	**M3**: Quality X Intervene X Education	**M4**: Quality X Intervene X Science Literacy	**M5:** Quality X Intervene X Subjective Numeracy	**M6**: Quality X Intervene X News Literacy	**M7**: Quality X Intervene X Unfavorable (Vaccine)	**M8**: Quality X Intervene X Unfavorable (AI)
Methodological **Quality**	0.06*** (0.01)	0.02 (0.03)	0.1 (0.06)	-0.14** (0.05)	0.08 (0.08)	0.13 (0.07)	0.04 (0.07)	-0.07 (0.08)
**Passive Literacy** Intervention (W1)		-0.01 (0.03)	0.12* (0.05)	-0.05 (0.05)	0.03 (0.07)	0.01 (0.07)	0.03 (0.07)	-0.08 (0.08)
**Active Literacy** Intervention (W1)		0.01 (0.03)	0.09 (0.05)	-0.03 (0.05)	0.18* (0.08)	0.03 (0.07)	0.02 (0.07)	-0.12 (0.09)
**Inoculation** Intervention (W1)		-0.05 (0.03)	0.03 (0.06)	-0.07 (0.05)	0.08 (0.08)	-0.03 (0.07)	-0.13 (0.07)	-0.08 (0.08)
**Individual Difference** Moderator			0.09 (0.06)	-0.34*** (0.07)	0.22* (0.09)	0.27** (0.09)	-0.1 (0.09)	-0.14 (0.11)
Quality X Passive Literacy		0.04 (0.04)	-0.19* (0.08)	0.1 (0.07)	-0.15 (0.11)	-0.11 (0.10)	-0.02 (0.10)	0.13 (0.11)

Quality X Active Literacy		0.02 (0.04)	-0.15 (0.08)	0.14 (0.07)	-0.28* (0.11)	-0.15 (0.10)	0.03 (0.10)	0.21 (0.12)
Quality X Inoculation		0.10** (0.04)	-0.01 (0.08)	0.18* (0.07)	0.02 (0.11)	0.05 (0.10)	0.18 (0.10)	0.25* (0.11)
Quality X Ind. Difference			-0.14 (0.09)	0.32*** (0.10)	-0.1 (0.13)	-0.19 (0.13)	0.01 (0.12)	0.15 (0.15)
Passive Literacy X Ind. Diff.			-0.25** (0.09)	0.04 (0.10)	-0.08 (0.13)	-0.04 (0.12)	-0.04 (0.12)	0.1 (0.15)
Active Literacy X Ind. Diff.			-0.15 (0.09)	0.06 (0.10)	-0.29* (0.14)	-0.02 (0.13)	0.04 (0.13)	0.2 (0.15)
Inoculation X Individual Diff.			-0.15 (0.10)	0.04 (0.10)	-0.23 (0.13)	-0.02 (0.12)	0.18 (0.13)	0.06 (0.14)
Quality X Passive Literacy X Ind. Diff.			0.41** (0.13)	-0.09 (0.14)	0.35 (0.19)	0.3 (0.18)	0.05 (0.17)	-0.12 (0.21)
Quality X Active Literacy X Ind. Diff.			0.32* (0.13)	-0.24 (0.14)	0.54** (0.19)	0.32 (0.18)	-0.05 (0.18)	-0.32 (0.21)
Quality X Inoculation X Ind. Diff.			0.2 (0.13)	-0.17 (0.13)	0.13 (0.18)	0.08 (0.17)	-0.19 (0.19)	-0.3 (0.20)
**Constant**	0.40*** (0.01)	0.41*** (0.02)	0.36*** (0.04)	0.58*** (0.04)	0.29*** (0.06)	0.26*** (0.05)	0.46*** (0.05)	0.48*** (0.06)
**N**	1,076	1,076	1,076	1,076	1,076	1,076	538	538
**R^2**	0.02	0.03	0.04	0.11	0.06	0.08	0.06	0.04
**F Statistic**	22.17*** (df = 1; 1074)	4.62*** (df = 7; 1068)	3.29*** (df = 15; 1060)	8.59*** (df = 15; 1060)	4.76*** (df = 15; 1060)	6.54*** (df = 15; 1060)	2.05* (df = 15; 522)	1.54 (df = 15; 522)

Notes. M denotes model number, Intervene denotes intervention factor, Ind. Differences denotes individual difference moderator and each of them is named in the respective model titles at the top column as the last interaction term in M3 through M8. Bold words in the top 5 row variable names reflect the shortened versions of variable names as used in lower rows. High Methodological Quality contrasts poor (0) and robust (1) quality polls. Reference category

for interventions is the control group. *p<0.05; **p<0.01, ***p<.001. Results are persistent when tested among vaccine or AI conditions only and when controlling for poll result directionality within either vaccine or AI conditions, despite halving the sample size in those subgroup tests. Results persisted when demographic control variables were included.

Figure. Effects on Poll Credibility Perceptions

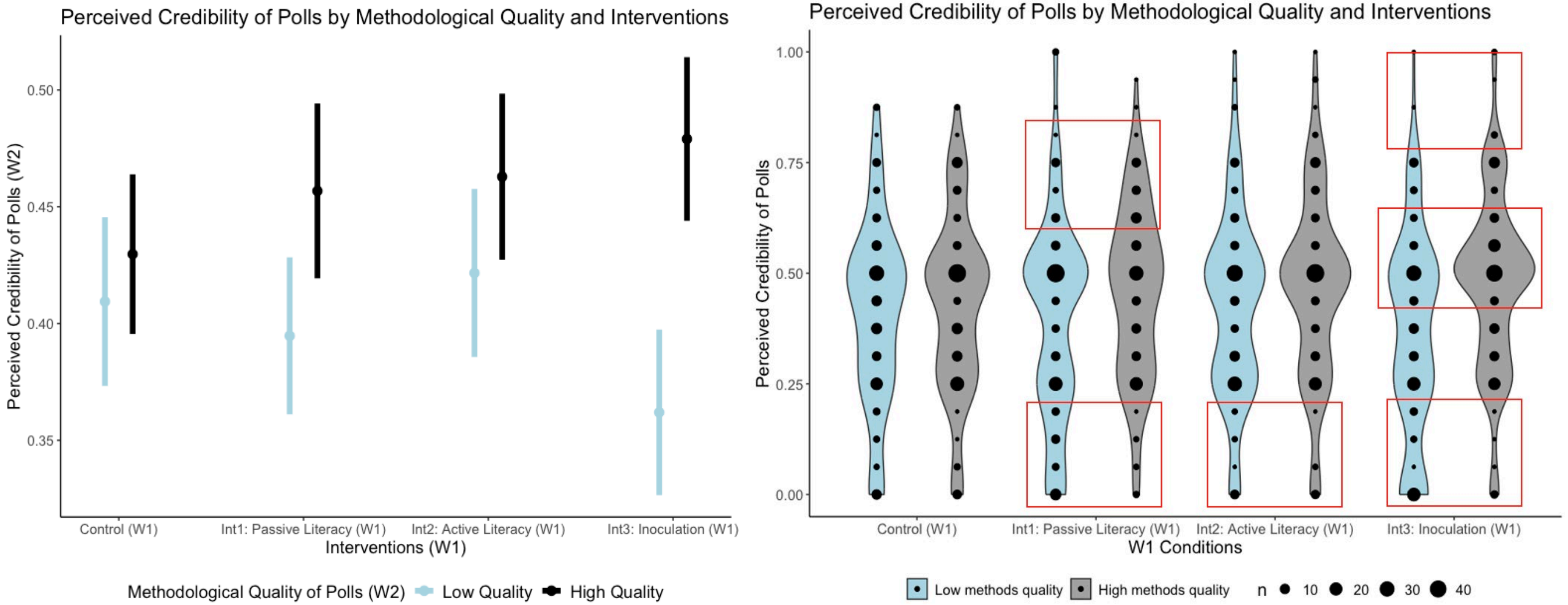

Figure. The Conditional Effects of Interventions on Poll Perceptions by Pre-existing Individual Differences

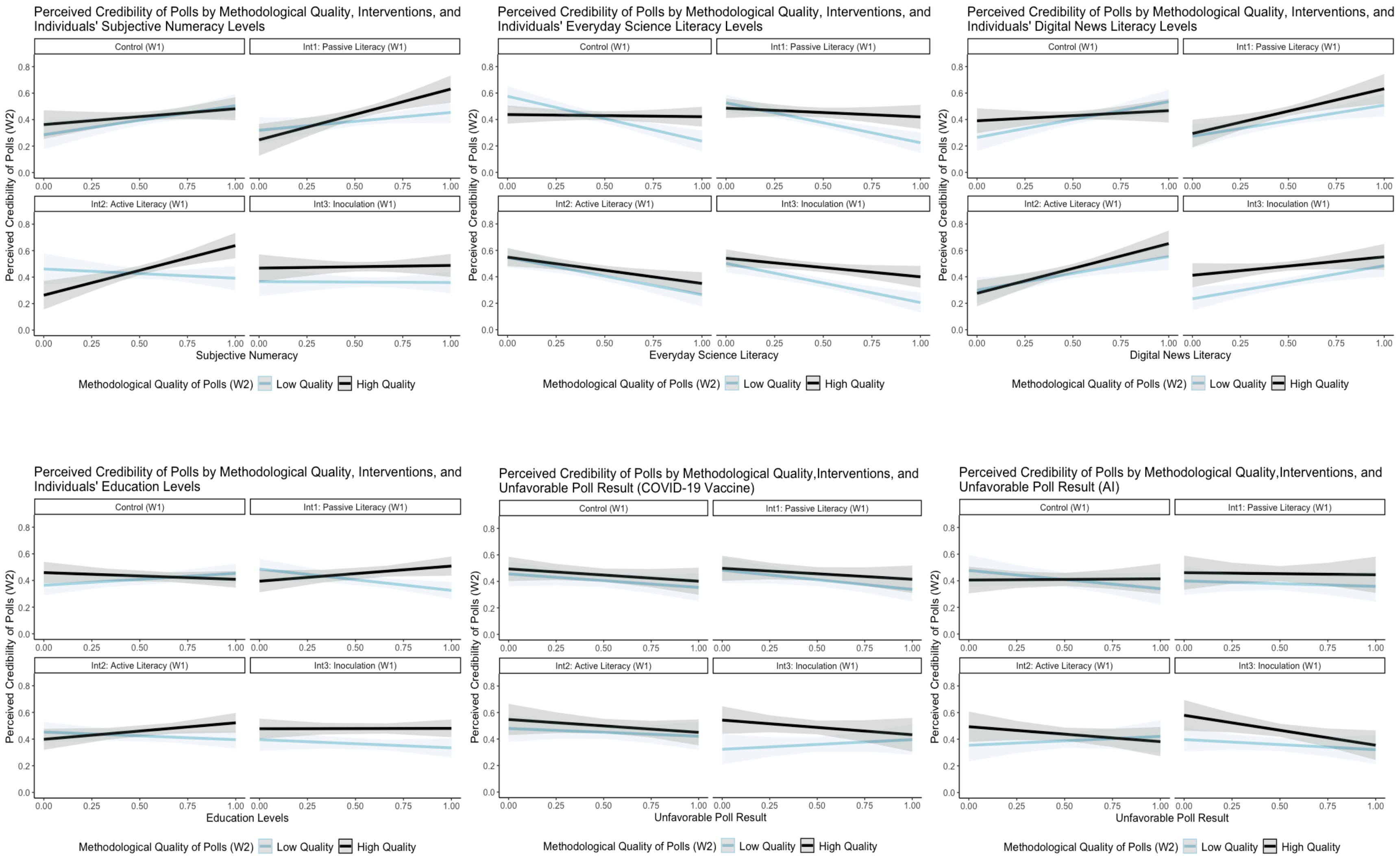

Figure. The Effects of Interventions on Risk and Public Opinion Perceptions

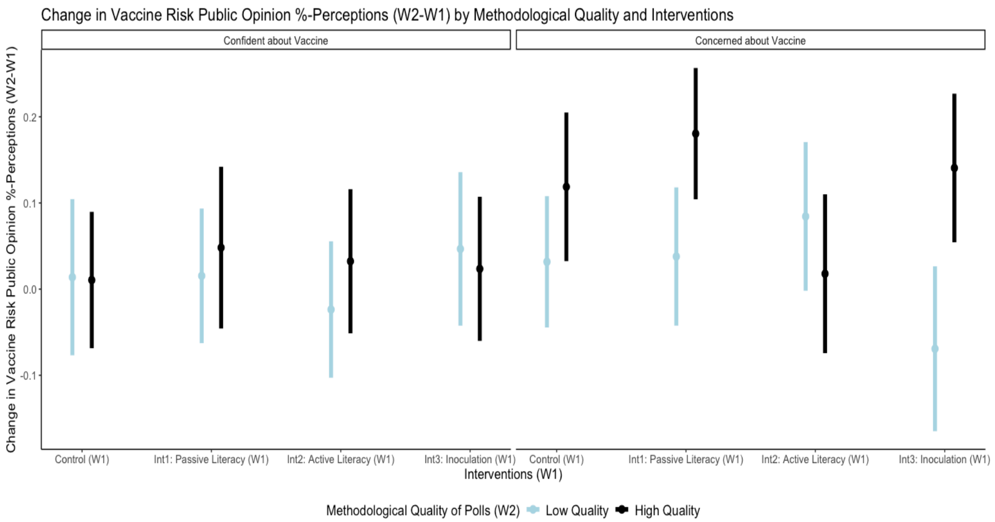

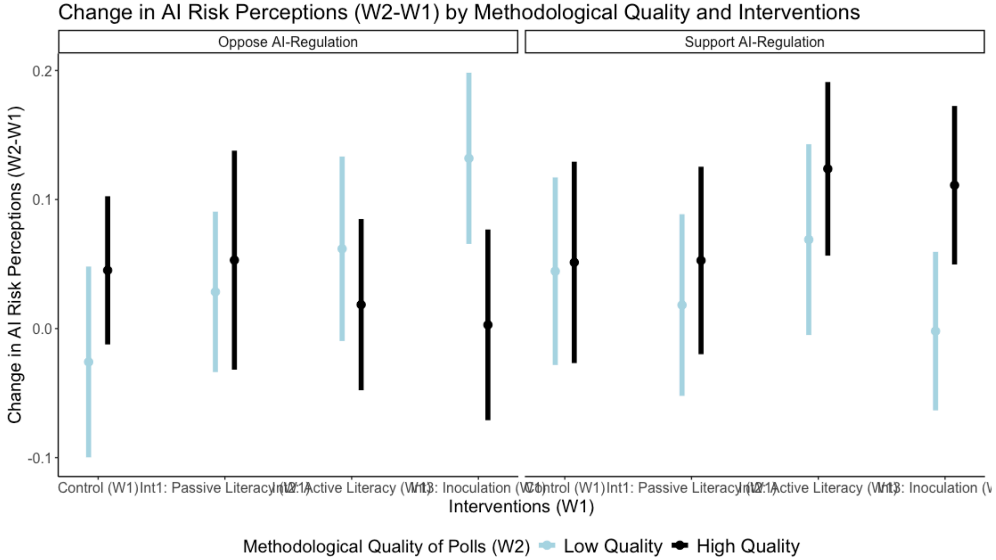

C. Results with All/Additional/Further Theoretically and Methodologically Relevant Control/Covariate Variables [click here to return to TOC]

Table. Results including Further Additional Control Variables (in addition to pre-test imbalance related controls reported in the main paper)

	**Model 1**	**Model 2**	**Model 3: Education**	**Model 4: Science Lit.**	**Model 5: Subj. Num.**	**Model 6: News Lit.**	**Model 7: Unfavorable Vaccine**	**Model 8: Unfavorable AI**
High Methodological Quality	0.06***	0.01	0.03	-0.11*	-0.01	0.03	0.05	-0.1
	(0.01)	(0.02)	(0.05)	(0.05)	(0.07)	(0.06)	(0.06)	(0.07)
Passive Literacy Intervention (W1)		-0.02	0.08	-0.05	-0.02	-0.01	-0.05	-0.07
		(0.02)	(0.05)	(0.04)	(0.06)	(0.06)	(0.06)	(0.07)
Active Literacy Intervention (W1)		0.001	0.05	-0.01	0.1	0.01	0.02	-0.1
		(0.02)	(0.04)	(0.05)	(0.07)	(0.06)	(0.06)	(0.07)
Inoculation Intervention (W1)		-0.04*	-0.02	-0.06	-0.03	-0.04	-0.07	-0.14*
		(0.02)	(0.05)	(0.05)	(0.07)	(0.06)	(0.06)	(0.06)
Unfavorable Result							-0.07	-0.17
							(0.08)	(0.09)
Age	-0.06*	-0.06*	-0.06*	-0.06*	-0.06	-0.06*	-0.08	-0.03
	(0.03)	(0.03)	(0.03)	(0.03)	(0.03)	(0.03)	(0.04)	(0.04)
Sex (Female=1)	-0.01	-0.01	-0.004	-0.01	-0.01	-0.01	-0.01	-0.001
	(0.01)	(0.01)	(0.01)	(0.01)	(0.01)	(0.01)	(0.02)	(0.02)
Chinese	-0.02	-0.02	-0.03	-0.02	-0.03	-0.03	0.01	-0.07
	(0.03)	(0.03)	(0.03)	(0.03)	(0.03)	(0.03)	(0.03)	(0.05)
Malay	0	0.0002	-0.001	-0.005	-0.01	-0.003	0.02	-0.02
	(0.03)	(0.03)	(0.03)	(0.03)	(0.03)	(0.03)	(0.04)	(0.05)
Indian	0.05	0.04	0.04	0.04	0.03	0.04	0.1	-0.01

	(0.04)	(0.04)	(0.04)	(0.04)	(0.04)	(0.04)	(0.06)	(0.06)
Liberal	0.02	0.02	0.01	0.02	0.01	0.02	0.05	-0.02
	(0.03)	(0.03)	(0.03)	(0.03)	(0.03)	(0.03)	(0.04)	(0.04)
Income	0.04	0.04	0.04	0.04	0.04	0.04	0.05	0.03
	(0.02)	(0.02)	(0.02)	(0.02)	(0.02)	(0.02)	(0.04)	(0.04)
Education	-0.03	-0.03	0.04	-0.03	-0.03	-0.03	-0.02	-0.03
	(0.02)	(0.02)	(0.06)	(0.02)	(0.02)	(0.02)	(0.03)	(0.03)
Interest in Polls	0.11**	0.11**	0.11**	0.11**	0.12***	0.11**	0.13*	0.10*
	(0.03)	(0.03)	(0.03)	(0.03)	(0.03)	(0.03)	(0.05)	(0.05)
COVID vs. AI issue polls exposure	-0.03*	-0.03*	-0.03*	-0.02*	-0.02*	-0.03*		
	(0.01)	(0.01)	(0.01)	(0.01)	(0.01)	(0.01)		
Pre-existing Vaccine Risk Perceptions	0.01	0.01	0.01	0.01	0.005	0.01	0.03	-0.01
	(0.03)	(0.03)	(0.03)	(0.03)	(0.03)	(0.03)	(0.04)	(0.04)
Pre-existing AI Risk Perceptions	0.03	0.03	0.03	0.02	0.03	0.03	0.04	-0.005
	(0.03)	(0.03)	(0.03)	(0.03)	(0.03)	(0.03)	(0.04)	(0.04)
Subjective Numeracy	-0.04	-0.04	-0.04	-0.03	-0.03	-0.04	-0.03	-0.06
	(0.03)	(0.03)	(0.03)	(0.03)	(0.08)	(0.03)	(0.05)	(0.05)
News Literacy Behaviors	0.01	0.01	0.01	0.01	0.01	0.02	0.03	-0.03
	(0.03)	(0.03)	(0.03)	(0.03)	(0.03)	(0.08)	(0.05)	(0.05)
Science Literacy	-0.15***	-0.15***	-0.15***	-0.26***	-0.15***	-0.15***	-0.17***	-0.12***
	(0.02)	(0.02)	(0.02)	(0.06)	(0.02)	(0.02)	(0.03)	(0.03)
Trust in Science	0.39***	0.39***	0.39***	0.39***	0.39***	0.40***	0.34***	0.45***
	(0.04)	(0.04)	(0.04)	(0.04)	(0.04)	(0.04)	(0.05)	(0.05)

Traditional News	-0.001	-0.01	-0.004	-0.01	-0.01	-0.01	-0.01	-0.002
	(0.03)	(0.03)	(0.03)	(0.03)	(0.03)	(0.03)	(0.05)	(0.05)
Social Media News	0.02	0.02	0.02	0.02	0.02	0.02	0.01	0.04
	(0.03)	(0.03)	(0.03)	(0.03)	(0.03)	(0.03)	(0.05)	(0.05)
Messaging Application News	0.02	0.02	0.02	0.02	0.02	0.02	0.03	0.02
	(0.03)	(0.03)	(0.03)	(0.03)	(0.03)	(0.03)	(0.04)	(0.04)
Vaccine Side Effect Severity Experienced	-0.03	-0.03	-0.04	-0.04	-0.04	-0.04	-0.04	-0.03
	(0.02)	(0.02)	(0.02)	(0.02)	(0.02)	(0.02)	(0.04)	(0.04)
Experience with AI	-0.01	-0.005	-0.004	-0.003	-0.003	-0.004	-0.03	0.02
	(0.02)	(0.02)	(0.02)	(0.02)	(0.02)	(0.02)	(0.03)	(0.03)
Poll Information Exposure Interference	0.11***	0.11***	0.11***	0.11***	0.11***	0.10***	0.10**	0.10*
	(0.03)	(0.03)	(0.03)	(0.03)	(0.03)	(0.03)	(0.04)	(0.04)
Vaccine Information Exposure Interference	0.03	0.03	0.03	0.03	0.03	0.03	0.03	0.01
	(0.02)	(0.02)	(0.02)	(0.02)	(0.02)	(0.02)	(0.04)	(0.03)
AI Information Exposure Interference	-0.05*	-0.04	-0.04	-0.05	-0.05*	-0.05	-0.08*	0.01
	(0.02)	(0.02)	(0.02)	(0.02)	(0.02)	(0.02)	(0.03)	(0.04)
High Quality X Passive Literacy		0.05	-0.07	0.11	-0.03	-0.06	0.02	0.08
		(0.03)	(0.07)	(0.06)	(0.09)	(0.09)	(0.08)	(0.10)
High Quality X Active Literacy		0.04	-0.04	0.11	-0.11	-0.01	0.02	0.19*
		(0.03)	(0.07)	(0.06)	(0.10)	(0.09)	(0.09)	(0.10)
High Quality X Inoculation		0.10***	0.12	0.16**	0.19*	0.13	0.07	0.32***
		(0.03)	(0.07)	(0.06)	(0.09)	(0.08)	(0.09)	(0.09)

High Quality X Individual Difference			-0.03	0.27^{**}	0.03	-0.03	-0.05	0.23
			(0.08)	(0.08)	(0.11)	(0.11)	(0.11)	(0.12)
Passive Literacy X Individual Difference			-0.20^{**}	0.04	-0.02	-0.03	0.05	0.09
			(0.07)	(0.08)	(0.11)	(0.11)	(0.10)	(0.12)
Active Literacy X Individual Difference			-0.1	0.01	-0.17	-0.02	0.002	0.17
			(0.07)	(0.09)	(0.12)	(0.11)	(0.11)	(0.13)
InoculationX Individual Difference			-0.06	0.03	-0.03	-0.01	0.03	0.21
			(0.08)	(0.08)	(0.11)	(0.10)	(0.12)	(0.12)
High Quality X Passive Literacy X Individual Difference			0.21	-0.12	0.16	0.22	0.07	-0.05
			(0.11)	(0.12)	(0.16)	(0.16)	(0.15)	(0.18)
High Quality X Active Literacy X Individual Difference			0.15	-0.13	0.28	0.11	0.03	-0.28
			(0.11)	(0.12)	(0.16)	(0.16)	(0.16)	(0.17)
High Quality X InoculationX Individual Difference			-0.02	-0.11	-0.15	-0.07	0.04	-0.40^{*}
			(0.11)	(0.12)	(0.16)	(0.15)	(0.17)	(0.17)
**Constant**	0.22^{***}	0.24^{***}	0.21^{***}	0.30^{***}	0.24^{***}	0.23^{***}	0.24^{**}	0.33^{***}
	(0.05)	(0.05)	(0.06)	(0.06)	(0.06)	(0.06)	(0.08)	(0.09)
**Observations**	1,074	1,074	1,074	1,074	1,074	1,074	538	536
**R^2**	0.31	0.32	0.33	0.34	0.33	0.33	0.33	0.36
**F Statistic**	19.25^{***} (df = 25; 1048)	16.15^{***} (df = 31; 1042)	13.56^{***} (df = 38; 1035)	13.87^{***} (df = 38; 1035)	13.67^{***} (df = 38; 1035)	13.32^{***} (df = 38; 1035)	6.48^{***} (df = 38; 499)	7.47^{***} (df = 38; 497)

Notes. *p<0.05; **p<0.01; ***p<0.001

D. Details for RQ1 and RQ2 Results [click here to return to TOC]

Part 1: Results for Vaccine, AI, and Pooled (Vaccine + AI) Separately
Part 2: Testing unfavorable outcome conditional on poll result directionality: split sample

Table. RQ1 and RQ2 models predicting poll credibility (Vaccine Issue)

	Model 1: for RQ1	Model 2: for RQ1 with robust controls	Model 3: for RQ2
Unfavorable Poll (Vaccine)	-0.072*	-0.075*	-0.059
	(0.030)	(0.029)	(0.041)
	p = 0.016	p = 0.012	p = 0.152
Low vs. High Quality Poll		0.061***	0.070*
		(0.017)	(0.033)
		p = 0.0003	p = 0.035
Passive Literacy (W1)		0.008	
		(0.023)	
		p = 0.728	
Active Literacy (W1)		0.049*	
		(0.023)	
		p = 0.038	
Inoculation (W1)		0.008	
		(0.024)	
		p = 0.745	
Age	-0.118**	-0.115**	-0.121**
	(0.040)	(0.040)	(0.040)
	p = 0.004	p = 0.004	p = 0.003
Chinese	-0.027	-0.024	-0.025
	(0.023)	(0.023)	(0.023)
	p = 0.252	p = 0.306	p = 0.277
Indian	0.102	0.096	0.100
	(0.062)	(0.062)	(0.062)
	p = 0.102	p = 0.120	p = 0.105
Poll Interest	0.321***	0.327***	0.322***

	(0.043)	(0.043)	(0.043)
	p = 0.000	p = 0.000	p = 0.000
Unfavorable Poll (Vaccine) X Low vs. High Quality Poll			-0.022
			(0.059)
			p = 0.711
Constant	0.347^{***}	0.296^{***}	0.311^{***}
	(0.039)	(0.042)	(0.042)
	p = 0.000	p = 0.000	p = 0.000
Observations	538	538	538
R^2	0.137	0.166	0.158
Adjusted R^2	0.129	0.152	0.147
Residual Std. Error	0.195 (df = 532)	0.192 (df = 528)	0.193 (df = 530)
F Statistic	16.903^{***} (df = 5; 532)	11.663^{***} (df = 9; 528)	14.186^{***} (df = 7; 530)
Note:			*p<0.05; **p<0.01; ***p<0.001

Table. RQ1 and RQ2 models predicting poll credibility (AI Issue)

	Model 1: for RQ1	Model 2: for RQ1 with robust controls	Model 3: for RQ2
Unfavorable Poll (AI)	-0.052	-0.057	-0.067
	(0.034)	(0.034)	(0.047)
	p = 0.128	p = 0.091	p = 0.154
Low vs. High Quality Poll		0.062^{***}	0.052
		(0.017)	(0.037)
		p = 0.0003	p = 0.160
Passive Literacy (W1)		0.007	
		(0.024)	
		p = 0.757	
Active Literacy (W1)		0.002	
		(0.024)	

		p = 0.935	
Inoculation (W1)		-0.003	
		(0.023)	
		p = 0.911	
Age	-0.027	-0.037	-0.036
	(0.040)	(0.039)	(0.039)
	p = 0.490	p = 0.346	p = 0.355
Chinese	-0.093**	-0.094***	-0.093***
	(0.028)	(0.028)	(0.028)
	p = 0.002	p = 0.001	p = 0.001
Indian	0.032	0.019	0.020
	(0.053)	(0.052)	(0.052)
	p = 0.542	p = 0.718	p = 0.698
Poll Interest	0.285***	0.296***	0.297***
	(0.044)	(0.044)	(0.044)
	p = 0.000	p = 0.000	p = 0.000
Unfavorable Poll (AI) X Low vs. High Quality Poll			0.020
			(0.068)
			p = 0.770
Constant	0.356***	0.324***	0.329***
	(0.042)	(0.045)	(0.044)
	p = 0.000	p = 0.000	p = 0.000
Observations	538	538	538
R^2	0.116	0.138	0.138
Adjusted R^2	0.108	0.123	0.126
Residual Std. Error	0.195 (df = 532)	0.193 (df = 528)	0.193 (df = 530)
F Statistic	13.955*** (df = 5; 532)	9.392*** (df = 9; 528)	12.103*** (df = 7; 530)
Note:			*p<0.05; **p<0.01; ***p<0.001

Table. RQ1 and RQ2 models predicting poll credibility (Vaccine and AI Issues Pooled)

	Model 1: for RQ1	Model 2: for RQ1 with robust controls	Model 3: for RQ2
Unfavorable Poll (Pooled, Vax+AI)	-0.063**	-0.066**	-0.060*
	(0.022)	(0.022)	(0.031)
	p = 0.006	p = 0.003	p = 0.050
Low vs. High Quality Poll		0.061***	0.064**
		(0.012)	(0.025)
		p = 0.00000	p = 0.010
Passive Literacy (W1)		0.009	
		(0.017)	
		p = 0.597	
Active Literacy (W1)		0.026	
		(0.017)	
		p = 0.121	
Inoculation (W1)		0.0004	
		(0.017)	
		p = 0.979	
Age	-0.072*	-0.077**	-0.079**
	(0.028)	(0.028)	(0.028)
	p = 0.011	p = 0.007	p = 0.005
Chinese	-0.057**	-0.055**	-0.056**
	(0.018)	(0.018)	(0.018)
	p = 0.002	p = 0.002	p = 0.002
Indian	0.067	0.056	0.059
	(0.039)	(0.039)	(0.039)
	p = 0.089	p = 0.149	p = 0.133

Poll Interest	0.302***	0.309***	0.307***
	(0.031)	(0.030)	(0.030)
	p = 0.000	p = 0.000	p = 0.000
Unfavorable Poll (Pooled, Vax+AI) X Low vs. High Quality Poll			-0.006
			(0.044)
			p = 0.895
Constant	0.349***	0.308***	0.317***
	(0.029)	(0.031)	(0.030)
	p = 0.000	p = 0.000	p = 0.000
Observations	1,076	1,076	1,076
R^2	0.121	0.145	0.142
Adjusted R^2	0.117	0.138	0.137
Residual Std. Error	0.195 (df = 1070)	0.193 (df = 1066)	0.193 (df = 1068)
F Statistic	29.515*** (df = 5; 1070)	20.082*** (df = 9; 1066)	25.347*** (df = 7; 1068)
Note:			*p<0.05; **p<0.01; ***p<0.001

Part 2: Testing unfavorable outcome conditional on poll result directionality: split sample

Table. Testing unfavorable outcome conditional on poll result directionality: split sample analysis based on issue and result directionality

	Dependent variable:			
	Vaccine (Majority Concerned)	Vaccine (Majority Confident)	AI (Majority Oppose Regulation)	AI (Majority Support Regulation)
Control Seen High Quality	-0.031	0.068	-0.091	-0.005
	(0.104)	(0.076)	(0.103)	(0.104)
	p = 0.769	p = 0.369	p = 0.379	p = 0.964
Interventions Seen Low Quality	0.052	-0.130	-0.160	-0.020
	(0.080)	(0.069)	(0.090)	(0.081)
	p = 0.514	p = 0.061	p = 0.080	p = 0.805

Interventions Seen High Quality	-0.022	0.129	-0.010	0.048
	(0.080)	(0.069)	(0.095)	(0.082)
	p = 0.780	p = 0.063	p = 0.914	p = 0.555
Unfavorable Poll (Vaccine)	-0.148	-0.047		
	(0.107)	(0.145)		
	p = 0.166	p = 0.747		
Unfavorable Poll (AI)			-0.328^{*}	-0.023
			(0.153)	(0.125)
			p = 0.033	p = 0.857
Age	-0.165^{**}	-0.097	-0.105	0.023
	(0.061)	(0.050)	(0.061)	(0.047)
	p = 0.008	p = 0.055	p = 0.087	p = 0.623
Chinese	-0.037	0.003	-0.068	-0.120^{***}
	(0.034)	(0.030)	(0.044)	(0.034)
	p = 0.274	p = 0.917	p = 0.122	p = 0.001
Indian	0.105	0.089	-0.037	0.017
	(0.084)	(0.091)	(0.089)	(0.059)
	p = 0.215	p = 0.330	p = 0.674	p = 0.773
Poll Interest	0.328^{***}	0.293^{***}	0.285^{***}	0.336^{***}
	(0.070)	(0.053)	(0.068)	(0.055)
	p = 0.00001	p = 0.00000	p = 0.00004	p = 0.000
Control Seen High Quality X Unfavorable Poll (Vaccine)	0.144	-0.132		
	(0.166)	(0.176)		
	p = 0.386	p = 0.454		
Interventions Seen Low Quality X Unfavorable Poll (Vaccine)	-0.090	0.242		
	(0.129)	(0.157)		
	p = 0.486	p = 0.124		

Interventions Seen High Quality X Unfavorable Poll (Vaccine)	0.152	-0.101		
	(0.130)	(0.158)		
	p = 0.246	p = 0.524		
Control Seen High Quality X Unfavorable Poll (AI)			0.215	0.051
			(0.205)	(0.174)
			p = 0.297	p = 0.768
Interventions Seen Low Quality X Unfavorable Poll (AI)			0.314	-0.090
			(0.174)	(0.141)
			p = 0.072	p = 0.523
Interventions Seen High Quality X Unfavorable Poll (AI)			0.160	-0.076
			(0.183)	(0.141)
			p = 0.383	p = 0.590
Constant	0.369^{***}	0.303^{***}	0.439^{***}	0.374^{***}
	(0.085)	(0.074)	(0.097)	(0.090)
	p = 0.00003	p = 0.0001	p = 0.00001	p = 0.00005
Observations	269	269	272	266
R^2	0.209	0.211	0.123	0.267
Adjusted R^2	0.176	0.177	0.086	0.235
Residual Std. Error	0.205 (df = 257)	0.172 (df = 257)	0.215 (df = 260)	0.158 (df = 254)
F Statistic	6.187^{***} (df = 11; 257)	6.254^{***} (df = 11; 257)	3.304^{***} (df = 11; 260)	8.416^{***} (df = 11; 254)
Note:				*p<0.05; **p<0.01; ***p<0.001

Supplementary Material 6: Robustness Checks

A. Attrition Analysis [click here to return to TOC]

Demographically, the W2 sample was different from than W1 in several ways as reported in the Table below: Returning respondents were older, more likely to be Chinese race, less likely to be Malay and Indian race, less liberal, and more interested in polls. However, almost all of these variables, and most importantly poll interest, were included as control in our main models.

We note that this is an experiment, and final conditions emerge from the crossing of both W1 and W2 groups, imputation or other statistical methods are not appropriate to examine attrition effects.

We also highlight that attrition is not a big bias problem in an educational context (Valentine & McHugh, 2007).

Most importantly, for experimental purposes, there was no experimental W1 (4 conditions) driven differential attrition. Such that, there was no W1 intervention-induced attrition, and this is important for the validity of the experimental findings. For example, participants in any intervention group were not more or less likely than the control (as well as, other interventions) to participate in the W2 survey. See cell balances in equivalence of conditions related to supplementary material.

Table. Attrition Comparison

	W1 Attrition Respondents who did not turn out in Wave 2			W2 (Returning) Respondents (Longitudinal Analysis Sample)			
**Pre-test Variables at W1**	**N**	**Mean**	**SD**	**N**	**Mean**	**SD**	**Test**
Age	951	.33	.21	1076	.37	.21	F=19.432***
Sex (Female=1)	951			1076			
Chinese Race	951	.79	.41	1076	.84	.36	F=10.382***
Malay Race	951	.11	.31	1076	.08	.27	F=3.859**
Indian Race	951	.06	.24	1076	.03	.16	F=14.116***

Other Race	951	.04	.20	1076	.05	.21	F=0.214
Liberal	951	.51	.20	1076	.50	.19	F=3.037*
Income	951	.38	.23	1076	.38	.24	F=0.065
Education	951	.53	.27	1076	.55	.28	F=1.614
Poll Interest Index	951	.54	.20	1076	.57	.19	F=10.826***
Polls are Credible in General	951	.47	.18	1076	.48	.19	F=2.324
Concerned about Polls	951	.52	.22	1076	.50	.22	F=3.82*
Efficacious about Polls	951	.48	.20	1076	.50	.19	F=7.829***
COVID-19 Vaccine Risk Perceptions	951	.43	.25	1076	.44	.24	F=1.004
Public Opinion Perceptions about COVID-19 Vaccine Risk (Verbal)	951	.55	.26	1075	.54	.26	F=0.01
Public Opinion Perceptions about COVID-19 Vaccine Risk (Percentage)	951	.39	.27	1076	.39	.27	F=0.004
COVID-19 Vaccine (Booster) Intention	951	.53	.31	1076	.54	.29	F=0.098
COVID-19 Vaccine Overall Attitude (Positivity)	951	.59	.25	1076	.59	.24	F=0.005
AI Risk Perceptions	951	.42	.22	1076	.42	.22	F=0.18
Public Opinion Perceptions about AI Risk (Verbal)	948	.54	.26	1073	.52	.26	F=1.302
Public Opinion Perceptions about AI Risk (Percentage)	951	.38	.25	1075	.38	.24	F=0.011
AI Regulation Policy View	951	.66	.23	1076	.68	.22	F=2.33
AI Overall Attitude (Positivity)	951	.61	.21	1076	.61	.20	F=0.127
News Literacy Behaviors	951	.51	.20	1076	.50	.20	F=1.059
Subjective Numeracy	951	.56	.19	1076	.56	.19	F=0.07
Everyday Science Literacy	951	.45	.25	1076	.45	.25	F=0.127

Statistical significance markers: *** p<0.1; ** p<0.05; *** p<0.01**

B. Longitudinal Interference Checks [click here to return to TOC]

Did individuals' exposure to information that is relevant to our manipulations between W1 and W2 surveys shape our findings? To provide additional insights into the longitudinal analysis with greater ecological validity (observing effects of real-world exposure to information that might suppress, facilitate, or condition the experimental effects), we measured for and tested a few interference factors. Asking about the type of content goes beyond asking and controlling for media/news exposure questions because these questions specifically tap into content related to our experiment:

1. Exposure to any type of COVID-19 vaccine-related information and news
   a. Main effect: There is a positive effect of being exposed to vaccine-related information on the perceived credibility of polls in general (b=.10, p<.001) as well as only among vaccine-related polls (b=.10, p<.01). The inclusion of this variable does not change results.
   b. Moderation effect: This interference measure does not moderate the experimental effects.
2. Exposure to any type of AI-related information and news
   a. Main effect: There is a positive effect of being exposed to AI-related information on the perceived credibility of polls in general (b=.06, p<.01) as well as only among AI-related polls (b=.12, p<.001). The inclusion of this variable does not change results.
   b. Moderation effect: This interference measure moderates the effects of active literacy intervention (b=.37, p<.05). For respondents in the active literacy training intervention, greater exposure to AI-related information and news during the time from W1 to W2 was associated with greater effectiveness of the intervention, such that, respondents distinguished low and high-quality polls more strongly. The same trend is visible for the other two interventions (although they are not significant) while no such trend is visible for the control condition. This strongly suggests that training received in any of the interventions (particularly active literacy intervention) conditioned respondents' engagement with AI-related information and news afterward in some way, which subsequently resulted in a greater exhibition of intervention effects for those who were exposed to more AI-related information. This effect is visualized in the plot further below.
3. Exposure to poll reports/findings in general/on any topic
   a. Main effect: There is a positive effect of being exposed to AI-related information on the perceived credibility of polls in general (b=.16, p<.001). The inclusion of this variable does not change results.
   b. Moderation effect: This interference measure does not moderate the experimental effects.
4. **Overall conclusion**: Collectively, these results reveal that there was almost no methodological bias that would have influenced our main findings as a confound. The main effects observed for all three interference measures provide further evidence that in between the two waves' data collection, respondents' real-world daily engagement with vaccine or AI-related information and news as well as any poll exposure have played some role in their credibility evaluations of polls at W2. This might suggest that those respondents who were more

involved with these issues or polls were more attentive in general. These results also show the importance of our "all control variables included" models elsewhere in this Supplementary Materials, since we replicate the main findings by controlling for these interference measures. Interference measures cannot be part of pre-test balance tests, but they can retroactively provide an opportunity to control for the content of information exposure between the waves that are above and beyond media and news exposure questions.

Table. Main and Interaction Effects of Interference Measures

	**Vaccine Interference**				**AI Interference**				**Poll Interference**	
	Among All Polls		Among Vaccine Polls		Among All Polls		Among AI Polls		Among All Polls	
	Main Effects	Interact. Effects	Main Effects	Interact. Effects	Main Effects	Interact. Effects	Main Effects	Interact. Effects	Main Effects	Interact. Effects
High Methodological Quality (Low Methodological Quality= 0)	0.02	0.01	0.02	0.04	0.02	0.05	0.01	0.03	0.02	0.04
	(0.02)	(0.03)	(0.03)	(0.05)	(0.02)	(0.03)	(0.03)	(0.05)	(0.02)	(0.03)
Passive Literacy Intervention (W1)	-0.01	0.004	-0.01	-0.02	-0.01	0.01	-0.02	0.06	-0.005	0.01
	(0.02)	(0.03)	(0.03)	(0.04)	(0.02)	(0.03)	(0.03)	(0.05)	(0.02)	(0.03)
Active Literacy Intervention (W1)	0.01	0.06	0.04	0.06	0.02	0.05	-0.02	0.08	0.01	0.04
	(0.02)	(0.03)	(0.03)	(0.04)	(0.02)	(0.03)	(0.04)	(0.05)	(0.02)	(0.03)
Inoculation Intervention (W1)	-0.05^{*}	-0.02	-0.05	-0.04	-0.05^{*}	-0.01	-0.06	0.02	-0.05^{*}	-0.01
	(0.02)	(0.03)	(0.03)	(0.05)	(0.02)	(0.04)	(0.03)	(0.05)	(0.02)	(0.03)
Interference Measure (See Column Names)	0.10^{***}	0.16^{**}	0.10^{**}	0.20^{*}	0.06^{**}	0.14^{*}	0.12^{***}	0.28^{**}	0.16^{***}	0.25^{***}
	(0.02)	(0.05)	(0.03)	(0.09)	(0.02)	(0.06)	(0.03)	(0.08)	(0.03)	(0.06)
Age	-0.10^{***}	-0.10^{***}	-0.13^{**}	-0.13^{***}	-0.09^{**}	-0.08^{**}	-0.05	-0.04	-0.06^{*}	-0.06^{*}
	(0.03)	(0.03)	(0.04)	(0.04)	(0.03)	(0.03)	(0.04)	(0.04)	(0.03)	(0.03)
Chinese	-0.06^{**}	-0.06^{**}	-0.02	-0.02	-0.05^{**}	-0.06^{**}	-0.10^{***}	-0.10^{***}	-0.05^{**}	-0.06^{**}
	(0.02)	(0.02)	(0.02)	(0.02)	(0.02)	(0.02)	(0.03)	(0.03)	(0.02)	(0.02)
Indian	0.07	0.07	0.1	0.09	0.07	0.07	0.03	0.03	0.06	0.06

	(0.04)	(0.04)	(0.06)	(0.06)	(0.04)	(0.04)	(0.05)	(0.05)	(0.04)	(0.04)
Interest in Polls	0.28***	0.28***	0.30***	0.29***	0.28***	0.28***	0.23***	0.24***	0.23***	0.23***
	(0.03)	(0.03)	(0.04)	(0.04)	(0.03)	(0.03)	(0.05)	(0.05)	(0.03)	(0.03)
AI Issue (vs. COVID-19 vaccine issue=0)	-0.03**	-0.03**			-0.02	-0.02			-0.03*	-0.03*
	(0.01)	(0.01)			(0.01)	(0.01)			(0.01)	(0.01)
High Quality X Passive Literacy	0.03	0.04	0.02	0.03	0.03	-0.02	0.06	-0.01	0.02	0.02
	(0.03)	(0.05)	(0.05)	(0.07)	(0.03)	(0.05)	(0.05)	(0.07)	(0.03)	(0.04)
High Quality X Active Literacy	0.02	0.003	0.01	0.03	0.02	-0.03	0.04	-0.04	0.02	0.01
	(0.03)	(0.05)	(0.05)	(0.06)	(0.03)	(0.05)	(0.05)	(0.07)	(0.03)	(0.04)
High Quality X Inoculation	0.10**	0.11*	0.11*	0.15*	0.10**	0.07	0.11*	0.04	0.10**	0.07
	(0.03)	(0.05)	(0.05)	(0.07)	(0.03)	(0.05)	(0.05)	(0.07)	(0.03)	(0.04)
High Quality X IInterference Measure		0.01		-0.09		-0.08		-0.07		-0.07
		(0.08)		(0.12)		(0.08)		(0.12)		(0.09)
Passive Literacy X Interference Measure		-0.06		0.01		-0.07		-0.25*		-0.04
		(0.07)		(0.12)		(0.08)		(0.11)		(0.09)
Active Literacy X Interference Measure		-0.17*		-0.09		-0.12		-0.40**		-0.1
		(0.08)		(0.13)		(0.08)		(0.13)		(0.09)
Inoculation X Interference Measure		-0.1		-0.04		-0.13		-0.23*		-0.19*
		(0.08)		(0.14)		(0.08)		(0.11)		(0.09)
High Quality X Passive Literacy X Interference Measure		0.001		-0.01		0.16		0.22		0.01
		(0.11)		(0.16)		(0.11)		(0.17)		(0.13)
High Quality X Active Literacy X Interference Measure		0.09		-0.04		0.16		0.37*		0.07

		(0.11)		(0.17)		(0.11)		(0.17)		(0.13)
High Quality X Inoculation X Interference Measure		-0.03		-0.12		0.08		0.21		0.11
		(0.11)		(0.18)		(0.11)		(0.16)		(0.13)
Constant	0.31^{***}	0.29^{***}	0.27^{***}	0.26^{***}	0.30^{***}	0.28^{***}	0.34^{***}	0.28^{***}	0.31^{***}	0.30^{***}
	(0.03)	(0.03)	(0.04)	(0.05)	(0.03)	(0.04)	(0.05)	(0.05)	(0.03)	(0.03)
Observations	1,076	1,076	538	538	1,076	1,076	538	538	1,076	1,076
R^2	0.17	0.17	0.18	0.2	0.16	0.16	0.17	0.19	0.18	0.19
F Statistic	16.51^{***} (df = 13; 1062)	11.13^{***} (df = 20; 1055)	9.80^{***} (df = 12; 525)	6.76^{***} (df = 19; 518)	15.28^{***} (df = 13; 1062)	10.25^{***} (df = 20; 1055)	8.77^{***} (df = 12; 525)	6.42^{***} (df = 19; 518)	18.22^{***} (df = 13; 1062)	12.15^{***} (df = 20; 1055)

*p<0.05; **p<0.01, ***p<.001.

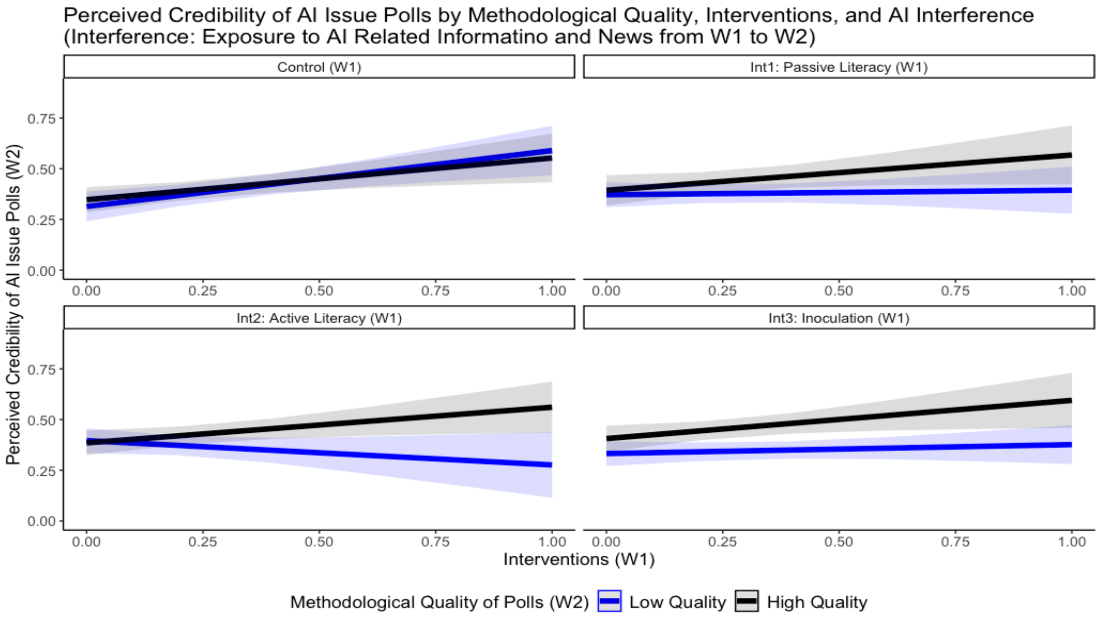

Supplementary Material 7: Other Preregistered and Exploratory Analyses

A. Shifts in General Evaluations of Polls across Time [click here to return to TOC]

1. Did general evaluations of polls in general shift overall from W1 to W2?
   a. Yes, there was a significant decrease in general credibility evaluations of polls (M_{diff}= -.08, t=-12.58, p< .001), a significant increase in concern about polls (M_{diff}= .06, t=8.13, p< .001), and a significant decrease (M_{diff}= -.03, t=-5.28, p< .001) in efficacy about polls in general.
2. Did general evaluations of polls change as a result of interventions? (Interventions as a predictor of evaluations)
   a. No. Intereventions did not predict their general poll evaluations. Also, the above t-tests for change scores for the three measures (credibility, concern, efficacy) were significant among control (no intervention) subjects too.
3. Did general evaluations of polls (pre-test pre-existing scores) moderate the effects of interventions?
   a. No. There was no significant interaction, plots are shown below. None of these were significant.
4. Conclusions:
   a. While polling evaluations, in general, shifted slightly to be more negative among returning subjects (N=1076 W2 respondents) compared to W1 (even among control – no treatment subjects), these general evaluations were not influenced by interventions, and pre-test pre-existing evaluations about polls did not moderate the effects of interventions. One worry was the trainings could induce general skepticism as seen in other contexts (Guess et al., 2020), but given these findings, it is inconclusive and not substantive. Even if there is some healthy dose of skepticism, it might be worth for more critical evaluation of specific polls that individuals encounter.

Table. Did Interventions Change General Poll Evaluations

	Credibility (W2)	Credibility (W2-W1)	Concern (W2)	Concern (W2-W1)	Efficacy (W2)	Efficacy (W2-W1)
High Methodological Quality	0.003	0.02	-0.02	0.02	-0.002	-0.001
	(0.02)	(0.02)	(0.02)	(0.03)	(0.02)	(0.03)
Passive Literacy Intervention	0.01	0.01	-0.004	-0.004	-0.01	-0.003
	(0.02)	(0.02)	(0.02)	(0.03)	(0.02)	(0.03)
Active Literacy Intervention	0.02	0.02	-0.003	-0.005	-0.01	-0.01

	(0.02)	(0.02)	(0.03)	(0.03)	(0.02)	(0.03)
Inoculation Intervention	-0.02	-0.001	0.02	-0.01	0.02	-0.002
	(0.02)	(0.02)	(0.03)	(0.03)	(0.02)	(0.03)
Age	-0.08**	-0.06*	-0.01	0.11**	-0.12***	-0.03
	(0.02)	(0.03)	(0.03)	(0.04)	(0.03)	(0.03)
Chinese	-0.02	0.02	-0.05*	-0.05*	-0.05**	-0.002
	(0.02)	(0.02)	(0.02)	(0.02)	(0.02)	(0.02)
Indian	0.08*	0.04	-0.03	-0.02	0.06	0.06
	(0.03)	(0.04)	(0.04)	(0.05)	(0.04)	(0.04)
Interest in Polls	0.27***	-0.30***	0.27***	0.14***	0.32***	-0.17***
	(0.03)	(0.03)	(0.03)	(0.04)	(0.03)	(0.03)
High Methodological Quality X Passive Literacy Intervention	-0.01	-0.05	-0.02	-0.05	-0.01	-0.001
	(0.03)	(0.03)	(0.04)	(0.04)	(0.03)	(0.04)
High Methodological Quality X Active Literacy Intervention	0.0001	0.01	-0.01	-0.05	0.02	0.002
	(0.03)	(0.03)	(0.04)	(0.04)	(0.03)	(0.04)
High Methodological Quality X Inoculation Intervention	0.02	-0.01	0.02	-0.004	-0.004	0.04
	(0.03)	(0.03)	(0.04)	(0.04)	(0.03)	(0.04)
Constant	0.30***	0.08**	0.45***	-0.01	0.36***	0.07*
	(0.03)	(0.03)	(0.03)	(0.04)	(0.03)	(0.03)
N	1,076	1,076	1,076	1,076	1,076	1,076
R^2	0.12	0.09	0.08	0.03	0.16	0.03

F Statistic (df = 11; 1064)	13.06***	9.73***	8.14***	2.92***	18.52***	2.91***

*p<0.05; **p<0.01; ***p<0.001

Table: Did Pretest General Poll Evaluations Moderate Intervention Effects?

	General Credibility as Moderator	General Efficacy as Moderator	General Concern as Moderator
High Methodological Quality	-0.06	-0.05	0.04
	(0.06)	(0.06)	(0.06)
Passive Literacy Intervention	-0.03	-0.01	0
	(0.06)	(0.06)	(0.06)
Active Literacy Intervention	-0.05	-0.03	-0.01
	(0.06)	(0.07)	(0.06)
Inoculation Intervention	-0.13*	-0.02	-0.04
	(0.06)	(0.06)	(0.06)
General Poll Evaluation Moderator (See Column Title)	0.26**	0.1	0.002
	(0.08)	(0.08)	(0.07)
Age	-0.08**	-0.07**	-0.09**
	(0.03)	(0.03)	(0.03)
Chinese	-0.04*	-0.05**	-0.05**
	(0.02)	(0.02)	(0.02)
Indian	0.05	0.07	0.07
	(0.04)	(0.04)	(0.04)
Interest in Polls	0.10**	0.25***	0.32***

	(0.04)	(0.03)	(0.03)
Vaccine vs. AI Issue	-0.02	-0.02*	-0.03*
	(0.01)	(0.01)	(0.01)
High Methodological Quality X Passive Literacy Intervention	0.04	0.06	0.01
	(0.09)	(0.09)	(0.08)
High Methodological Quality X Active Literacy Intervention	0.12	0.1	0.11
	(0.09)	(0.09)	(0.08)
High Methodological Quality X Inoculation Intervention	0.19*	0.07	0.12
	(0.09)	(0.09)	(0.08)
High Methodological Quality X Moderator (See Column Title)	0.18	0.14	-0.04
	(0.12)	(0.12)	(0.10)
Passive Literacy Intervention X Moderator (See Column Title)	0.04	-0.01	-0.02
	(0.11)	(0.11)	(0.10)
Active Literacy Intervention X Moderator (See Column Title)	0.13	0.07	0.03
	(0.12)	(0.12)	(0.11)
Inoculation Intervention X Moderator (See Column Title)	0.16	-0.06	-0.02
	(0.11)	(0.11)	(0.10)
High Methodological Quality X Passive Literacy Intervention X Moderator (See Column Title)	-0.03	-0.06	0.05
	(0.17)	(0.17)	(0.15)
High Methodological Quality X Active Literacy Intervention X Moderator (See Column Title)	-0.19	-0.15	-0.17
	(0.17)	(0.17)	(0.15)

High Methodological Quality X Inoculation Intervention X Moderator (See Column Title)	-0.16<br>(0.17)	0.08<br>(0.17)	-0.04<br>(0.15)
Constant	0.29*** (0.05)	0.29*** (0.05)	0.31*** (0.05)
N	1,076	1,076	1,076
R^2	0.23	0.17	0.16
F Statistic (df = 20; 1055)	15.93***	10.86***	9.76***

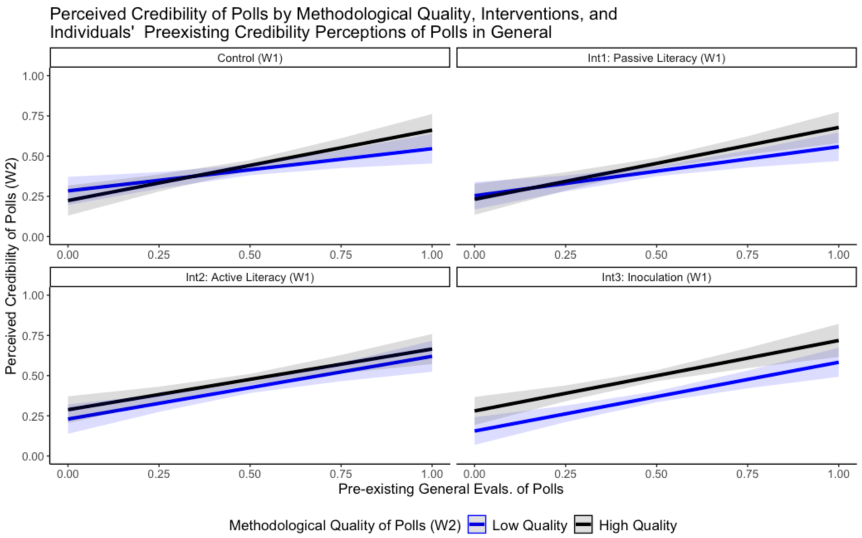
Perceived Credibility of Polls by Methodological Quality, Interventions, and Individuals' Preexisting Credibility Perceptions of Polls in General
Control (W1)
Int1: Passive Literacy (W1)
Int2: Active Literacy (W1)
Int3: Inoculation (W1)
Perceived Credibility of Polls (W2)
Pre-existing General Evals. of Polls
Methodological Quality of Polls (W2)
Low Quality
High Quality

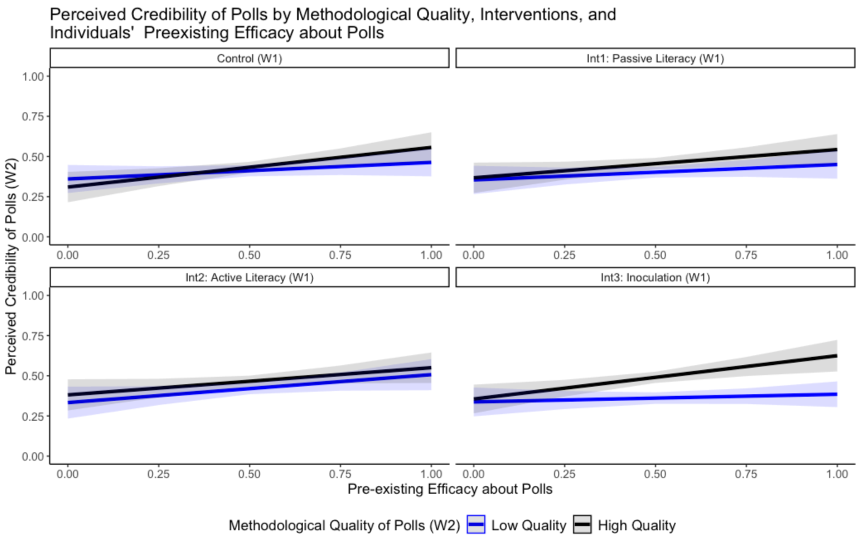
Perceived Credibility of Polls by Methodological Quality, Interventions, and Individuals' Preexisting Efficacy about Polls
Control (W1)
Int1: Passive Literacy (W1)
Int2: Active Literacy (W1)
Int3: Inoculation (W1)
Perceived Credibility of Polls (W2)
Pre-existing Efficacy about Polls
Methodological Quality of Polls (W2)
Low Quality
High Quality

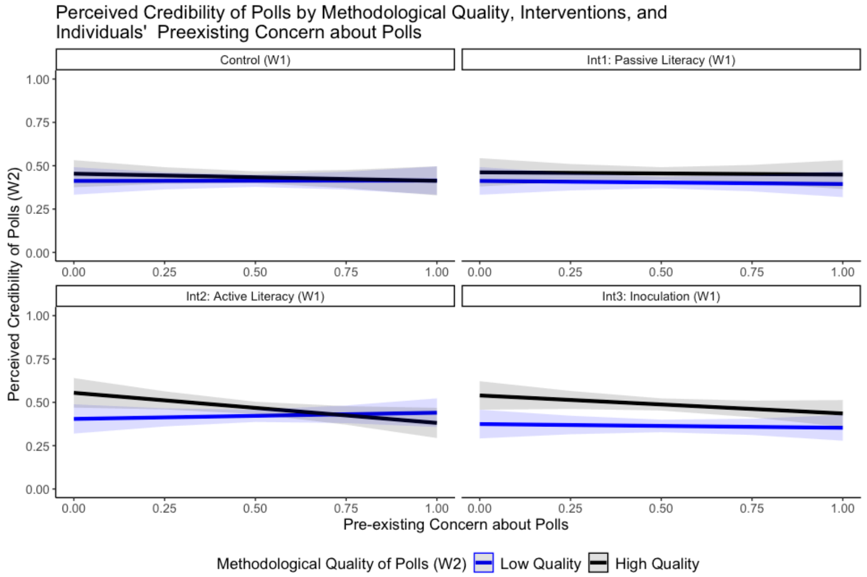
Perceived Credibility of Polls by Methodological Quality, Interventions, and Individuals' Preexisting Concern about Polls
Control (W1)
Int1: Passive Literacy (W1)
Int2: Active Literacy (W1)
Int3: Inoculation (W1)
Perceived Credibility of Polls (W2)
Pre-existing Concern about Polls
Methodological Quality of Polls (W2)
Low Quality
High Quality

References for Supplementary Materials [click here to return to TOC]

Bailey, M. A. (2024). *Polling at a Crossroads: Rethinking Modern Survey Research*. Cambridge University Press. https://books.google.co.th/books?hl=th&lr=&id=G3n1EAAAQBAJ&oi=fnd&pg=PR12&dq=Polling+at+a+Crossroads&ots=6Hx9bI7-lj&sig=pl6-kU5k9sbmSnAebo3fGRTFMcQ&redir_esc=y#v=onepage&q=Polling%20at%20a%20Crossroads&f=false

Baker, R., Blumberg, S. J., Brick, J. M., Couper, M. P., Courtright, M., Dennis, J. M., Dillman, D., Frankel, M. R., Garland, P., Groves, R. M., Kennedy, C., Krosnick, J., Lavrakas, P. J., Lee, S., Link, M., Piekarski, L., Rao, K., Thomas, R. K., & Zahs, D. (2010). Research Synthesis: AAPOR Report on Online Panels. *Public Opinion Quarterly*, *74*(4), 711–781. https://doi.org/10.1093/poq/nfq048

Basol, M., Roozenbeek, J., & van der Linden, S. (2020). Good News about Bad News: Gamified Inoculation Boosts Confidence and Cognitive Immunity Against Fake News. *Journal of Cognition*, *3*(1), 2. https://doi.org/10.5334/joc.91

Blendi & respondi. (2025). *Blendi & respondi - Services for the Market Research* . https://www.bilendi.co.uk/static/studymarket#sm-sec-15

Bradley, V. C., Kuriwaki, S., Isakov, M., Sejdinovic, D., Meng, X. L., & Flaxman, S. (2021). Unrepresentative big surveys significantly overestimated US vaccine uptake. *Nature*, *600*(7890), 695–700. https://doi.org/10.1038/s41586-021-04198-4

Bubela, T., Nisbet, M. C., Borchelt, R., Brunger, F., Critchley, C., Einsiedel, E., Gellerg, G., Guptal, A., Hampel, J., Hyde-Lay, R., Jandciu, E. W., Jones, A., Kolopack, P., Lane, S., Lougheed, T., Nerlich, B., Ogbogu, U., O'Riordan, K., Ouellette, C., … Caulfield, T. (2009). Science communication reconsidered. *Nature Biotechnology*, *27*(6), 514–519. https://go-gale-com.libproxy1.nus.edu.sg/ps/i.do?p=AONE&sw=w&issn=10870156&v=2.1&it=r&id=GALE%7CA201944223&sid=googleScholar&linkaccess=fulltext

Capewell, G., Maertens, R., Linden, Dr. S. van der, & Roozenbeek, J. (2023). *Misinformation interventions decay rapidly without an immediate post-test*. https://doi.org/10.31234/OSF.IO/93UJX

Chan, M. S., Jones, C. R., Jamieson, K. H., & Albarracín, D. (2017). Debunking: A Meta-Analysis of the Psychological Efficacy of Messages Countering Misinformation. *Psychological Science*, *28*(11), 1531–1546. https://doi.org/10.1177/0956797617714579

Collins, L. M., Dziak, J. J., Kugler, K. C., & Trail, J. B. (2014). Factorial experiments: Efficient tools for evaluation of intervention components. *American Journal of Preventive Medicine*, *47*(4), 498–504. https://doi.org/10.1016/j.amepre.2014.06.021

Collins, L. M., Dziak, J. J., & Li, R. (2009). Design of Experiments With Multiple Independent Variables: A Resource Management Perspective on Complete and Reduced Factorial Designs. *Psychological Methods*, *14*(3), 202–224. https://doi.org/10.1037/a0015826

Collins, L. M., Nahum-Shani, I., Guastaferro, K., Strayhorn, J. C., Vanness, D. J., & Murphy, S. A. (2024). Intervention Optimization: A Paradigm Shift and Its Potential Implications for Clinical Psychology. *Annual Review of Clinical Psychology*, *20*(1), 21–47. https://doi.org/10.1146/annurev-clinpsy-080822-051119

Comprehensive Labour Force Survey. (2022). Ministry of Manpower. https://www.mom.gov.sg/newsroom/press-releases/2022/0425-launch-of-clfs-2022

Compton, J. (2021). Threat and/in Inoculation Theory. *International Journal of Communication*, *15*(0), 13. https://ijoc.org/index.php/ijoc/article/view/17634

de Saint Laurent, C., Murphy, G., Hegarty, K., & Greene, C. M. (2022). Measuring the effects of misinformation exposure and beliefs on behavioural intentions: a COVID-19 vaccination study. *Cognitive Research: Principles and Implications 2022 7:1*, *7*(1), 1–19. https://doi.org/10.1186/S41235-022-00437-Y

Department of Statistics. (2020). *Singapore Census of Population 2020, Statistical Release 1: Demographic Characteristics, Education, Language and Religion*. Department of Statistics, Singapore. https://www.singstat.gov.sg/publications/reference/cop2020/cop2020-sr1/census20_stat_release1

Dziak, J. J., Nahum-Shani, I., & Collins, L. M. (2012). Multilevel factorial experiments for developing behavioral interventions: Power, sample size, and resource considerations. *Psychological Methods*, *17*(2), 153–175. https://doi.org/10.1037/a0026972

Green, M., McShane, C. J., & Swinbourne, A. (2022). Active versus passive: evaluating the effectiveness of inoculation techniques in relation to misinformation about climate change. *Australian Journal of Psychology*, *74*(1). https://doi.org/10.1080/00049530.2022.2113340

Guess, A. M., Lerner, M., Lyons, B., Montgomery, J. M., Nyhan, B., Reifler, J., & Sircar, N. (2020). A digital media literacy intervention increases discernment between mainstream and false news in the United States and India. *Proceedings of the National Academy of Sciences*, *117*(27), 15536–15545. https://doi.org/10.1073/pnas.1920498117

Leeper, T. J. (2019). Where Have the Respondents Gone? Perhaps We Ate Them All. *Public Opinion Quarterly*, *83*(S1), 280–288. https://doi.org/10.1093/POQ/NFZ010

MacInnis, B., Krosnick, J. A., Ho, A. S., & Cho, M.-J. (2018). The Accuracy of Measurements with Probability and Nonprobability Survey Samples: Replication and Extension. *Public Opinion Quarterly*, *82*(4), 707–744. https://doi.org/10.1093/poq/nfy038

McDermott, R. (2022). Breaking free. *Politics and the Life Sciences*, *41*(1), 55–59. https://doi.org/10.1017/pls.2022.4

McDougall, J. (2019). Media Literacy versus Fake News: Critical Thinking, Resilience and Civic Engagement. *Media Studies*, *10*(19), 29–45. https://doi.org/10.20901/ms.10.19.2/SUBMITTED

Mercer, A., Kennedy, C., & Keeter, S. (2024). *Online opt-in polls can produce misleading results about young adults', Hispanics' views*. https://www.pewresearch.org/short-reads/2024/03/05/online-opt-in-polls-can-produce-misleading-results-especially-for-young-people-and-hispanic-adults/

MOH | National Population Health Survey 2022. (n.d.). Retrieved July 7, 2024, from https://www.moh.gov.sg/resources-statistics/reports/national-population-health-survey-2022

Pasek, J. (2015). The Polls—Review Predicting Elections: Considering Tools to Pool the Polls. *Public Opinion Quarterly*, *79*(2), 594–619. https://doi.org/10.1093/poq/nfu060

Roozenbeek, J., van der Linden, S., Goldberg, B., Rathje, S., & Lewandowsky, S. (2022). Psychological inoculation improves resilience against misinformation on social media. *Science Advances*, *8*(34). https://doi.org/10.1126/SCIADV.ABO6254

Saleh, N. F., Roozenbeek, J., Makki, F. A., Mcclanahan, W. P., & Linden, S. van der. (2021). Active inoculation boosts attitudinal resistance against extremist persuasion techniques: a novel approach towards the prevention of violent extremism. *Behavioural Public Policy*, 1–24. https://doi.org/10.1017/BPP.2020.60

Shamon, H., & Berning, C. (2020). Attention check items and instructions in online surveys with incentivized and non-incentivizedquality?Samples: Boon or bane for data. *Survey Research Methods*, *14*(1), 55–77. https://doi.org/10.18148/srm/2020.v14i1.7374

Stadtmüller, S., Silber, H., & Beuthner, C. (2022). What Influences Trust in Survey Results? Evidence From a Vignette Experiment. *International Journal of Public Opinion Research*, *34*(2), 1–9. https://doi.org/10.1093/IJPOR/EDAC012

Udry, J., & Barber, S. J. (2024). The illusory truth effect: A review of how repetition increases belief in misinformation. *Current Opinion in Psychology*, *56*, 101736. https://doi.org/10.1016/J.COPSYC.2023.101736

Valentine, J. C., & McHugh, C. M. (2007). The Effects of Attrition on Baseline Comparability in Randomized Experiments in Education: A Meta-Analysis. *Psychological Methods*, *12*(3), 268–282. https://doi.org/10.1037/1082-989X.12.3.268

van der Meer, T. G. L. A., Hameleers, M., & Ohme, J. (2023). Can Fighting Misinformation Have a Negative Spillover Effect? How Warnings for the Threat of Misinformation Can Decrease General News Credibility. *Journalism Studies*, *24*(6), 803–823. https://doi.org/10.1080/1461670X.2023.2187652

Watkins, E. R., & Newbold, A. (2020). Factorial designs help to understand how psychological therapy works. *Frontiers in Psychiatry*, *11*, 477682. https://doi.org/10.3389/fpsyt.2020.00429

Williams-Ceci, S., Macy, M. W., & Naaman, M. (2024). Misinformation does not reduce trust in accurate search results, but warning banners may backfire. *Scientific Reports*, *14*(1), 10977. https://doi.org/10.1038/s41598-024-61645-8

Wood, T., & Porter, E. (2019). The Elusive Backfire Effect: Mass Attitudes' Steadfast Factual Adherence. *Political Behavior*, *41*(1), 135–163. https://doi.org/10.1007/s11109-018-9443-y
